# Multiple Copies of a Simple MYB-Binding Site Confers Trans-regulation by Specific Flavonoid-Related R2R3 MYBs in Diverse Species

**DOI:** 10.3389/fpls.2017.01864

**Published:** 2017-10-31

**Authors:** Cyril Brendolise, Richard V. Espley, Kui Lin-Wang, William Laing, Yongyan Peng, Tony McGhie, Supinya Dejnoprat, Sumathi Tomes, Roger P. Hellens, Andrew C. Allan

**Affiliations:** ^1^Mt Albert Research Centre, Plant and Food Research, Auckland, New Zealand; ^2^Fitzherbert Science Centre, Plant and Food Research, Palmerston North, New Zealand; ^3^Centre for Tropical Crops and Biocommodities, Queensland University of Technology, Brisbane, QLD, Australia; ^4^School of Biological Sciences, University of Auckland, Auckland, New Zealand

**Keywords:** promoter, transcription factor, MYB, biotechnology, flavonoids, anthocyanin

## Abstract

In apple, the MYB transcription factor MYB10 controls the accumulation of anthocyanins. MYB10 is able to auto-activate its expression by binding its own promoter at a specific motif, the R1 motif. In some apple accessions a natural mutation, termed R6, has more copies of this motif within the MYB10 promoter resulting in stronger auto-activation and elevated anthocyanins. Here we show that other anthocyanin-related MYBs selected from apple, pear, strawberry, petunia, kiwifruit and Arabidopsis are able to activate promoters containing the R6 motif. To examine the specificity of this motif, members of the R2R3 MYB family were screened against a promoter harboring the R6 mutation. Only MYBs from subgroups 5 and 6 activate expression by binding the R6 motif, with these MYBs sharing conserved residues in their R2R3 DNA binding domains. Insertion of the apple R6 motif into orthologous promoters of *MYB10* in pear (*PcMYB10*) and Arabidopsis (*AtMY75*) elevated anthocyanin levels. Introduction of the R6 motif into the promoter region of an anthocyanin biosynthetic enzyme F3′5′H of kiwifruit imparts regulation by MYB10. This results in elevated levels of delphinidin in both tobacco and kiwifruit. Finally, an R6 motif inserted into the promoter the vitamin C biosynthesis gene *GDP-L-Gal phosphorylase* increases vitamin C content in a MYB10-dependent manner. This motif therefore provides a tool to re-engineer novel MYB-regulated responses in plants.

## Introduction

Gene transcription is partly controlled by transcription factors (TFs), often in combination(s) and within hierarchical networks (Hobert, 2008). These TFs interact with *cis*-acting sequences, which are typically found in promoters in proximity to the coding sequence of the target gene. TF proteins possess distinct functional domains, such as a DNA binding domain and a transcriptional activator domain. The DNA binding domain is conserved within the separate classes of TF and contain *trans*-acting motifs that bind to the *cis*-acting sequences of target gene promoters. TFs are identified by their distinct DNA binding domains, such as the large family of R2R3 domain MYB TFs (Dubos et al., 2010). Activator domains are not as conserved and are involved in the enhancement or repression of transcription, modulating the rate of mRNA synthesis by RNA polymerase II (Ptashne, 1988; Riechmann, 2002). Transcriptional regulation may depend on the interaction of binding and activator domains of different TFs, or on homo- or hetero-dimerisation of certain classes of TF proteins. TFs may co-ordinate regulation in a combinatorial manner, with different classes of TF forming complexes at the protein:DNA or protein:protein level (Grotewold et al., 2000). As TFs can control the transcription of whole pathways, they have become a target for metabolic engineering (Broun, 2004).

Identifying regulatory elements in plant promoter sequences is critical to understanding the specificity of interaction between TFs and target gene promoters, as well as understanding gene regulatory networks. The most common regulatory elements are between 6 and 15 nucleotides, which are well-conserved between species. These elements provide sites for the binding of different classes of TF proteins onto the regulatory sequences of genes to provide regulation of a particular biosynthetic pathway or transcriptional cascade. These have been characterized at the bioinformatic and functional level, and can be viewed in various databases including TRANSFAC, AGRIS, and PLACE (Wingender et al., 1996; Higo et al., 1999; Davuluri et al., 2003). Well-studied examples include the bZIP protein binding G-box (CACGTG) (Menkens et al., 1995), the MYB motifs (Sablowski et al., 1994; Romero et al., 1998), the CArG box (Riechmann et al., 1996), and combinations of these (Hartmann et al., 2005). More recently, computational approaches (Rombauts et al., 2003) and large sequencing data sets, such as the Arabidopsis 1,001-genomes project (Cao et al., 2011), have been used to expand the knowledge on regulatory elements as well as identifying new motifs (Korkuc et al., 2014).

Occasionally the targeted binding motif of a TF may also be present in the regulatory promoter region of the TF itself, providing a means of auto-regulation. The Arabidopsis MADS-box TF, AGL15, has been shown to auto-regulate its own expression by binding its own promoter sequence at *cis*-elements known to associate with MADS-box binding (Zhu and Perry, 2005). The Arabidopsis TT8 bHLH TF exhibits auto-regulatory activity, partly mediated by members of the MYB, bHLH and WD40 (MBW) complex (Baudry et al., 2006). Further research has shown that the expression of TT8 is controlled by different MBW complexes, which dictate the synthesis of specific flavonoids in different tissues (Xu et al., 2013). In apple the anthocyanin activating R2R3 MYB, MYB10, has been shown to auto-activate its own expression by binding a *cis*-element in its own promoter (Espley et al., 2009). The activity of other R2R3 MYBs has been shown to be under an auto-regulatory control, with the effect sometimes being repressive. Arabidopsis MYB4, whose encoded protein is able to bind its own promoter, suppresses transcription as part of a negative auto-regulatory loop (Zhao et al., 2007). A similar repressive mechanism has been shown for the rice *OsMYB4* gene (Baldoni et al., 2013), petunia MYB27 (Albert et al., 2014) and Arabidopsis AtMYBL2 (Dubos et al., 2008).

The plant MYB superfamily is abundant with over 180 members in Arabidopsis (Stracke et al., 2001) and is functionally diverse, having roles in plant development, secondary metabolism, signal transduction, disease resistance, and stress response (Jin and Martin, 1999; Dare et al., 2008). MYBs are characterized by a structurally conserved N-terminal DNA binding domain consisting of single or multiple imperfect repeats (Stracke et al., 2001). These repeats each code three alpha-helices, the third helix binding directly with the major groove of the DNA target (Stracke et al., 2001). The R2R3 MYB TFs in Arabidopsis have been divided on the basis of their sequence into 24 sub-groups (Stracke et al., 2001). More recent genome-wide studies have continued to elucidate this diverse class of TFs in other species. For example, one of the largest families has been identified in soybean, with 244 R2R3 MYBs (Du et al., 2012), while the *Populus* genome contains 192 R2R3 MYBs (Wilkins et al., 2009). R2R3 MYBs, acting as part of a MBW complex, are involved in flavonoid regulation including anthocyanin biosynthesis (Ramsay and Glover, 2005).

In apple, studies have shown that MYB1 (also called MYBA) is responsible for anthocyanin accumulation in the apple skin, while MYB10 regulates flesh color in some cultivars (Takos et al., 2006; Ban et al., 2007; Espley et al., 2007). Previous studies and new analysis of the recently released double-haploid ‘Golden Delicious’ genome confirms the allelic nature of MYB10 and MYB1/A (Lin-Wang et al., 2010; Daccord et al., 2017). In red-fleshed/red-foliaged apple accessions, the accumulation of anthocyanins correlates with an elevation of MYB10 transcript levels (Espley et al., 2007). In these cultivars there are two alleles; the MYB1 allele, which is also present in all white-fleshed, green-foliaged apples and has one copy of a 23-base pair sequence (R1) in the proximal promoter sequence of the coding sequence of the MYB, and secondly the MYB10 allele, which is only found in red-fleshed, red-foliage apple accessions and contains a further five copies of the 23-base pair sequence in a minisatellite-like structure (Espley et al., 2009). This multiple repeat rearrangement (R6) generates a positive auto-regulatory allele, as MYB10 can bind the 23 bp sequence as shown by *in vitro* binding assays (Espley et al., 2009). This is sufficient to account for the increase in MYB10 transcripts in all red-foliaged apple varieties. Further studies have confirmed that this R6-MYB10 allele is strongly associated with red-fleshed apples (Volz et al., 2009; van Nocker et al., 2011) and crab apples (Tian et al., 2015). The R6 rearrangement appears to be specific to apple as to date it has not been found naturally in red tissues of other species (e.g., plum, peach). The presence of the progenitor R1 motif is less confined, as it appears in other species in the Rosaceae family, such as pear, peach, cherry, and plum. The R6 motif can function in other species: transformation of petunia using a construct containing the apple MYB10 promoter (*MYB10-R6_pro_*) driving the apple MYB10 gene generates increased anthocyanin in petals (Boase et al., 2015).

Here, we demonstrate that anthocyanin-activating R2R3 MYBs from a number of species can activate the apple *MYB10-R6_pro_*. We show that the introduction of an R6 motif into promoters of MYB10 orthologs can initiate auto-regulation in species as diverse as pear and Arabidopsis. Engineering of this *cis*-element can change anthocyanin composition, harnessing the activity of different biosynthetic genes to produce a different metabolic profile. We then engineered the promoter of an enzyme completely unrelated to anthocyanin, the biosynthesis of ascorbic acid (Laing et al., 2015), to generate a gene that is then responsive to the MYB10 protein. Therefore, the R6 motif can be exploited to drive transcription of a range of genes in the presence of MYB10. This range is not restricted to flavonoid biosynthesis. This versatility could suit a range of biotechnological applications for promoter engineering in order to produce a temporal or spatial elevation of a target pathways.

## Results

### MYB10 Orthologs Activate the *MYB10-R6_pro_* Promoter

**FIGURE 1:**
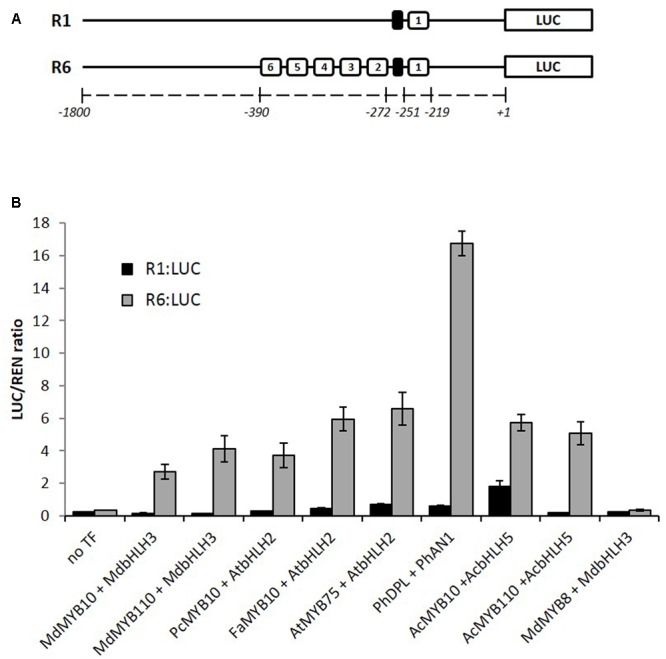
MYB10 orthologs activate the R6-containing *MYB10* promoter in a transient assay in *Nicotiana benthamiana*. **(A)** Schematic of the R1 and R6 apple MYB10 promoters fused to the *LUC* reporter gene in pGreen0800LUC representing the R repeat units (open boxes) and the microsatellite structure upstream of unit 1 (black box) and their position relative to the ATG start codon. **(B)** Leaves of *N. benthamiana* were infiltrated with the *MdMYB10-R1_pro_*:*LUC* or *MdMYB10-R6_pro_*:*LUC* fusions (named R1:LUC and R6:LUC respectively) on their own (no TF) or co-infiltrated with MdMYB10 or MYB10 orthologs and bHLH partner. Md: *Malus domestica*, Pc: *Pyrus communis*, Fa: *Fragaria ananassa*, At: *Arabidopsis thaliana*, Ph: *Petunia hybrida*, Ac: *Actinidia chinensis*. Luminescence was measured 3 days post-infiltration and is expressed as a ratio of the LUC to REN signals. Data represent means (± SE) of four technical replicate reactions.

We have previously reported that apple MYB10 can regulate its own transcription by binding to a characterized motif (R1) in its own promoter (Espley et al., 2009). A naturally occurring mutation of this motif, which has multiple repeats of the MYB binding site (R6), results in enhanced MYB10 auto-regulation and ectopic accumulation of anthocyanin in apple trees. Examination of many sources of apple germplasm (Espley et al., 2009) identified only R1 and R6 variants, suggesting that this mutation has happened only once. To test whether this activation is restricted to the apple MYB10, we over-expressed the anthocyanin-activating MYBs (belonging to subgroup 6, according to Stracke et al., 2001) from pear, strawberry, Arabidopsis, petunia, and kiwifruit in *Nicotiana benthamiana* together with the R1 or R6 versions of the apple MYB10 promoter fused to a luciferase reporter (Hellens et al., 2005). Expression of these MYBs and their respective bHLH partner led to an average 16-fold activation of the R6 variant of the MYB10 promoter compared with R1 (**Figure 1**). Some of the MYBs tested, particularly PhDPL, were able to activate the R6 promoter to a greater extent than MYB10 from apple. In the same experiment, MdMYB8, an R2R3 MYB from apple, which is not implicated in anthocyanin regulation (Espley et al., 2007), failed to activate the R6 motif, even in the presence of a bHLH partner.

### Flavonoid-Related R2R3 MYBs Activate the *MYB10-R6_pro_* Promoter

Anthocyanin-related MYBs from a diverse range of species activated the R6 promoter, whereas MdMYB8, not implicated in anthocyanin regulation, failed to do so. To further test this specificity, the R1 and R6 variants of the *MdMYB10* promoter were screened with a panel of apple R2R3 MYBs. To ensure that we tested MYBs across a range of subgroups (Stracke et al., 2001), all the available apple MYB sequences were extracted from the apple genome database (Velasco et al., 2010). These were then assembled into a phylogenetic tree together with Arabidopsis MYBs. From this, a total of 29 R2R3 MYBs from a diverse range of subgroups were selected.

**FIGURE 2:**
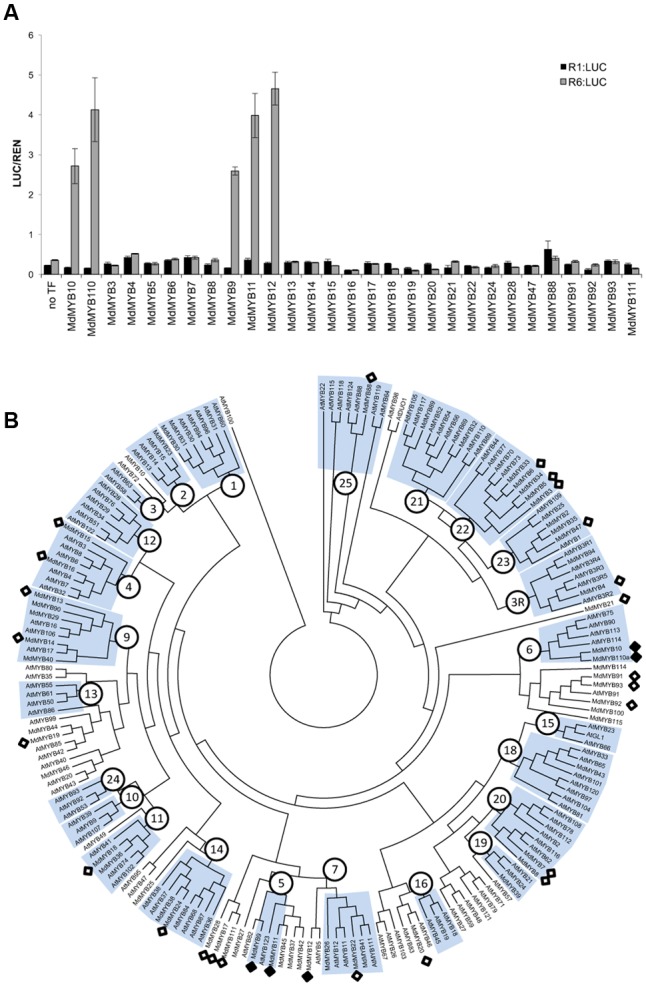
Apple MYBs from subgroups 5, 6 and MdMYB12 can activate the R6-containing *MYB10* promoter. **(A)** Apple MYB transcription factors (TFs) were co-infiltrated with MdbHLH3 and the *MdMYB10-R1_pro_*:*LUC* or *MdMYB10-R6_pro_*:*LUC* fusions (named R1:LUC and R6:LUC respectively) in *N. benthamiana* leaves and the LUC/REN signal was measured. Data represent means (± SE) of four technical replicate reactions. **(B)** Phylogenetic tree representing relationships between apple and Arabidopsis MYBs. Numbers indicate subgroups previously described for Arabidopsis MYBs (Stracke et al., 2001). Apple MYBs tested in transient assay in **(A)** (white diamond) and the MYBs tested positive for transactivation of the R6 promoter (black diamond) are indicated. Deduced amino acid sequences were aligned using CLUSTALX. Protein distances were calculated with PROTDIST using the Jones-Taylor-Thornton matrix and the tree was constructed by the neighbor-joining method.

Among all the apple MYBs tested, only MYB9, MYB10, MYB11, MYB12, and MYB110 were able to activate the *MYB10-R6_pro_* promoter (**Figure 2A**) in the presence of MdbHLH3, suggesting that recognition of the R6 motif was restricted to flavonoid-related MYBs (**Figure 2B**). These MYBs are from subgroup 5 (MdMYB9 and 11), representing the proanthocyanidin MYB clade (e.g., Arabidopsis TT2), subgroup 6 (MdMYB10 and 110) representing the anthocyanin-associated clade (e.g., AtMYB75) and a putative ortholog of AtMYB5 (MdMYB12), which regulates seed mucilage and seed-coat pigmentation (Li et al., 2009).

### Conserved Amino Acid Residues within the R6-Activating MYBs

**FIGURE 3:**
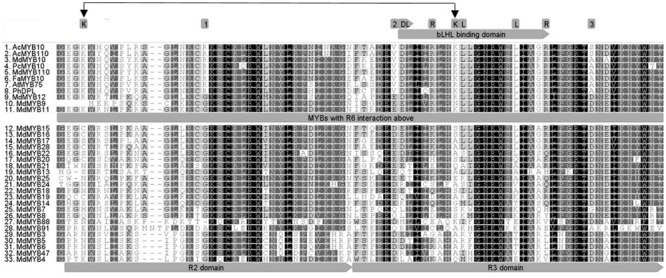
Deduced amino acid sequences were aligned using CLUSTALW. The bHLH binding region within the R3 domain is indicated (gray arrow) and conserved amino acids required for the bHLH-dependant activation (Grotewold et al., 2000) are highlighted above it. Box 1, 2, and 3 show previously described conserved residues specific to dicot anthocyanin-promoting MYBs (Lin-Wang et al., 2010). Double black arrow indicates the signature (K/R)_25_-(X)x46-(K/R/N)_72_ specific to the R6-activating MYBs.

Protein alignment of the R2R3 domain of all the MYBs tested in the transient assay (**Figures 1, 2**), revealed that the flavonoid related MYBs shared a potential amino acid signature in their R2R3 domain consisting of a Lysine or Arginine residue at position 25 and a Lysine, Arginine, or Glutamine residue at position 72, relative to MdMYB10 sequence (**Figure 3**). We also included in our analysis the sequences of other MYBs not necessarily related to flavonoid biosynthesis (**Figure 3**). In these, the signature (K/R)_25_-(X)x46-(K/R/N)_72_ was not apparent at these positions, supporting a specificity linked to flavonoid related MYBs.

### Insertion of the R6 Motif into the Promoters of Pear and Arabidopsis

**FIGURE 4:**
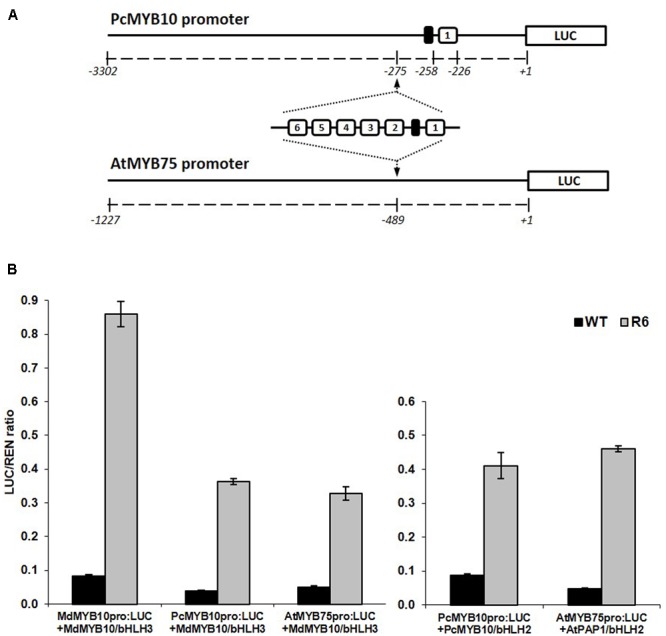
Insertion of the R6 domain in the promoter of MYB10 orthologs leads to autoregulation. **(A)** Schematic of the engineered pear MYB10 and AtMYB75 promoters fused to the *LUC* reporter gene in pGreen0800LUC. **(B)** Dual luciferase promoter assay of the R6 engineered promoters in *N. benthamiana*. Promoters, with or without the R6 domain, were co-infiltrated with MdMYB10 and MdbHLH3, PcMYB10 and AtbHLH2, or AtMYB75 and AtbHLH2. Luminescence of LUC and REN was measured 3 days post-infiltration and expressed as a ratio of LUC to REN. Data represent means (± SE) of four technical replicate reactions.

The specificity of the activation of the R6 motif to MYB10 orthologs and other flavonoid-related MYBs suggests that this motif could be used to elevate plant anthocyanin levels in species other than apple. To test this, the motif was inserted into the promoter of MYB10 orthologs from a close apple relative, European pear (PcMYB10) and an unrelated species, Arabidopsis (AtMYB75). The promoter of PcMYB10 naturally harbors a single repeat of the R1 motif at a similar distance to the start codon as that seen in the apple *MYB10* promoter. The R6 motif was inserted upstream of the predicted 5′ UTR region in the promoter of pear and Arabidopsis, fused to the luciferase reporter (schematic in **Figure 4A**). Transient assays in *N. benthamiana* revealed that both pear and Arabidopsis *MYB* promoters harboring the R6 motif were strongly activated by MdMYB10, PcMYB10, or AtMYB75 (**Figure 4B**). This activation was dependent on a bHLH, either from apple (MdbHLH3) or Arabidopsis (AtbHLH2/EGL3). For the engineered promoters of either pear or Arabidopsis, the level of activation was similar when co-infiltrated with MdMYB10 or with their own MYB activator, PcMYB10 or AtMYB75, respectively.

**FIGURE 5:**
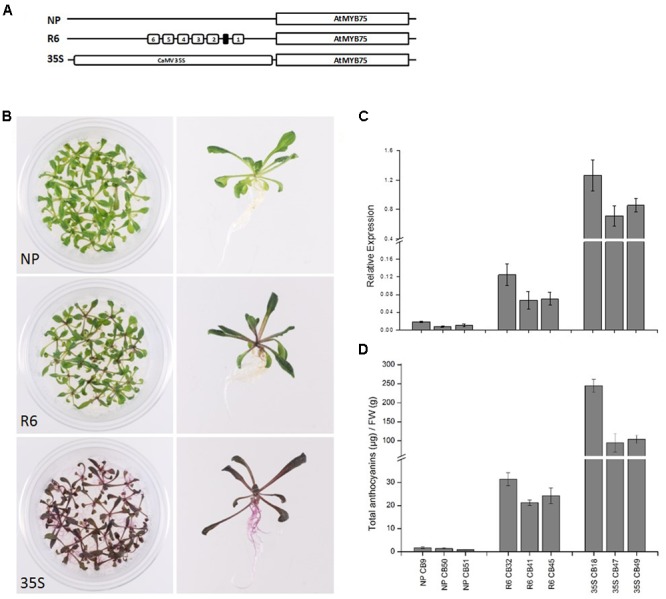
Insertion of the R6 motif into the promoter of AtMYB75 induces AtMYB75 overexpression and ectopic anthocyanin accumulation. **(A)** Schematics of the engineered *AtMYB75* constructs transformed into Arabidopsis. **(B)** Phenotype of representative T2 lines transformed by *AtMYB75_pro_:AtMYB75* [denoted as native promoter (NP)], *AtMYB75-R6_pro_:AtMYB75* (denoted as R6) and *35S_pro_:AtMYB75* (denoted as 35S). **(C)** Expression analysis by qPCR of the transcripts of the *AtMYB75* gene in each transformed lines. Expression is given relative to actin and error bars represent the standard errors of the means calculated from four technical replicates. **(D)** Total anthocyanin content in each lines measured by HPLC and expressed as μg of Cy-glu equivalent per gram of fresh weight (FW).

The engineered promoters harboring the R6 motif were then used to drive the expression of their own respective genes (*PcMYB10* and *AtMYB75*), rather than a luciferase reporter. Three constructs were transformed in Arabidopsis: the native *MYB75* promoter driving *MYB75* genomic DNA (termed *MYB75_pro_:MYB75*), the engineered *MYB75-R6* promoter driving *MYB75* (termed *MYB75-R6_pro_:MYB75*), and a *35S_pro_:MYB75* construct (**Figure 5A**) previously shown to elevate the anthocyanin content in Arabidopsis (Borevitz et al., 2000). Three independent T2 generation lines were generated for each construct. The *MYB75-R6_pro_:MYB75* lines showed enhanced pigmentation compared with *MYB75_pro_:MYB75*, increasing from 1.3 μg/g FW to 26.6 μg/g FW (**Figure 5B**). However, this is not as pigmented as the *35S_pro_:MYB75* lines (**Figure 5B**; 147.8 μg/g FW). Expression analysis of these lines revealed an eightfold increase in *MYB75* expression in the *MYB75-R6_pro_:MYB75* lines compared with *MYB75_pro_:MYB75* (**Figure 5C**). Analysis of anthocyanins in these lines revealed that the total anthocyanin content measured in each line was related to the level of *MYB75* transcription (**Figure 5D**).

**FIGURE 6:**
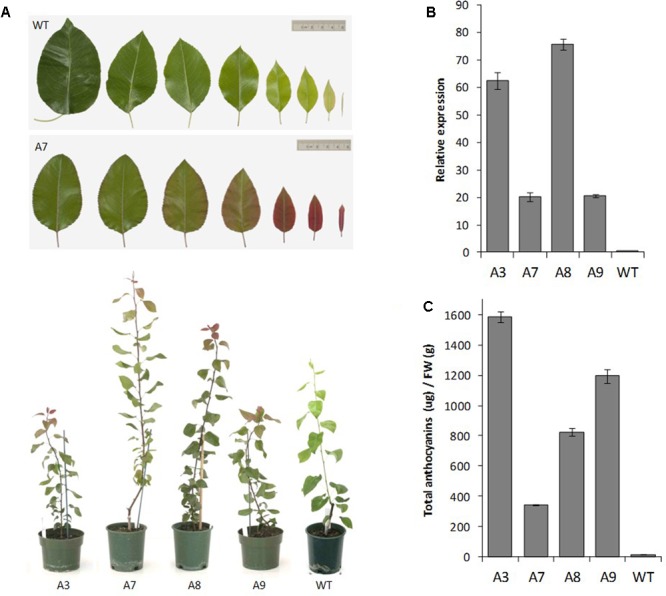
Insertion of R6 domain into the pear *MYB10* promoter induces PcMYB10 overexpression and ectopic anthocyanin accumulation. **(A)** Phenotype of representative transgenic pear lines harboring the *PcMYB10-R6_pro_:PcMYB10* construct. **(B)** Expression analysis by qPCR of the transcripts of the *PcMYB10* gene in fully expanded mature leaves of each transgenic line. Expression is given relative to pear actin and error bars represent the standard errors of the means calculated from four technical replicates. **(C)** Total anthocyanin content of fully expanded mature leaves of each line measured by HPLC and expressed as μg of Cy-glu equivalent per gram of fresh weight (FW). Error bars represent the standard errors of the means calculated from three replicates.

The pear *MYB10* construct engineered to contain the R6 motif was transformed into ‘Conference’ pear. The multiple lines generated showed varying levels of foliar anthocyanin content both visually and using HPLC analysis, whereas no anthocyanin was detected in mature leaves of wild-type pear grown under the same glasshouse conditions (**Figures 6A,B**). The expression of *PcMYB10* was elevated in the presence of the R6 motif compared with the wild-type line (**Figure 6C**). These results showed that the insertion of the R6 motif in the promoter of *AtMYB75* or *PcMYB10* is able to generate an auto-regulatory allele, leading to an ectopic accumulation of anthocyanins in the plant.

### Manipulating Anthocyanin Profile Using the R6 Motif

We examined the potential of using the R6 motif to modify a known biosynthetic pathway by modifying the expression of key genes. The hue of anthocyanin pigments depends on their level of hydroxylation and the position of the hydroxyl group on the B-ring (Harborne, 1967). These hydroxylation steps are directed by P450-dependent monooxygenases, the flavonoid 3′-hydroxylase (F3′H) and the flavonoid 3′5′-hydroxylase (F3′5′H), responsible for the synthesis of cyanidin and delphinidin derivatives respectively. In kiwifruit (*Actinidia* sp.), delphinidin derivatives have been identified in two pigmented species, *A. melanandra* and *A. arguta* var. *purpurea* (Montefiori et al., 2009), but not in commercially grown green (*A. deliciosa*) and yellow or red (*Actinidia chinensis*) fruit.

The promoter region of the *F3*′*5*′*H* gene was isolated from genomic DNA of the yellow kiwifruit and fused to the luciferase reporter gene, with or without a R6 motif inserted 183 bp upstream of the start codon (**Supplementary Figure S1A**). Using a transient assay in *N. benthamiana*, apple MdMYB10 and kiwifruit AcMYB110 (Fraser et al., 2013) were able to enhance the activation of the engineered promoter harboring the R6 motif by threefold compared with the native *F3*′*5*′*H* promoter (**Supplementary Figure S1B**). The engineered R6 promoter was then fused to the genomic DNA of the *F3*′*5*′*H* gene to generate the *F3*′*5*′*H-R6_pro_:F3*′*5*′*H* construct, which was transiently expressed together with *MYB110* in tobacco leaves (*N. tabacum*). Anthocyanin measurements revealed that MYB110 induced a 6.7-fold increase of the Dp/Cy (derivatives) ratio when the R6 motif was inserted in the F3′5′H promoter compared with the native promoter (**Supplementary Figures S1C,D**). MYB110 alone induces synthesis of approximately equal amounts of cyanidin (Cy-glucoside, Cy-rutinoside) and delphinidin derivatives (Dp- rutinoside) in tobacco leaves. The higher Dp/Cy ratio obtained with MYB110 plus *F3*′*5*′*H-R6_pro_:F3*′*5*′*H* construct was due to a reduced amount of cyanidin derivatives, while content of delphinidin derivatives were similar. This could suggest that the enhanced F3′5′H activity competed with the endogenous F3′H of tobacco, hence reducing the amount of substrate available for cyanidins.

**FIGURE 7:**
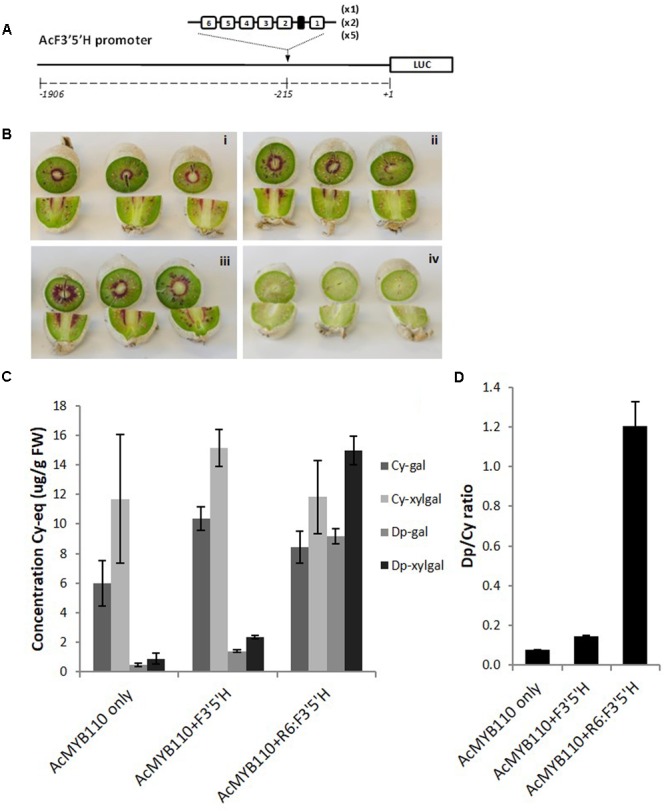
Insertion of the R6 motif into the *AcF3′5′H* promoter induces accumulation of delphinidin derivatives. **(A)** Schematic of the engineered F3*′*5*′*H promoter fused to the *LUC* reporter gene in pGreen0800LUC. **(B)** *Actinidia eriantha* kiwifruit were infiltrated by *Agrobacterium* suspension containing AcMYB110 (i), AcMYB110 and F3*′*5*′*H_pro_: F3*′*5*′*H (ii), AcMYB110 and F3*′*5*′*H-R6_pro_: F3*′*5*′*H (iii) constructs and corresponding empty vectors (iv). Photos were taken 4 days post-infiltration. **(C)** Measurement of cyanidin and delphinidin derivatives in kiwifruits expressing corresponding constructs, expressed as concentration of cyanidin-gal equivalent and **(D)** presented as a ratio of delphinidin to cyanidin.

We also assessed whether the number of repeats had an effect on the magnitude of promoter activation. The kiwifruit *F3*′*5*′*H* promoter was engineered with additional copies of the R6 motif, generating promoters harboring 6, 12, or 30 units of the R1 sequence (**Figure 7A**). These promoters, fused to the luciferase reporter or to the *F3*′*5*′*H* genomic coding sequence, were tested in *N. benthamiana* and tobacco. The luciferase reporter assay suggested that maximum activation is obtained with 12 to 30 repeats, while the Dp/Cy ratio measured in tobacco leaf showing 12 repeats gave the highest ratio (**Supplementary Figure S2**).

To validate this result in kiwifruit tissue, the same constructs were assayed by injection of *Agrobacterium* carrying cloned genes into *A. eriantha* fruits (**Figure 7B**) (Montefiori et al., 2011). Fruits expressing MYB110 alone produced cyanidin-based compounds and trace amounts of delphinidin-galactoside and delphinidin-xylgalactoside (**Figure 7C**). Co-infiltration of the *F3*′*5*′*H_pro_:F3*′*5*′*H* construct slightly enhanced delphinidin derivative levels. However, a significant increase of delphinidins was measured when the engineered *F3*′*5*′*H-R6_pro_:F3*′*5*′*H* was co-infiltrated, yielding to a Dp/Cy ratio 8 times higher than with the native promoter (**Figure 7D**).

### Elevating Ascorbate Content in Plants Using the R6 Motif

Ascorbate (or vitamin C) is an essential metabolite for most living organisms. The GDP-L-galactose phosphorylase, encoded by the *GGP* gene (also known as *VTC2*) has been shown to be the main control point of the pathway (Bulley et al., 2009; Laing et al., 2015). This gene is regulated transcriptionally during fruit development, but also post-transcriptionally *via* a highly conserved uORF within its 5′ UTR region that represses its translation under high ascorbate concentration (Laing et al., 2015).

**FIGURE 8:**
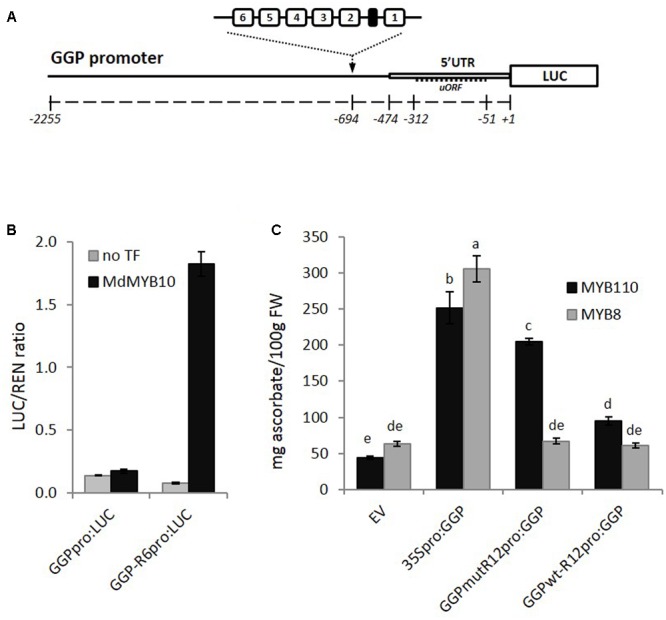
Effect of inserting the R6 motif in the *GGP* promoter. **(A)** Schematic of the engineered *GGP* promoter fused to the *LUC* reporter gene in pGreen0800LUC representing the position of two R6 insertions upstream of the 5*′* UTR and uORF (open box and dash line respectively). **(B)** Dual luciferase assay of the *GGP* promoter harboring the R6 motif fused to the *LUC* reporter. Promoter constructs were co-infiltrated in *N. benthamiana* with MdMYB10 and MdbHLH3. Luminescence of LUC and REN was measured 3 days post-infiltration and expressed as a ratio of LUC to REN. Data represent means (± SE) of four replicate reactions. **(C)** The *GGP* cDNA, placed under its own promoter engineered with two copies of the R6 motif and harboring or not a single point mutation of the uORF start codon (*GGPmut-R12_pro_:GGP* and *GGPwt-R12_pro_-GGP*, respectively), or placed under the control a *35S* promoter (*35S_pro_:GGP*), or the corresponding empty vector (EV) were co-infiltrated with AcMYB110 (or the MYB8 negative control). Ascorbate content was measured at 5 dpi. Data represent means (± SE) of six biological replicates. One-way ANOVA test was applied and resulting grouping information was obtained using the Tukey method, columns sharing the same letters are not significantly different at *P* < 0.05.

The GGP promoter sequence was isolated from kiwifruit (*A. eriantha*), fused to the LUC reporter gene and the R6 motif was inserted upstream of the 5′ UTR (**Figure 8A**). Transient expression in *N. benthamiana* revealed that MdMYB10 was able to activate the *GGP* promoter harboring the R6 motif (**Figure 8B**). To test for any potential increase in ascorbate levels, further assays were performed in *N. benthamiana* using the *CaMV35S* promoter driving *GGP* and a version of the *GGP* promoter engineered with two copies of the R6 motif fused to the *GGP* cDNA (named *GGPwt-R12_pro_:GGP*). These were co-infiltrated in the presence of either the kiwifruit MYB110 or the apple MYB8 as a control. Ascorbate content of the infiltrated patch revealed that the *GGP* construct harboring the R12 motif (*GGPwt-R12_pro_:GGP*) did not increase the ascorbate content (**Figure 8C**). This is likely due negative feedback repression by ascorbate involving the uORF in the 5′ UTR of the *GGP* promoter (Laing et al., 2015). It was shown recently that a single point mutation of the non-canonical start codon of the uORF was sufficient to cancel this negative feedback (Laing et al., 2015). Therefore, we made the same point mutation in the two versions of the *GGP* promoter, either with or without the R12 motif. The construct harboring the R12 motif and the mutated uORF (*GGPmut-R12_pro_:GGP*) was able to induce over-expression of GGP in the presence of MYB110, resulting in a fivefold increase in ascorbate content in tobacco leaves (**Figure 8C**). In the presence of control MdMYB8, no increase in ascorbate concentration was observed. The *35S*-driven construct was able to elevate ascorbate levels, irrespective of MYB co-infiltration.

## Discussion

The transcriptional regulation of endogenous genes and pathways by TFs is one key approach for plant biotechnology. The promoters of targeted genes must respond to the TF, which is dependent on the presence of *cis*-regulatory elements. Synthetic promoter engineering is designed to produce promoters that offer stronger and more precisely targeted transcription (Venter, 2007). Engineering is still reliant on known *cis*-elements, hence the discovery of new enhancer or repressor elements is important. A successful product will also depend on the social and regulatory issues surrounding emerging new gene synthesis and editing technologies, and the outcome of the *trans*-genesis versus *cis*-genesis debate (Holme et al., 2013; Doudna and Charpentier, 2014). Here we describe a number of applications for the R6 motif from apple, a naturally occurring mutation, which produces enhanced binding for specific MYB TFs. This can be used to upregulate biosynthetic pathways or manipulate metabolite composition, with potential for either *cis-*genic adoption in apple and pear, or gene editing technologies in other -species. Here we describe diverse applications, to increase anthocyanin levels (e.g., pear which often suffers from reduced color), alter anthocyanin profile (e.g., kiwifruit, a relatively un-domesticated crop to add novelty and potential health targets), or enhance other metabolites (e.g., vitamin C, a major health target).

Apple MYB10 is a member of a distinct clade of dicot R2R3 MYBs that activate the expression of anthocyanin biosynthetic genes. MYB10 is able to auto-activate its expression by binding its own promoter at an R1 motif, with a natural mutation of this motif, termed R6, resulting in stronger auto-activation. MYB10 orthologs from Arabidopsis, petunia and kiwifruit, representing the Brassicaceae, Solanaceae and Actinidiaceae families, also activate R6. Petunia MYB *DPL*, in particular, was able to trans-activate the apple promoter to a high level. Corresponding *cis*-elements in these species, which resemble R1 or R6-like motifs, were seen in promoters of apple, pear and *Prunus* species, but less evident outside the Rosaceous family (**Supplementary Figure S3**). Activation required the co-infiltration of a bHLH partner.

In a number of plant species, ectopic expression of anthocyanin accumulation has been observed and is associated with altered expression of an anthocyanin-activating MYB. In cauliflower, a Harbinger DNA transposon insertion into the promoter of *BoMYB2* causes ectopic anthocyanin accumulation in the curd (Chiu et al., 2010). In red-foliaged plum (*Prunus cerasifera*) *PcMYB10.6* is constitutively up-regulated and is responsible for this phenotype (Gu et al., 2015). A similar, but independent event is responsible for red foliage in peach (*P. persica*) where *PpMYB10.4* (a paralogous gene) is mis-regulated (Zhou et al., 2014). However, none of these phenotypes is the result of an R6-like mutation. Despite this, the apple motif is recognized by MYBs from other species.

Previous studies show the specificity of certain subgroups of R2R3 MYBs for regulation of specific flavonoids, such as in Arabidopsis (Stracke et al., 2001), peach (Ravaglia et al., 2013) and reviewed in Lai et al. (2013). These include a number of conserved features including the bHLH binding domain (Grotewold et al., 2000) and residues that specify the regulation of either anthocyanins or proanthocyanidins (Heppel et al., 2013). This latter study provided an understanding into the evolutionary aspects of different regulators from a common ancestral MYB from functional analysis of residues within the R2R3 domain. Of the 29 apple MYBs tested across a range of phylogenetic clades, only those belonging to known flavonoid-related subgroups were able to activate the R6 motif. No activation was observed for the previously reported apple anthocyanin repressors, such as MdMYB111 or MdMYB17 (SG4) (Lin-Wang et al., 2010) or for the flavonol regulator MdMYB22 (SG7) (Peng et al., 2013). A consensus signature of (K/R)_25_-Nx46-(K/R/N)_72_ is present in MYBs that trans-activated the R6 motif. This suggests evolution of key amino acids specifying DNA motif recognition. The mechanism for this should be examined in further studies.

We tested R6 in stable transformations of both pear and Arabidopsis to confirm their effect on anthocyanin biosynthesis. The Arabidopsis promoter containing R6 fused to the AtMYB75 coding sequence was transformed into Arabidopsis (**Figure 5**). The stable R6-containing lines showed increases in anthocyanin, which was confirmed by HPLC analysis. The transcription level of AtMYB75 was also clearly elevated in these lines. Transformation of pear with an engineered version of the PcMYB10 gene containing the R6 motif produced trees with ectopic anthocyanin accumulation in the leaves. Both PcMYB10 transcription and anthocyanin concentration were elevated, compared with undetectable levels in WT tissue. This is the first reported case of manipulation of pear pigmentation using transformation.

The function of MYB10 is to bind the promoters of the genes encoding enzymes of the anthocyanin biosynthetic pathway to control pigment levels. This has previously been shown to occur through MYB binding sites (Sablowski et al., 1994). To determine if insertion of an R6 motif would enhance the binding capacity of MYB10 to biosynthetic promoters, the promoter of the F3′5′H from kiwifruit was engineered. Trans-activation in tobacco occurred with both the apple and kiwifruit anthocyanin-related MYBs (when co-infiltrated with a bHLH), which was increased 12-fold after insertion of an R6 motif compared with the background activity of the promoter. Despite this R6-driven increase, the total concentration of anthocyanin in the tobacco leaf remained relatively constant. However, the anthocyanin composition was changed: infiltration with the *F3*′*5*′*H-R6_pro_:F3*′*5*′*H* or *35S_pro_:F3*′*5*′*H* produced a shift from cyanidin derivatives to delphinidin derivatives. This is consistent with the function of F3′5′H (Seitz et al., 2007). The greatest change in ratio was achieved with the R6 engineering, suggesting that this is an efficient way of re-directing anthocyanin composition. Transient assays of F3′5′H were performed in fruit tissue of kiwifruit (**Figure 7**). We compared the effect of infiltrating *AcMYB110* alone or with the endogenous kiwifruit *F3*′*5*′*H* promoter driving the *F3*′*5*′*H*, or with the R6-containing *F3*′*5*′*H* promoter driving the *F3*′*5*′*H*. There was an increase in total anthocyanin concentration with *F3*′*5*′*H-R6_pro_: F3*′*5*′*H* together with a dramatic shift from cyanidin derivatives to delphinidin derivatives. Several studies have targeted the content of cyanidin and delphinidin derivatives in plants in order to modify color, which is usually achieved by either overexpressing or silencing the F3′5′H enzyme, such as in carnations (Fukui et al., 2003), roses (Katsumoto et al., 2007), and tobacco (Okinaka et al., 2003). The color of fruit is also of interest to plant breeders and being able to modify the pigment content in fruit skin or flesh is a valuable tool for breeding new varieties, such as in apple (Volz et al., 2009).

To test the R6 motif in a completely unrelated biosynthetic pathway, we engineered the vitamin C pathway gene, *GGP*, to include the R6 motif. Humans lack the ability to synthesize vitamin C and rely on ingestion from plants. Being able to increase ascorbate content in plants has become an important target for plant breeding. Therefore, the ascorbate biosynthetic pathway and its regulation have been extensively studied (Bulley and Laing, 2016). Vitamin C levels regulate translation of the key gene, *GGP*, via a repression at a uORF within the 5′ UTR of the gene (Laing et al., 2015). Insertion of an R6 into the *GGP* promoter was ineffectual while this uORF was intact. However, when the uORF was disrupted by a point mutation in the non-canonical start site, vitamin C levels were highly elevated in tobacco leaves when AcMYB110 was co-infiltrated with the R6-containing *GGP* promoter. This shows that the ability of the MYB protein to bind promoter sequences harboring R6 is not restricted to flavonoid-related genes and supports the hypothesis that MYB10 can enhance the transcription level of any gene harboring an R6 motif.

Here, we have provided data to show that the R6 sequence is an adaptable motif for the elevation of transcription. This elevation will be concomitant to the expression of a MYB10-like gene, which can provide a temporal-spatial dimension since MYB10 is likely to be elevated itself by environmental and developmental cues, such as the onset of fruit ripening in specific skin cells. Enhanced color in apple and pear could be achieved via *cis*-genesis, or gene editing. This could enhance potential health components, as evidence suggests that a high-anthocyanin diet may be beneficial (Espley et al., 2014), and that some anthocyanins are more bioactive in the diet than others (Nyanhanda et al., 2014). Other phytochemicals related to health, flavor or other quality parameters could be increased concurrent with color change, such as vitamin C concentration. In summary, the R6 motif is a novel and versatile tool for biotechnological approaches for plant manipulation.

## Experimental Procedures

### Plant Material and Transformation

Arabidopsis plants (Columbia-0) were transformed using the floral dip method (Clough and Bent, 1998). Resultant transgenic seeds were sterilized in 70% ethanol – 0.05% Triton X-100 for 10 min, then in 100% ethanol twice for 5 min each time. Seeds were dried on Whatman filter paper, then sprinkled onto growth media containing ½ MS + 2% sucrose + 50 mg/L kanamycin and placed at 25°C with 16 h photoperiod. At least three independent T2 generation lines were selected for analysis. Four-week-old transgenic seedlings were harvested and frozen in liquid nitrogen, ground into powder and stored at -80°C for HPLC and qPCR analysis.

*Nicotiana benthamiana* and *N. tabacum* were grown in a glasshouse at 22–25°C with natural light and supplemental lighting to extend the day length to 16 h.

Pear plants, cv. ‘Conference,’ were grown *in vitro* in apple multiplication media. Freshly opened leaves from 4 weeks old plants in tissue culture were used as explants for *Agrobacterium*-mediated transformation based on the apple protocols (Yao et al., 2013). The vitamins in the apple protocols were replaced with the vitamin as described by Brisset et al. (1988). Transgenic plants were rooted and grown in a containment glasshouse at The New Zealand Institute for Plant and Food Research Limited (PFR), Auckland, New Zealand.

### Plasmid Construction

Apple MYB10-R1_pro_ and MYB10-R6_pro_ promoters cloned in the pGreen0800LUC vector (Hellens et al., 2005) as a transcriptional fusion to the luciferase gene were previously described (Espley et al., 2009). Other constructs expressing the apple TFs and other MYB10 orthologs under the control of the CaMV35S promoter have also been described previously: *MdMYB10, MdbHLH3, MdMYB8* (Espley et al., 2007), *MdMYB110* (Chagne et al., 2013), *PcMYB10, FaMYB10* and *AtbHLH2*/At1g63650/*EGL3* (Lin-Wang et al., 2010), *PhDPL* (Albert et al., 2011), *AtMYB75, MdMYB4, MdMYB6, MdMYB7, MdMYB11, MdMYB13, MdMYB14, MdMYB18, MdMYB19, MdMYB20, MdMYB21, MdMYB22, MdMYB24, MdMYB91, MdMYB92* (Hellens et al., 2005), *MdMYB15, MdMYB16, MdMYB17, MdMYB111* (Lin-Wang et al., 2011), *AcMYB110* (Montefiori et al., 2015). GenBank^®^ accessions for the TFs which were cloned into the pSAK277 vector under the control of the CaMV 35S promoter and not previously reported are as follows: Kiwifruit *bHLH5* (KY623715), *MdMYB3* (ADL36760.1), *MdMYB5* (ADL36769.1), *MdMYB9* (ABB84757.1), *MdMYB12* (ADL36755.1), *MdMYB28* (KY623714), *MdMYB47* (ABK20308.1), *MdMYB88* (ADL36771.1), *MdMYB93* (ADL36772.1).

### Heterologous Promoter Engineering

Promoters were engineered using standard restriction cloning of the PCR amplified R6 fragment from apple. A 1227 bp fragment of the AtMYB75 promoter was cloned into pGreen0800LUC in which the *Hind*III restriction site had been disrupted by digestion, T4 DNA polymerase treatment and re-ligation. The R6 motif was amplified from the R6:MYB10 genomic construct (Espley et al., 2009) using primers FW01 and RV01 (**Supplementary Table S1**), the PCR product was digested by *Dra*I, the 194 bp fragment was gel purified, treated by T4 DNA polymerase and ligated into pGreen0800LUC-AtMYB75_pro_ at the *Hind*III site located 489 bp upstream of the start codon to generate the AtMYB75-R6_pro_-LUC construct. The AtMYB75 promoters with and without the R6 insert were then excised from the pGreen0800-LUC (*Kpn*I/*Nco*I) and used to replace the 35S promoter in the pGreenII 62-SK-AtMYB75 (35S-AtMYB75) to generate the R0-AtMYB75 and R6-AtMYB75 constructs.

A 3.3 kb fragment of the *PcMYB10* promoter and the full-length PcMYB10 gene, including 3.3 kb of promoter and 1.5 kb of terminator, were amplified from ‘Williams Bon Chretien’ pear genomic DNA using primers FW02/RV02 and FW02/RV03, and cloned into pGreen0800LUC and in pGEMTeasy, respectively. The 194 bp R6 fragment isolated as previously was ligated at the *Bsg*I restriction site located at position -275 within the PcMYB10 promoter in the pGreenLUC and in the pGEMTeasy constructs. The full-length PcMYB10 alleles with and without the R6 insert were then sub-cloned at the *Not*I site into the pART27 binary vector to generate the R1-PcMYB10 and R6-PcMYB10 constructs.

A full-length *F3′5′H* gene, including 1906 bp of promoter and 780 bp of terminator was amplified from kiwifruit (*A. chinensis*) genomic DNA using primers FW03 and RV04 and cloned into pENTR-D/TOPO following instructions from the manufacturer (Invitrogen, Carlsbad, CA, United States). The plasmid was linearised at position -215 in the *F3′5′H* promoter by inverse PCR using primers FW04 and RV05 and one, two and five copies of the R6 fragment, isolated as described earlier, was ligated within the promoter, hence generating new R6-, R12-, and R30- *F3′5′H* versions. The *CaMV35S* promoter region and nopaline synthase 3*′* region were deleted from the pHEX2 vector (Hellens et al., 2005) to generate the pXCB2 vector in which the native (R0), R6, R12, and R30 alleles of *F3′5′H* were recombined by LR reaction following manufacturer instructions (Invitrogen). The promoter region of the four *F3′5′H* alleles was amplified using primers FW03 and RV06 and cloned at the *Bam*HI-*Nco*I sites fused to the LUC reporter in the pGreen0800LUC vector.

Cloning of the kiwifruit *GGP* promoter into pGreen0800LUC has been described previously (Laing et al., 2015). The R6 fragment isolated as described above was ligated at the *Hpa*I restriction site located at position -694 within the *GGP* promoter. Positive clones were sequenced and a clone containing two tandem copies of the R6 fragment was selected (R12). The *GGP* promoter containing the R12 domain was fused to the *GGP* coding region by overlap PCR extension. The *GGP* promoter was amplified using primers OE3/OE4 and the *GGP* coding sequence was amplified from the pHEX2-*GGP* construct (Bulley et al., 2012) using OE1/OE2. Equal amount of the two gel-purified PCR products were mixed and used as template to amplify the full-length *GGP* allele using primers OE3/OE2, which was cloned into the pGreen0000 vector (Hellens et al., 2005) at the *Kpn*I site. The non-canonical start codon of the uORF within the 5′ UTR of the GGP promoter was mutated from ACG to TTG by inverse PCR of the pGreen0000-R12:*GGP* construct using primers M1/M2 both containing the mutated TTG codon encompassed in an 18 bp overlap. Linearised mutated amplicon was re-ligated using In-Fusion HD enzyme (Clontech Laboratories, Inc., Mountain View, CA, United States) following instructions from the manufacturer.

### Phylogenetic Analysis

The phylogenetic analysis was conducted using the PHYLIP suite of programs (Felsenstein, 1989). Full-length deduced amino acid sequences of 132 and 58 members of the MYB family from Arabidopsis and apple, respectively (sequences provided in Supplementary Date Sheet 1) were aligned using CLUSTALX (Thompson et al., 1997) and protein distances were calculated with PRODIST using the Jones-Taylor-Thornton matrix. Finally, the phylogenetic tree was generated by the neighbor-joining method and visualized using MEGA 4.0 software (Tamura et al., 2007).

### Transient Expression Assays

The dual luciferase promoter assays in *N. benthamiana* and transient expression experiments in *N. tabacum* of the F3′5′H and MYB constructs were performed as previously described (Espley et al., 2007). Tobacco infiltrated leaf areas were harvested 5 days after infiltration and cyanidin and delphinidin derivatives were analyzed by LC–MS. Transient expression in *A. eriantha* fruits was performed as described previously (Montefiori et al., 2011). Four mature fruits were syringe-injected with *Agrobacterium* suspensions containing the combination of constructs as indicated in the tables and figures. Tissue from the area of injection showing anthocyanin accumulation was collected at 6 days post-injection and anthocyanin was extracted and analyzed by LC–MS. For ascorbate concentration experiments, *N. tabacum* leaves were co-infiltrated with equal amounts of *Agrobacterium* suspensions containing the GGP and the MYB constructs together with a *35S_pro_:GME* (GDP-mannose-3′,5′-epimerase) construct shown previously to synergistically enhance the ascorbate production by GGP (Bulley et al., 2009) and the suppressor of silencing P19, following a procedure previously described (Laing et al., 2015). Ascorbate content in leaf tissue was measured by HPLC as described in Rassam and Laing (2005).

### HPLC and LC–MS Analysis

For each Arabidopsis line, three seedlings were snap-frozen in liquid nitrogen, powdered and freeze-dried overnight in the dark. The freeze-dried samples were then re-suspended in 100% methanol + 0.1% HCl and incubated at room temperature for 2 h in the dark with occasional vortexing. The samples were centrifuged and supernatants were spin dried for 2 h, re-suspended thoroughly in 20% methanol, centrifuged again and the supernatants were analyzed by LC–MS (Supplementary Data Sheet 2).

For each pear line, three mature fully expanded leaves were snap-frozen, powdered, and freeze-dried overnight. Samples were then extracted overnight at 1°C with 2.5 mL solvent (ethanol/water/formic acid 80/20/1 v/v/v). After the samples were centrifuged (Jouan, microcentrifuge and diluted with methanol/water/formic acid 50/50/5 v/v/v) the samples were analyzed by UHPLC as detailed in Supplementary Data Sheet 2.

*Nicotiana tabacum* leaf samples and *A. eriantha* fruit samples expressing F3′5′H constructs were harvested, snap-frozen in liquid nitrogen and freeze-dried overnight, then re-suspended in methanol + 0.1% HCl and incubated at room temperature for 2 h and analyzed by UHPLC (Supplementary Data Sheet 2).

### Real-time Quantitative PCR (q-PCR) Analysis

RNA from the 27 transgenic Arabidopsis samples and the 15 pear samples (fully expanded mature leaves) used for anthocyanin extractions were extracted by using Spectrum Plant Total RNA Kit (Sigma-Aldrich, St. Louis, MO, United States). First-strand cDNA synthesis was carried out by using RT Primer Mix according to manufacturer’s instructions (QuantiTect Reverse Transcription kit, Qiagen, Hilden, Germany). Real-time qPCR DNA amplification and analysis was carried out using the LightCycler 480 Real-Time PCR System (Roche Diagnostics, Mannheim, Germany), with LightCycler 480 software version 1.5. The LightCycler 480 SYBR Green I Master Mix (Roche) was used, and 10 μl of total reaction volume applied in all the reactions following the manufacturer’s method. qPCR conditions were 5 min at 95°C, followed by 40 cycles of 5 s at 95°C, 5 s at 60°C, and 10 s at 72°C, followed by 65–95°C melting curve detection. The qPCR efficiency of each gene was obtained by analysing the standard curve of a cDNA serial dilution of that gene. Sequence of the primers used are provided in Supplementary Table S1.

## Author Contributions

AA, RE, CB, and RH conceived the project and its components. CB, KL-W, WL, and SD cloned constructs and performed transient transformations. ST generated pear lines. CB and KL-W conducted gene expression analysis. TM, WL, and YP grew Arabidopsis lines and performed HPLC. AA and RE performed bioinformatics. CB, AA, and RE wrote the paper.

## Conflict of Interest Statement

The authors declare that the research was conducted in the absence of any commercial or financial relationships that could be construed as a potential conflict of interest.

## Supplementary Material

FIGURE S1 Effect of the insertion of the R6 motif into the AcF3′5′H promoter. **(A)** Schematic of the engineered F3′5′H promoter fused to the *LUC* reporter gene in pGreen0800LUC. **(B)** Dual luciferase promoter assay in *N. benthamiana*. Promoters, with or without the R6 motif, were co-infiltrated with MdMYB10 and MdbHLH3, or AcMYB110 alone. Luminescence of LUC and REN was measured 3 days post-infiltration and expressed as a ratio of LUC to REN. Data represent means (± SE) of four technical replicate reactions. **(C)** Transient expression in *N. tabacum* of the F3′5′H gene placed under the control of its own promoter (*F3*′*5*′*H_pro_:F3*′*5*′*H*), the R6-engineered F3′5′H promoter (*F3*′*5*′*H-R6_pro_:F3*′*5*′*H*) or the 35S promoter (*35S_pro_:F3*′*5*′*H*), in presence of AcMYB110. Cyanidin and delphinidin derivatives extracted from tobacco leaves are expressed as concentration of Cy-glu equivalent and **(D)** presented as a ratio of delphinidin to cyanidin.

FIGURE S2 The number of R1 motifs affect the level of transactivation by MYB110. **(A)** *F3*′*5*′*H* promoter, containing 0, 6, 12, or 30 repeats of the R1 motif, fused to the *LUC* reporter was co-infiltrated with MYB110 in *N. benthamiana*, and the LUC/REN ratio was calculated as previously. **(B)** Cyanidin and delphinidin derivatives extracted from tobacco leaves expressing the corresponding engineered promoters driving the F3′5′H gene were quantified by HPLC and are presented as a ratio of delphinidin to cyanidin (Dp/Cy).

FIGURE S3 ClustalW alignment of the R1 motif from Rosaceous species, Arabidopsis and Actinidia **(A)**. Box represents required nucleotides for MYB10 binding (Espley et al., 2009). Graphical representation of the conserved residues at each position of the R1 motif was created in WebLogo (Crooks et al., 2004) **(B)**, the height of each letter representing the relative frequency at that position.
