# Supplementary material for: Multiple Copies of a Simple MYB-Binding Site Confers Trans-regulation by Specific Flavonoid-Related R2R3 MYBs in Diverse Species
Source: Front Plant Sci. 2017 Oct 31;8:1864. doi: 10.3389/fpls.2017.01864 (PMC5671642; doi:10.3389/fpls.2017.01864)
Supplement: FIGURE S1 — Effect of the insertion of the R6 motif into the AcF3′5′H promoter. (A) Schematic of the engineered F3′5′H promoter fused to the LUC reporter gene in pGreen0800LUC. (B) Dual luciferase promoter assay in N. benthamiana. Promoters, with or without the R6 motif, were co-infiltrated with MdMYB10 and MdbHLH3, or AcMYB110 alone. Luminescence of LUC and REN was measured 3 days post-infiltration and expressed as a ratio of LUC to REN. Data represent means (± SE) of four technical replicate reactions. (C) Transient expression in N. tabacum of the F3′5′H gene placed under the control of its own promoter (F3′5′Hpro:F3′5′H), the R6-engineered F3′5′H promoter (F3′5′H-R6pro:F3′5′H) or the 35S promoter (35Spro:F3′5′H), in presence of AcMYB110. Cyanidin and delphinidin derivatives extracted from tobacco leaves are expressed as concentration of Cy-glu equivalent and (D) presented as a ratio of delphinidin to cyanidin. [file Supplementary_Figures.PPTX]

## Slide 1
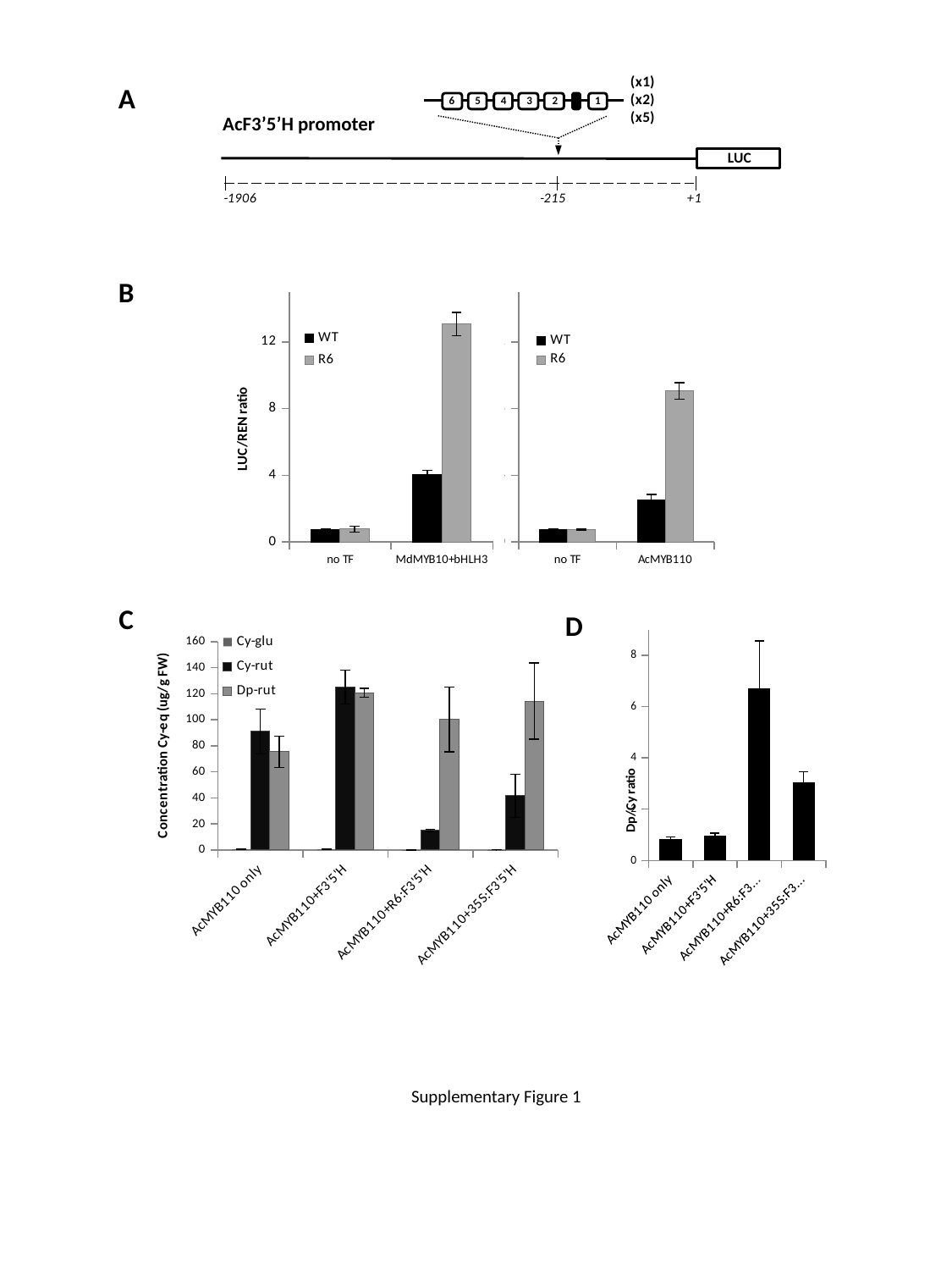

A
AcF3’5’H promoter
B
### Chart
| Category | WT | R6 |
|---|---|---|
| no TF | 0.755707745926581 | 0.7716185972210364 |
| MdMYB10+bHLH3 | 4.056054729059978 | 13.060883204457935 |
### Chart
| Category | WT | R6 |
|---|---|---|
| no TF | 0.7368557902174626 | 0.7365753728129458 |
| AcMYB110 | 2.534467367811129 | 9.06004924144344 |C
D
### Chart
| Category | Dp/Cy |
|---|---|
| AcMYB110 only | 0.8349643332689688 |
| AcMYB110+F3'5'H | 0.9777548196981757 |
| AcMYB110+R6:F3'5'H | 6.7035782262675845 |
| AcMYB110+35S:F3'5'H | 3.0330956460185186 |
### Chart
| Category | Cy-glu | Cy-rut | Dp-rut |
|---|---|---|---|
| AcMYB110 only | 0.6583941420958919 | 91.0709508147084 | 75.41356857127937 |
| AcMYB110+F3'5'H | 0.6329813324944883 | 125.07040379286626 | 120.75951097989022 |
| AcMYB110+R6:F3'5'H | 0.02493853578109031 | 15.092980027607975 | 100.19926159544518 |
| AcMYB110+35S:F3'5'H | 0.1470723982297472 | 41.5909927452096 | 114.33379562193466 |Supplementary Figure 1

## Slide 2
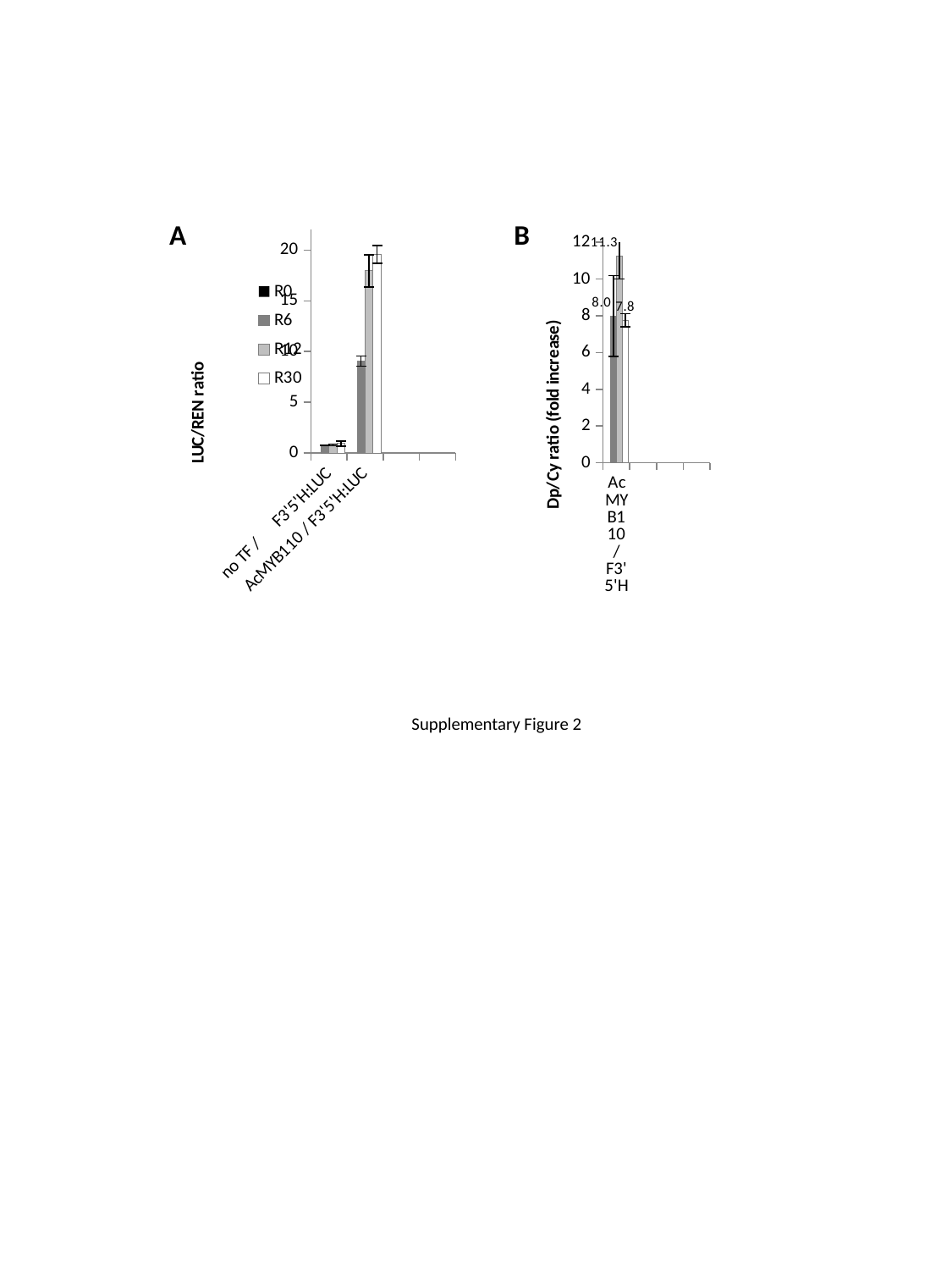

A
B
### Chart
| Category | R0 | R6 | R12 | R30 |
|---|---|---|---|---|
| no TF / F3'5'H:LUC | 0.7368557902174626 | 0.7365753728129458 | 0.7946345051444742 | 0.9203644991795972 |
| AcMYB110 / F3'5'H:LUC | 2.534467367811129 | 9.06004924144344 | 17.956501879261506 | 19.58034611832812 |
### Chart
| Category | R0 | R6 | R12 | R30 |
|---|---|---|---|---|
| AcMYB110 / F3'5'H | 1.169131519525512 | 7.982837163385761 | 11.262973305928888 | 7.75548077718977 |Supplementary Figure 2

## Slide 3
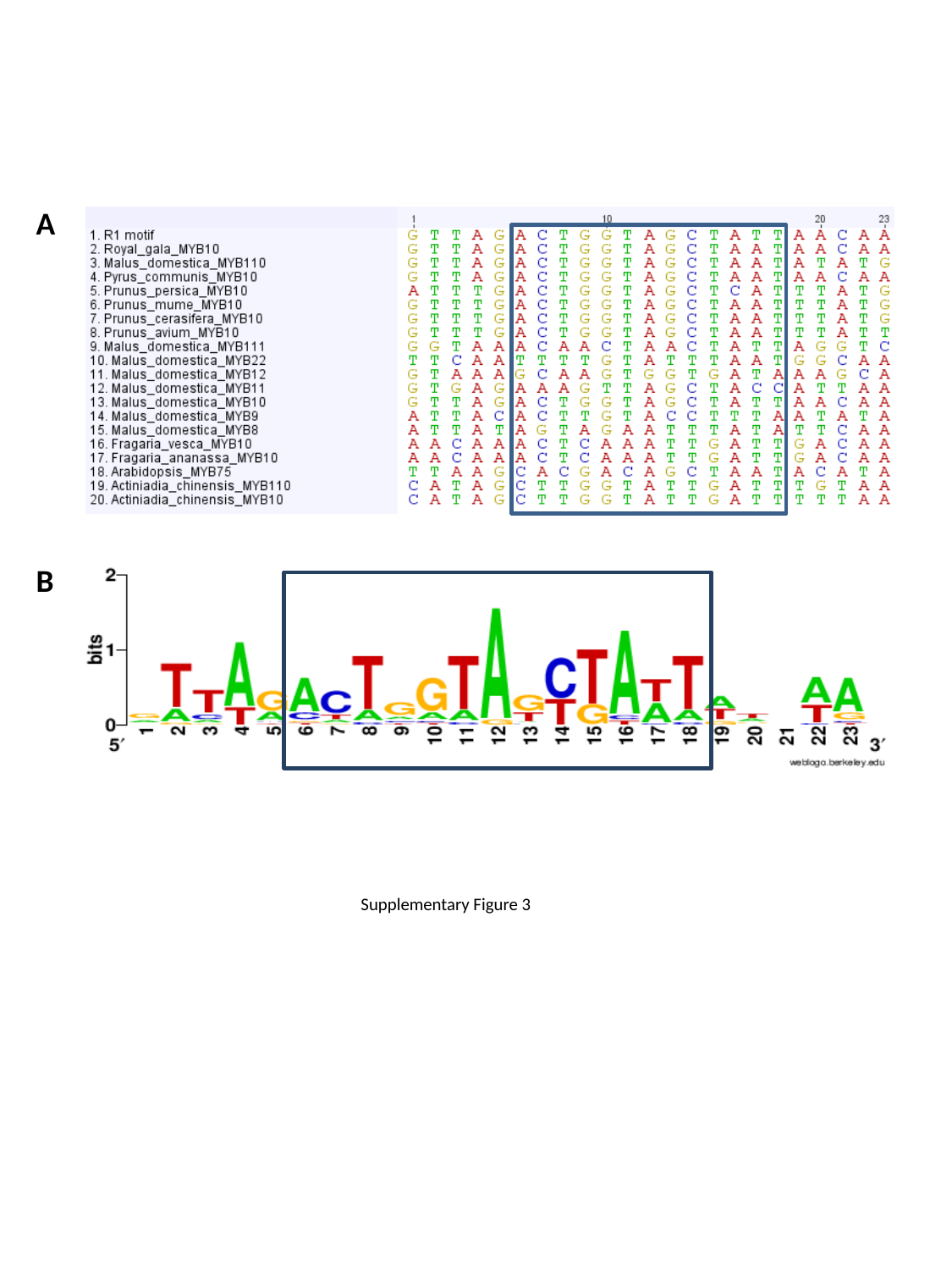

A
B
Supplementary Figure 3
