# Supplementary material for: Multiple Copies of a Simple MYB-Binding Site Confers Trans-regulation by Specific Flavonoid-Related R2R3 MYBs in Diverse Species
Source: Front Plant Sci. 2017 Oct 31;8:1864. doi: 10.3389/fpls.2017.01864 (PMC5671642; doi:10.3389/fpls.2017.01864)
Supplement: Supplementary file 2 [file Table_1.DOCX]

**Supplementary Table 1.** Primers used in this study.

| Name | Sequence |
| --- | --- |
| FW01 | CTGCAGAAATGTTAGACTGGTAGCTATTAAC |
| RV01 | CCAGTGACGTGCATGTCTGATATCC |
| FW02 | GAGCTCATCCCAAGTACGTGTGCAAG |
| RV02 | CTGGAGTATGAAGTGGGTAGCAGGCGGATCCT |
| RV03 | ATGCGGCCGCGTGGATTGATGTTCCTGGCTC |
| FW03 | CACCGGATCCTTTATAGCACAGTTCGGCG |
| RV04 | CTCAACCAAGACAAGCGAAGCC |
| FW04 | CACCTAACCCCCAATCATGTGCTTATATATTG |
| RV05 | GCAATTTTGTTTTGCAATAAATCCATATTTTC |
| RV06 | TTCCATGGTTTGTGTAGTGTATTGCTTTGTGAGTAC |
| OE3 | TGGTACCTCCTCTTTTGAATTGACGTGACAC |
| OE4 | GAACCCTCTTGATCTTCAACATCTCGAACTCAGAAAACGCAAAAAAC |
| OE1 | GTTTTTTGCGTTTTCTGAGTTCGAGATGTTGAAGATCAAGAGGGTTC |
| OE2 | AGGTACCGGCCGCAGATTTAGGTGACAC |
| M1 | CATCTTGGCTATATTCGGAGTTTCTCGTGCCCTC |
| M2 | CCGAATATAGCCAAGATGTCTACAACTTGTCAAACACCGGG |
| qPCR primers |  |
| AtMYB75 FW | TTCCTGTAAGAGCTGGGCTA |
| AtMYB75 RV | TTAAAGACCACCTATTCCCT |
| AtACTIN FW | CTCTCCCGCTATGTATGTCGCCA |
| AtACTIN RV | GTGAGACACACCATCACCAG |
| PcMYB10 FW | ACAAACGTCGTCGTCAACAAAGAAC |
| PcMYB10 RV | TCAATGCTGGGACATGCAGCC |
| PcACTIN FW | TGAGACATTCAACACCCCGGCTAT |
| PcACTIN RV | GATGGCATGTGGGAGGGCATA |
