# Supplementary material for: Multiple Copies of a Simple MYB-Binding Site Confers Trans-regulation by Specific Flavonoid-Related R2R3 MYBs in Diverse Species
Source: Front Plant Sci. 2017 Oct 31;8:1864. doi: 10.3389/fpls.2017.01864 (PMC5671642; doi:10.3389/fpls.2017.01864)
Supplement: Supplementary file 3 [file Data_Sheet_1.docx]

**Supplementary Data Sheet 1**: deduced amino acid sequences of Arabidopsis and apple MYB genes used to generate the phylogenetic tree.

>At3g60460____DUO1

MEAKKEEIKKGPWKAEEDEVLINHVKRYGPRDWSSIRSKGLLQRTGKSCRLRWVNKLRPNLKNGCKFSADEERTVIELQSEFGNKWARIATYLPGRTDNDVKNFWSSRQKRLARILHNSSDASSSSFNPKSSSSHRLKGKNVKPIRQSSQGFGLVEEEVTVSSSCSQMVPYSSDQVGDEVLRLPDLGVKLEHQPFAFGTDLVLAEYSDSQNDANQQAISPFSPESRELLARLDDPFYYDILGPADSSEPLFALPQPFFEPSPVPRRCRHVSKDEEADVFLDDFPADMFDQVDPIPSP

>AtGL1_________AT3G27920_AtGL1

MRIRRRDEKENQEYKKGLWTVEEDNILMDYVLNHGTGQWNRIVRKTGLKRCGKSCRLRWMNYLSPNVNKGNFTEQEEDLIIRLHKLLGNRWSLIAKRVPGRTDNQVKNYWNTHLSKKLVGDYSSAVKTTGEDDDSPPSLFITAATPSSRHHQQENIYENIAKSFNGVVSASYEDKPKQELAQKDVLMATTNDPSHYYGNNALWVHDDDFELSSLVMMNFASSDIEYCL

>AtMYB1_____At3g09230

MEAEIVRRSEVTGLRREVEESSIGRGDCDGDGGDVGEDAAGFVGTSGRGRRDRVKGPWSKEEDDVLSELVKRLGARNWSFIARSIPGRSGKSCRLRWCNQLNPNLIRNSFTEVEDQAIIAAHAIHGNKWAVIAKLLPGRTDNAIKNHWNSALRRRFIDFEKAKNIGTGSLVVDDSGFDRTTTVASSEETLSSGGGCHVTTPIVSPEGKEATTSMEMSEEQCVEKTNGEGISRQDDKDPPTLFRPVPRLSSFNACNHMEGSPSPHIQDQNQLQSSKQDAAMLRLLEGAYSERFVPQTCGGGCCSNNPDGSFQQESLLGPEFVDYLDSPTFPSSELAAIATEIGSLAWLRSGLESSSVRVMEDAVGRLRPQGSRGHRDHYLVSEQGTNITNVLST

>AtMYB2____At2g47190

MEDYERINSNSPTHEEDSDVRKGPWTEEEDAILVNFVSIHGDARWNHIARSSGLKRTGKSCRLRWLNYLRPDVRRGNITLEEQFMILKLHSLWGNRWSKIAQYLPGRTDNEIKNYWRTRVQKQAKHLRCDVNSNLFKETMRNVWMPRLVERINAQSLPTTCEQVESMITDPSQPVNEPSPVEPGFVQFSQNHHQQFVPATELSATSSNSPAETFSDVRGGVVNGSGYDPSGQTGFGEFNDWGCVGGDNMWTDEESFWFLQDQFCPDTTSYSYN

>AtMYB3____At1g22640

MGRSPCCEKAHMNKGAWTKEEDQLLVDYIRKHGEGCWRSLPRAAGLQRCGKSCRLRWMNYLRPDLKRGNFTEEEDELIIKLHSLLGNKWSLIAGRLPGRTDNEIKNYWNTHIKRKLLSRGIDPNSHRLINESVVSPSSLQNDVVETIHLDFSGPVKPEPVREEIGMVNNCESSGTTSEKDYGNEEDWVLNLELSVGPSYRYESTRKVSVVDSAESTRRWGSELFGAHESDAVCLCCRIGLFRNESCRNCRVSDVRTH

>AtMYB3R1

MKREMKAPTTPLESLQGDLKGKQGRTSGPARRSTKGQWTPEEDEVLCKAVERFQGKNWKKIAECFKDRTDVQCLHRWQKVLNPELVKGPWSKEEDNTIIDLVEKYGPKKWSTISQHLPGRIGKQCRERWHNHLNPGINKNAWTQEEELTLIRAHQIYGNKWAELMKFLPGRSDNSIKNHWNSSVKKKLDSYYASGLLDQCQSSPLIALQNKSIASSSSWMHSNGDEGSSRPGVDAEESECSQASTVFSQSTNDLQDEVQRGNEEYYMPEFHSGTEQQISNAASHAEPYYPSFKDVKIVVPEISCETECSKKFQNLNCSHELRTTTATEDQLPGVSNDAKQDRGLELLTHNMDNGGKNQALQQDFQSSVRLSDQPFLSNSDTDPEAQTLITDEECCRVLFPDNMKDSSTSSGEQGRNMVDPQNGKGSLCSQAAETHAHETGKVPALPWHPSSSEGLAGHNCVPLLDSDLKDSLLPRNDSNAPIQGCRLFGATELECKTDTNDGFIDTYGHVTSHGNDDNGGFPEQQGLSYIPKDSLKLVPLNSFSSPSRVNKIYFPIDDKPAEKDKGALCYEPPRFPSADIPFFSCDLVPSNSDLRQEYSPFGIRQLMISSMNCTTPLRLWDSPCHDRSPDVMLNDTAKSFSGAPSILKKRHRDLLSPVLDRRKDKKLKRAATSSLANDFSRLDVMLDEGDDCMTSRPSESPEDKNICASPSIARDNRNCASARLYQEMIPIDEEPKETLESGGVTSMQNENGCNDGGASAKNVSPSLSLHIIWYQL

>AtMYB3R2

MSCPSYINVSLKPFFHLMQDQILTNVVKKYQGRNWKRIAECLPGSEENRRNDVQCQHRWLKVLDPSLQKGAWKKEEDELLSELVKDYMENDRPPWSKISKELPGRIGKQCRERWHNHLNPTIIKSPWTREEELILVQAQRGNGNKWAEIAKLLPGRTENNIKNHWNCSVKKRLEQFPSNLFSGVVYGSKPSSGFEYNFFNQRNTMVESCITSQIKEAAKSPQRDFLDLTLGLNWRSISSSTSSLRGEESVSSSVDSVCARLNACLETPQNSNNDTVCVKEVREMKERLRMAARTFDTPSIISKTSSPASGLKRLRQKYDTPFPTDARSHMSSEEDHSVSASPSSKYRFVKRNTCSGSKPLERRLDFDFLLWDEHGRRNGIVNFSVRILPQKSDLKSGLVRPFWLR

>AtMYB3R3_____AT3g09370

MMDLQEETGEVKIEDQCVENKQSTPASCSSVSEGSAGSSHKSPTIASPATVSPTHRYLGRTSGPIRRAKGGWTPEEDETLRQAVDTFKGKSWKNIAKSFPDRTEVQCLHRWQKVLNPDLIKGPWTHEEDEKIVELVEKYGPAKWSIIAQSLPGRIGKQCRERWHNHLNPDINKDAWTTEEEVALMNAHRSHGNKWAEIAKVLPGRTDNAIKNHWNSSLKKKSEFYLLTGRLPPPTTTRNGVPDSVTKRSSSAQKRVFGSVAQTSSVTTDVNNLAEDGNGQINSSVPVEEVVAASRMTSLNEYARSPQLPNPEPLPENGGAANNGYHLYYTPQIDYYRASEVDTQRMYGNECGCSPSASPVSFFTPPPCRNVHSNGSTPRSPESYLREAGRTYPNTPSIFRKRRPRVVVQDNNNAKKTDEAKEVDQKVNDGKDSSEIQNNGSNAYNLSPPYRIRSKRTAVFKSRQLEFISREEEKADDETKSSEKDMLIDGDSQLLG

>AtMYB3R4______AT5g11510

MEAESSTPQERIPKLRHGRTSGPARRSTRGQWTAEEDEILRKAVHSFKGKNWKKIAEYFKDRTDVQCLHRWQKVLNPELVKGPWTKEEDEMIVQLIEKYGPKKWSTIARFLPGRIGKQCRERWHNHLNPAINKEAWTQEEELLLIRAHQIYGNRWAELTKFLPGRSDNGIKNHWHSSVKKKLDSYMSSGLLDQYQAMPLAPYERSSTLQSTFMQSNIDGNGCLNGQAENEIDSRQNSSMVGCSLSARDFQNGTINIGHDFHPCGNSQENEQTAYHSEQFYYPELEDISVSISEVSYDMEDCSQFPDHNVSTSPSQDYQFDFQELSDISLEMRHNMSEIPMPYTKESKESTLGAPNSTLNIDVATYTNSANVLTPETECCRVLFPDQESEGHSVSRSLTQEPNEFNQVDRRDPILYSSASDRQISEATKSPTQSSSSRFTATAASGKGTLRPAPLIISPDKYSKKSSGLICHPFEVEPKCTTNGNGSFICIGDPSSSTCVDEGTNNSSEEDQSYHVNDPKKLVPVNDFASLAEDRPHSLPKHEPNMTNEQHHEDMGASSSLGFPSFDLPVFNCDLLQSKNDPLHDYSPLGIRKLLMSTMTCMSPLRLWESPTGKKTLVGAQSILRKRTRDLLTPLSEKRSDKKLEIDIAASLAKDFSRLDVMFDETENRQSNFGNSTGVIHGDRENHFHILNGDGEEWSGKPSSLFSHRMPEETMHIRKSLEKVDQICMEANVREKDDSEQDVENVEFFSGILSEHNTGKPVLSTPGQSVTKAEKAQVSTPRNQLQRTLMATSNKEHHSPSSVCLVINSPSRARNKEGHLVDNGTSNENFSIFCGTPFRRGLESPSAWKSPFYINSLLPSPRFDTDLTIEDMGYIFSPGERSYESIGVMTQINEHTSAFAAFADAMEVSISPTNDDARQKKELDKENNDPLLCWTSTIASLQSKQQKKSHPTS

>AtMYB3R5_____At5g02320

MKIEIQCMENKQPLAASCSSASEGSGCFFLKSPEIATPATVSSFPRRTSGPMRRAKGGWTPEEDETLRRAVEKYKGKRWKKIAEFFPERTEVQCLHRWQKVLNPELVKGPWTQEEDDKIVELVKKYGPAKWSVIAKSLPGRIGKQCRERWHNHLNPGIRKDAWTVEEESALMNSHRMYGNKWAEIAKVLPGRTDNAIKNHWNSSLKKKLEFYLATGNLPPPASKFIVLKDIADGDRDSKQSSATKPFKDSDSLTQTSSGNTDSNEVGRDHFDSSSALLEEVAASRRIGVNEYACSPVEYKPQLPNLEPISEEVRINSKAYFERSIQRKVENGFGTPKHGNLYYKSPLDYYFPSEADLQHMYGYECGCSPGAASPVSLMTTPCNKDSGLTATRSPESFLREAARTFPNTPSIFRKRRKVVLAAKTDAVVVVNGVVKEVDRKEESKDMRKSLLLETTDNCSDDEELGLNGNAFNLSPPYRLRAKRTAVIKSRQLEFTSEKEKQPDNEIEFTSAKEKQPDNEIKTSEEDKPV

>AtMYB4____86_At4g38620

MGRSPCCEKAHTNKGAWTKEEDERLVAYIKAHGEGCWRSLPKAAGLLRCGKSCRLRWINYLRPDLKRGNFTEEEDELIIKLHSLLGNKWSLIAGRLPGRTDNEIKNYWNTHIRRKLINRGIDPTSHRPIQESSASQDSKPTQLEPVTSNTINISFTSAPKVETFHESISFPGKSEKISMLTFKEEKDECPVQEKFPDLNLELRISLPDDVDRLQGHGKSTTPRCFKCSLGMINGMECRCGRMRCDVVGGSSKGSDMSNGFDFLGLAKKETTSLLGFRSLEMK

>AtMYB5_____At3g13540

MMSCGGKKPVSKKTTPCCTKMGMKRGPWTVEEDEILVSFIKKEGEGRWRSLPKRAGLLRCGKSCRLRWMNYLRPSVKRGGITSDEEDLILRLHRLLGNRWSLIAGRIPGRTDNEIKNYWNTHLRKKLLRQGIDPQTHKPLDANNIHKPEEEVSGGQKYPLEPISSSHTDDTTVNGGDGDSKNSINVFGGEHGYEDFGFCYDDKFSSFLNSLINDVGDPFGNIIPISQPLQMDDCKDGIVGASSSSLGHD

>AtMYB6____At4g09460

MGRSPCCEKAHTNKGAWTKEEDQRLVDYIRNHGEGCWRSLPKSAGLLRCGKSCRLRWINYLRPDLKRGNFTDDEDQIIIKLHSLLGNKWSLIAGRLPGRTDNEIKNYWNTHIKRKLLSHGIDPQTHRQINESKTVSSQVVVPIQNDAVEYSFSNLAVKPKTENSSDNGASTSGTTTDEDLRQNGECYYSDNSGHIKLNLDLTLGFGSWSGRIVGVGSSADSKPWCDPVMEARLSLL

>AtMYB7____At2g16720

MGRSPCCEKEHMNKGAWTKEEDERLVSYIKSHGEGCWRSLPRAAGLLRCGKSCRLRWINYLRPDLKRGNFTHDEDELIIKLHSLLGNKWSLIAARLPGRTDNEIKNYWNTHIKRKLLSKGIDPATHRGINEAKISDLKKTKDQIVKDVSFVTKFEETDKSGDQKQNKYIRNGLVCKEERVVVEEKIGPDLNLELRISPPWQNQREISTCTASRFYMENDMECSSETVKCQTENSSSISYSSIDISSSNVGYDFLGLKTRILDFRSLEMK

>AtMYB8____At1g35515_HOS10

MGRSPCCEKAHMNKGAWTKEEDQRLIDYIRNHGEGSWRSLPKSVGKFLQRLLRCGKSCRLRWINYLRPDLKRGNFTDGEEQIIVKLHSLFGNKEKASDLLFPATIASLVFVGQFSVQNLMAFPAWMSGFCPAMFRATFDCRGSSDTVRFFTTMVGLRLASIGFFSGGRLAKTSAFLFFFKVLLKAPSAYSTSMFFRFQESIAFGRSFLSKFANENGGEWRQLRLLQSTWSLIAGKLPGRTDNEIKNYWNTHIKRKLLNRGIDPKTHGSIIEPKTTSFHPRNEDLKSTFPGSVKLKMETSCENCASTSGTTTDEDLRLSVDCDYRYDHLDKELNLDLTLGYSPTRFVGVGSCY

>AtMYB9____At5g16770

MGRSPCCDENGLKKGPWTQEEDDKLIDHIQKHGHGSWRALPKQAGLNRCGKSCRLRWTNYLRPDIKRGNFTEEEEQTIINLHSLLGNKWSSIAGNLPGRTDNEIKNYWNTHLRKKLLQMGIDPVTHRPRTDHLNVLAALPQLIAAANFNSLLNLNQNVQLDATTLAKAQLLHTMIQVLSTNNNTTNPSFSSSTMQNSNTNLFGQASYLENQNLFGQSQNFSHILEDENLMVKTQIIDNPLDSFSSPIQPGFQDDHNSLPLLVPASPEESKETQRMIKNKDIVDYHHHDASNPSSSNSTFTQDHHHPWCDTIDDGASDSFWKEIIE

>AtMYB10_____At3g12820

MGNRRAPCCDKSQVKRGPWSDEESERLRSFILKNGHQNWRSLPKLAGLMRCGKSCRLRWINYLRPGLKRGNFTKEEEDTIIHLHQAYGNKWSKIASNFPGRTDNEIKNVWNTHLKKRLVKRSISSSSSDVTNHSVSSTSSSSSSISSVLQDVIIKSERPNQEEEFGEILVEQMACGFEVDAPQSLECLFDDSQVPPPISKPDSLQTHGKSSDHEFWSRLIEPGFDDYNEWLIFLDNQTC

>AtMYB11______At3g62610

MGRAPCCEKVGIKKGRWTAEEDRTLSDYIQSNGEGSWRSLPKNAGLKRCGKSCRLRWINYLRSDIKRGNITPEEEDVIVKLHSTLGTRWSTIASNLPGRTDNEIKNYWNSHLSRKLHGYFRKPTVANTVENAPPPPKRRPGRTSRSAMKPKFILNPKNHKTPNSFKANKSDIVLPTTTIENGEGDKEDALMVLSSSSLSGAEEPGLGPCGYGDDGDCNPSINGDDGALCLNDDIFDSCFLLDDSHAVHVSSCESNNVKNSEPYGGMSVGHKNIETMADDFVDWDFVWREGQTLWDEKEDLDSVLSRLLDGEEMESEIRQRDSNDFGEPLDIDEENKMAAWLLS

>AtMYB12_____At2g47460

MGRAPCCEKVGIKRGRWTAEEDQILSNYIQSNGEGSWRSLPKNAGLKRCGKSCRLRWINYLRSDLKRGNITPEEEELVVKLHSTLGNRWSLIAGHLPGRTDNEIKNYWNSHLSRKLHNFIRKPSISQDVSAVIMTNASSAPPPPQAKRRLGRTSRSAMKPKIHRTKTRKTKKTSAPPEPNADVAGADKEALMVESSGAEAELGRPCDYYGDDCNKNLMSINGDNGVLTFDDDIIDLLLDESDPGHLYTNTTCGGDGELHNIRDSEGARGFSDTWNQGNLDCLLQSCPSVESFLNYDHQVNDASTDEFIDWDCVWQEGSDNNLWHEKENPDSMVSWLLDGDDEATIGNSNCENFGEPLDHDDESALVAWLLS

>AtMYB13_____AT1G06180

MGRRPCCEKIGLKKGPWSAEEDRILINYISLHGHPNWRALPKLAGLLRCGKSCRLRWINYLRPDIKRGNFTPHEEDTIISLHQLLGNRWSAIAAKLPGRTDNEIKNVWHTHLKKRLHHSQDQNNKEDFVSTTAAEMPTSPQQQSSSSADISAITTLGNNNDISNSNKDSATSSEDVLAIIDESFWSEVVLMDCDISGNEKNEKKIENWEGSLDRNDKGYNHDMEFWFDHLTSSSCIIGEMSDISEF

>AtMYB14_____At2g31180

MGRAPCCEKMGVKRGPWTPEEDQILINYIHLYGHSNWRALPKHAGLLRCGKSCRLRWINYLRPDIKRGNFTPQEEQTIINLHESLGNRWSAIAAKLPGRTDNEIKNVWHTHLKKRLSKNLNNGGDTKDVNGINETTNEDKGSVIVDTASLQQFSNSITTFDISNDNKDDIMSYEDISALIDDSFWSDVISVDNSNKNEKKIEDWEGLIDRNSKKCSYSNSKLYNDDMEFWFDVFTSNRRIEEFSDIPEF

>AtMYB15_____At3g23250

MGRAPCCEKMGLKRGPWTPEEDQILVSFILNHGHSNWRALPKQAGLLRCGKSCRLRWMNYLKPDIKRGNFTKEEEDAIISLHQILGNRWSAIAAKLPGRTDNEIKNVWHTHLKKRLEDYQPAKPKTSNKKKGTKPKSESVITSSNSTRSESELADSSNPSGESLFSTSPSTSEVSSMTLISHDGYSNEINMDNKPGDISTIDQECVSFETFGADIDESFWKETLYSQDEHNYVSNDLEVAGLVEIQQEFQNLGSANNEMIFDSEMDFWFDVLARTGGEQDLLAGL

>AtMYB16____ATMIXTA

MGRSPCCDKLGLKKGPWTPEEDQKLLAYIEEHGHGSWRSLPEKAGLHRCGKSCRLRWTNYLRPDIKRGKFNLQEEQTIIQLHALLGNRWSAIATHLPKRTDNEIKNYWNTHLKKRLVKMGIDPVTHKPKNETPLSSLGLSKNAAILSHTAQWESARLEAEARLARESKLLHLQHYQTKTSSQPHHHHGFTHKSLLPNWTTKPHEDQQQLESPTSTVSFSEMKESIPAKIEFVGSSTGVTLMKEPEHDWINSTMHEFETTQMGEGIEEGFTGLLLGGDSIDRSFSGDKNETAGESSGGDCNYYEDNKNYLDSIFNFVDPSPSDSPMF

>AtMYB17

MGRTPCCDKIGLKKGPWTPEEDEVLVAHIKKNGHGSWRTLPKLAGLLRCGKSCRLRWTNYLRPDIKRGPFTADEEKLVIQLHAILGNRWAAIAAQLPGRTDNEIKNLWNTHLKKRLLSMGLDPRTHEPLPSYGLAKQAPSSPTTRHMAQWESARVEAEARLSRESMLFSPSFYSGVVKTECDHFLRIWNSEIGEAFRNLAPLDESTITSQSPCSRATSTSSALLKSSTNSWGGKEVTVAIHGSDYSPYSNDLEDDSTDSALQLLLDFPISDDDMSFLEENIDSYSQAPPIGLVSMVSKF

>AtMYB18____At4g25560_LAF1

MAKTKYGERHRKGLWSPEEDEKLRSFILSYGHSCWTTVPIKAGLQRNGKSCRLRWINYLRPGLKRDMISAEEEETILTFHSSLGNKWSQIAKFLPGRTDNEIKNYWHSHLKKKWLKSQSLQDAKSISPPSSSSSSLVACGKRNPETLISNHVFSFQRLLENKSSSPSQESNGNNSHQCSSAPEIPRLFFSEWLSSSYPHTDYSSEFTDSKHSQAPNVEETLSAYEEMGDVDQFHYNEMMINNSNWTLNDIVFGSKCKKQEHHIYREASDCNSSAEFFSPSTTT

>AtMYB19

MTKSGERPKQRQRKGLWSPEEDQKLKSFILSRGHACWTTVPILAGLQRNGKSCRLRWINYLRPGLKRGSFSEEEEETILTLHSSLGNKWSRIAKYLPGRTDNEIKNYWHSYLKKRWLKSQPQLKSQISDLTESPSSLLSCGKRNLETETLDHVISFQKFSENPTSSPSKESNNNMIMNNSNNLPKLFFSEWISSSNPHIDYSSAFTDSKHINETQDQINEEEVMMINNNNYSSLEDVMLRTDFLQPDHEYANYYSSGDFFINSDQNYV

>AtMYB20____At1g66230

MGRQPCCDKVGLKKGPWTAEEDRKLINFILTNGQCCWRAVPKLSGLLRCGKSCRLRWTNYLRPDLKRGLLSDYEEKMVIDLHSQLGNRWSKIASHLPGRTDNEIKNHWNTHIKKKLRKMGIDPLTHKPLSIVEKEDEEPLKKLQNNTVPFQETMERPLENNIKNISRLEESLGDDQFMEINLEYGVEDVPLIETESLDLICSNSTMSSSTSTSSHSSNDSSFLKDLQFPEFEWSDYGNSNNDNNNGVDNIIENNMMSLWEISDFSSLDLLLNDESSSTFGLF

>AtMYB21_____At3g27810

MEKRGGGSSGGSGSSAEAEVRKGPWTMEEDLILINYIANHGDGVWNSLAKSAGLKRTGKSCRLRWLNYLRPDVRRGNITPEEQLIIMELHAKWGNRWSKIAKHLPGRTDNEIKNFWRTRIQKYIKQSDVTTTSSVGSHHSSEINDQAASTSSHNVFCTQDQAMETYSPTPTSYQHTNMEFNYGNYSAAAVTATVDYPVPMTVDDQTGENYWGMDDIWSSMHLLNGN

>AtMYB22_____At5g40430

MEFSLISDENFILNDAHRCPVNDDASHIPKNNLNFFNDNLGQSSRTGWSFSPDLTDISNQHHQNLIPLIPNYDSQNQNLDTNQNHLVYNSSSYEIPSNYPFMSIKSYSNIDTLEQSMNNIVNNGKIHMIDNPPIFANPKGIFENFHDLQEYTIGNEIVHNEELTNKGYEPTLDKVMGEPQLFDVPVLEGIKNTTNEIMNQLEDDKMKKTYENKKEASTSKYLKKSDITKKRWTESEDIKLKEMVALEPKKWTKVAKHFEGRTPKQCRERWHNHARPNVKKTTWSEEEDQILIEVHKVIGAKWIQISEQLPGRSYNNVKNHWNTTKRRVQNKSGRTVNRVGNNILENYIRSITINNDDESDGEPTNIENYHDDSEDMLYGEMNLSPEAITQTTKPLTDASTISPYIPMPKENYTLEVCESLEDYLELLRWWD

>AtMYB23_____At5g40330

MRMTRDGKEHEYKKGLWTVEEDKILMDYVRTHGQGHWNRIAKKTGLKRCGKSCRLRWMNYLSPNVNRGNFTDQEEDLIIRLHKLLGNRWSLIAKRVPGRTDNQVKNYWNTHLSKKLGLGDHSTAVKAACGVESPPSMALITTTSSSHQEISGGKNSTLRFDTLVDESKLKPKSKLVHATPTDVEVAATVPNLFDTFWVLEDDFELSSLTMMDFTNGYCL

>AtMYB24_____At5g40350

MEKRESSGGSGSGDAEVRKGPWTMEEDLILINYIANHGEGVWNSLAKSAGLKRTGKSCRLRWLNYLRPDVRRGNITPEEQLTIMELHAKWGNRWSKIAKHLPGRTDNEIKNFWRTKIQKYIIKSGETTTVGSQSSEFINHHATTSHVMNDTQETMDMYSPTTSYQHASNINQQLNYGNYVPESGSIMMPLSVDQSEQNYWSVDDLWPMNIYNGN

>AtMYB25_____At2g39880

MNGEISRPPELISSRNPCKSFENAIHKAVEAELAELAKSDANGGGKSKVKGPWLPEQDEALTRLVKMCGPRNWNLISRGIPGRSGKSCRLRWCNQLDPILKRKPFSDEEEHMIMSAQAVLGNKWSVIAKLLPGRTDNAIKNHWNSNLRRKPAEQWKIPLLMSNTEIVYQLYPSMVRRISNASPKEHLPQEEETGVLSDDKMDDEAKEPPREQNSKTGVYRPVARMGAFSVCKPGYMAPCEGPLVQASRPDSLAGKFLQSLCYDPIIPSKCGHGCCNHQDSTTLSSSSVLGSEFVDYEEHSSAELDKELISISNDLNNTAWIRSGKEAEQSLKADDQFRREYAHSKFSGMVNNGVSSQMVRQDLRALS

>AtMYB26____At3g13890

MGHHSCCNKQKVKRGLWSPEEDEKLINYINSYGHGCWSSVPKHAGLQRCGKSCRLRWINYLRPDLKRGSFSPQEAALIIELHSILGNRWAQIAKHLPGRTDNEVKNFWNSSIKKKLMSHHHHGHHHHHLSSMASLLTNLPYHNGFNPTTVDDESSRFMSNIITNTNPNFITPSHLSLPSPHVMTPLMFPTSREGDFKFLTTNNPNQSHHHDNNHYNNLDILSPTPTINNHHQPSLSSCPHDNNLQWPALPDFPASTISGFQETLQDYDDANKLNVFVTPFNDNAKKLLCGEVLEGKVLSSSSPISQDHGLFLPTTYNFQMTSTSDHQHHHRVDSYINHMIIPSSSSSSPISCGQYVIT

>AtMYB27______At3g532006

MDFKKEETLRRGPWLEEEDERLVKVISLLGERRWDSLAIVSGLKRSGKSCRLRWMNYLNPTLKRGPMSQEEERIIFQLHALWGNKWSKIARRLPGRTDNEIKNYWRTHYRKKQEAQNYGKLFEWRGNTGEELLHKYKETEITRTKTTSQEHGFVEVVSMESGKEANGGVGGRESFGVMKSPYENRISDWISEISTDQSEANLSEDHSSNSCSENNINIGTWWFQETRDFEEFSCSLWS

>AtMYB28_____At5g61420

MSRKPCCVGEGLKKGAWTTEEDKKLISYIHDHGEGGWRDIPQKAGLKRCGKSCRLRWTNYLKPEIKRGEFSSEEEQIIIMLHASRGNKWSVIARHLPRRTDNEIKNYWNTHLKKRLMEQGIDPVTHKPLASSSNPTVDENLNSPNASSSDKQYSRSSSMPFLSRPPPSSCNMVSKVSELSSNDGTPIQGSSLSCKKRFKKSSSTSRLLNKVAAKATSIKDILSASMEGSLSATTISHASFFNGFTEQIRNEEDSSNTSLTNTLAEFDPFSPSSLYPEHEINATSDLNMDQDYDFSQFFEKFGGDNHNEENSMNDLLMSDVSQEVSSTSVDDQDNMVGNFEGWSNYLLDHTNFMYDTDSDSLEKHFI

>AtMYB29_____At5g07690

MSRKPCCVGEGLKKGAWTAEEDKKLISYIHEHGEGGWRDIPQKAGLKRCGKSCRLRWANYLKPDIKRGEFSYEEEQIIIMLHASRGNKWSVIARHLPKRTDNEIKNYWNTHLKKLLIDKGIDPVTHKPLAYDSNPDEQSQSGSISPKSLPPSSSKNVPEITSSDETPKYDASLSSKKRCFKRSSSTSKLLNKVAARASSMGTILGASIEGTLISSTPLSSCLNDDFSETSQFQMEEFDPFYQSSEHIIDHMKEDISINNSEYDFSQFLEQFSNNEGEEADNTGGGYNQDLLMSDVSSTSVDEDEMMQNITGWSNYLLDHSDFNYDTSQDYDDKNFI

>AtMYB30_____At3g28910

MVRPPCCDKGGVKKGPWTPEEDIILVTYIQEHGPGNWRAVPTNTGLLRCSKSCRLRWTNYLRPGIKRGNFTEHEEKMIVHLQALLGNRWAAIASYLPQRTDNDIKNYWNTHLKKKLNKVNQDSHQELDRSSLSSSPSSSSANSNSNISRGQWERRLQTDIHLAKKALSEALSPAVAPIITSTVTTTSSSAESRRSTSSASGFLRTQETSTTYASSTENIAKLLKGWVKNSPKTQNSADQIASTEVKEVIKSDDGKECAGAFQSFSEFDHSYQQAGVSPDHETKPDITGCCSNQSQWSLFEKWLFEDSGGQIGDILLDENTNFF

>AtMYB31_____At1g74650

MGRPPCCEKIEVKKGPWTPEEDIILVSYIQQHGPGNWRSVPANTGLLRCSKSCRLRWTNYLRPGIKRGNFTQPEEKMIIHLQALLGNRWAAIASYLPQRTDNDIKNYWNTHLKKKLVMMKFQNGIINENKTNLATDISSCNNNNNGCNHNKRTTNKGQWEKKLQTDINMAKQALFQALSLDQPSSLIPPDPDSPKPHHHSTTTYASSTDNISKLLQNWTSSSSSKPNTSSVSNNRSSSPGEGGLFDHHSLFSSNSESGSVDEKLNLMSETSMFKGESKPDIDMEATPTTTTTDDQGSLSLIEKWLFDDQGLVQCDDSQEDLIDVSLEELK

>AtMYB32____At4g34990

MGRSPCCEKDHTNKGAWTKEEDDKLISYIKAHGEGCWRSLPRSAGLQRCGKSCRLRWINYLRPDLKRGNFTLEEDDLIIKLHSLLGNKWSLIATRLPGRTDNEIKNYWNTHVKRKLLRKGIDPATHRPINETKTSQDSSDSSKTEDPLVKILSFGPQLEKIANFGDERIQKRVEYSVVEERCLDLNLELRISPPWQDKLHDERNLRFGRVKYRCSACRFGFGNGKECSCNNVKCQTEDSSSSSYSSTDISSSIGYDFLGLNNTRVLDFSTLEMK

>AtMYB33____At5g06100

MSYTSTDSDHNESPAADDNGSDCRSRWDGHALKKGPWSSAEDDILIDYVNKHGEGNWNAVQKHTSLFRCGKSCRLRWANHLRPNLKKGAFSQEEEQLIVELHAKMGNRWARMAAHLPGRTDNEIKNYWNTRIKRRQRAGLPLYPPEMHVEALEWSQEYAKSRVMGEDRRHQDFLQLGSCESNVFFDTLNFTDMVPGTFDLADMTAYKNMGNCASSPRYENFMTPTIPSSKRLWESELLYPGCSSTIKQEFSSPEQFRNTSPQTISKTCSFSVPCDVEHPLYGNRHSPVMIPDSHTPTDGIVPYSKPLYGAVKLELPSFQYSETTFDQWKKSSSPPHSDLLDPFDTYIQSPPPPTGGEESDLYSNFDTGLLDMLLLEAKIRNNSTKNNLYRSCASTIPSADLGQVTVSQTKSEEFDNSLKSFLVHSEMSTQNADETPPSELLFTLPSLEIVC

>AtMYB34_____At5g60890

MVRTPCCKEEGIKKGAWTPEEDQKLIAYLHLHGEGGWRTLPEKAGLKRCGKSCRLRWANYLRPDIKRGEFSPEEDDTIIKLHALKGNKWAAIATSLAGRTDNEIKNYWNTNLKKRLKQKGIDAITHKPINSTGQTGFEPKVNKPVYSSGSARLLNRVASKYAVELNRDLLTGIISGNSTVAEDSQNSGDVDSPTSTLLNKMAATSVLINTTTTYSGFSDNCSFTDEFNEFFNNEEISDIYTTVDNFGFMEELKSILSYGDASAGVIENSPEVNVADAMEFIDSWNEDDNMVGVFV

>AtMYB35

MGRPPCCDKSNVKKGLWTEEEDAKILAYVAIHGVGNWSLIPKKAGLNRCGKSCRLRWTNYLRPDLKHDSFSTQEEELIIECHRAIGSRWSSIARKLPGRTDNDVKNHWNTKLKKKLMKMGIDPVTHKPVSQLLAEFRNISGHGNASFKTEPSNNSILTQSNSAWEMMRNTTTNHESYYTNSPMMFTNSSEYQTTPFHFYSHPNHLLNGTTSSCSSSSSSTSITQPNQVPQTPVTNFYWSDFLLSDPVPQVVGSSATSDLTFTQNEHHFNIEAEYISQNIDSKASGTCHSASSFVDEILDKDQEMLSQFPQLLNDFDY

>AtMYB36_____At5g57620

MGRAPCCDKANVKKGPWSPEEDVKLKDYIDKYGTGGNWIALPQKIGLKRCGKSCRLRWLNYLRPNIKHGGFSEEEDRIILSLYISIGSRWSIIAAQLPGRTDNDIKNYWNTKLKKKLLGRQKQMNRQDSITDSTENNLSNNNNNKSPQNLSNSALERLQLHMQLQNLQSPFSSFYNNPILWPKLHPLLQSTTTNQNPKLASQESFHPLGVNVDHQHNNTKLAQINNGASSLYSENVEQSQNPAHEFQPNFGFSQDLRLDNHNMDFMNRGVSKELFQVGNEFELTNGSSWWSEEVELERKTTSSSSWGSASVLDQTTEGMVMLQDYAQMSYHSV

>AtMYB37

MGRAPCCDKTKVKRGPWSPEEDSKLRDYIEKYGNGGNWISFPLKAGLRRCGKSCRLRWLNYLRPNIKHGDFSEEEDRIIFSLFAAIGSRWSIIAAHLPGRTDNDIKNYWNTKLRKKLLSSSSDSSSSAMASPYLNPISQDVKRPTSPTTIPSSSYNPYAENPNQYPTKSLISSINGFEAGDKQIISYINPNYPQDLYLSDSNNNTSNANGFLLNHNMCDQYKNHTSFSSDVNGIRSEIMMKQEEIMMMMMIDHHIDQRTKGYNGEFTQGYYNYYNGHGDLKQMISGTGTNSNINMGGSGSSSSSISNLAENKSSGSLLLEYKCLPYFYS

>AtMYB38____At2g36890_RAX2

MGRAPCCDKANVKRGPWSPEEDAKLKDYIEKQGTGGNWIALPHKAGLRRCGKSCRLRWLNYLRPNIRHGDFTEEEDNIIYSLFASIGSRWSVIAAHLQGRTDNDIKNYWNTKLKKKLIATMAPPPHHHLAIATSSSSASPSSSSHYNMINSLLPYNPSTNQLLTPHQGIMMTMMGQQQQLFYQEDMGNLVNSPNRNNLIMSHQEDNQEQSTNKGIMLLSDVRSGSSTTSTVTRVKMEHRDHDDHHHHHEEDERSMTSVVMEDYGMEEIKQLISSSCTSSNNSLWFDENKTEDKFMLYY

>AtMYB39

MGRSPCCDQDKGVKKGPWLPEEDDKLTAYINENGYGNWRSLPKLAGLNRCGKSCRLRWMNYLRPDIRRGKFSDGEESTIVRLHALLGNKWSKIAGHLPGRTDNEIKNYWNTHMRKKLLQMGIDPVTHEPRTNDLSPILDVSQMLAAAINNGQFGNNNLLNNNTALEDILKLQLIHKMLQIITPKAIPNISSFKTNLLNPKPEPVVNSFNTNSVNPKPDPPAGLFINQSGITPEAASDFIPSYENVWDGFEDNQLPGLVTVSQESLNTAKPGTSTTTKVNDHIRTGMMPCYYGDQLLETPSTGSVSVSPETTSLNHPSTAQHSSGSDFLEDWEKFLDDETSDSCWKSFLEKIDKKSKNKQSPRMEEEEKNPSSIYIVADLLEDIFLRLPLKSILISKSVSKRWRSILESKTFVERRMSLQKKRKILAAYNCKCGWEPRLLPGSSQCKGNEEIVYLHCNAAQPSFTCDGLVCILEPRWIDVLNPWTRQLRRYGFGFGTIFGVDKVTGSYKVVKMCLISFSEICARDPEVEYSVLDVETGEWRMLSPPPYKVFEVRKSECANGSIYWLHKPTERAWTILALDLHKEELHNISVPDMSVTQETFQIVNLEDRLAIANTYTKTEWKLEIWSMDTEVETWTKTYSIDLENRVASRERRNRWFTPVSVSKQGNIVFYDNHKRLFKYYPRKNEILYLSADTCVISPFFENLAPLPQKSTLHTPIIAGEPNAPLTTLVVVGSIWSPLFLFSVKL

>AtMYB40____At5g14340

MGRKPCCDKIGLKRGPWTIEEDHRLMNFILNNGIHCWRIVPKLAGLLRCGKSCRLRWINYLRPDLKRGGFTDAEEDRIMELHSQLGNRWSKIASHFSGRTDNEIKNHWNTKIKKKMKHLGLDPATHKPMNDITHQTDPNQDKKPNMCSTINEGEEIKDQTPKDDVITETTKTLMLSDNDEELVAKNCKILCAEEVDLESLFETQCNEISSSSFSSLCSNISRSESSSYLAEDSISLEQWDLDMTDPFVPWDLFANLDDNLFLL

>AtMYB41____At4g28110

MGRSPCCDKNGVKKGPWTAEEDQKLIDYIRFHGPGNWRTLPKNAGLHRCGKSCRLRWTNYLRPDIKRGRFSFEEEETIIQLHSVMGNKWSAIAARLPGRTDNEIKNHWNTHIRKRLVRSGIDPVTHSPRLDLLDLSSLLSALFNQPNFSAVATHASSLLNPDVLRLASLLLPLQNPNPVYPSNLDQNLQTPNTSSESSQPQAETSTVPTNYETSSLEPMNARLDDVGLADVLPPLSESFDLDSLMSTPMSSPRQNSIEAETNSSTFFDFGIPEDFILDDFMF

>AtMYB42____At4g12350

MMALPKLAGLRRCGKSCRLRWTNYLRPDLKRGLLSDAEEQLVIDLHALLGNRWSKIAARLPGRTDNEIKNHWNTHIKKKLLKMEIDPSTHQPLNKVFTDTNLVDKSETSSKADNVNDNKIVEIDGTTTNTIDDSIITHQNSSNDDYELLGDIIHNYGDLFNILWTNDEPPLVDDASWSNHNVGIGGTAAVAASDKNNTAAEEDFPERSFEKQNGESWMFLDYCQEFGVEDFGFECYHGFGQSSMKTGHKD

>AtMYB43____At5g16600

MGRQPCCDKVGLKKGPWTIEEDKKLINFILTNGHCCWRALPKLSGLLRCGKSCRLRWINYLRPDLKRGLLSEYEEQKVINLHAQLGNRWSKIASHLPGRTDNEIKNHWNTHIKKKLRKMGIDPLTHKPLSEQEASQQAQGRKKSLVPHDDKNPKQDQQTKDEQEQHQLEQALEKNNTSVSGDGFCIDEVPLLNPHEILIDISSSHHHHSNDDNVNINTSKFTSPSSSSSSTSSCISSVVPGDEFSKFFDEMEILDLKWLSSDDSLGDDISKDGKFNNSTVDTMNLWDINDLSSLDMFMNEHDDGFIGNGNGCSRMVLDQDSWTFDLL

>AtMYB44______At5g67300

MADRIKGPWSPEEDEQLRRLVVKYGPRNWTVISKSIPGRSGKSCRLRWCNQLSPQVEHRPFSAEEDETIARAHAQFGNKWATIARLLNGRTDNAVKNHWNSTLKRKCGGYDHRGYDGSEDHRPVKRSVSAGSPPVVTGLYMSPGSPTGSDVSDSSTIPILPSVELFKPVPRPGAVVLPLPIETSSSSDDPPTSLSLSLPGADVSEESNRSHESTNINNTTSSRHNHNNTVSFMPFSGGFRGAIEEMGKSFPGNGGEFMAVVQEMIKAEVRSYMTEMQRNNGGGFVGGFIDNGMIPMSQIGVGRIE

>AtMYB45

MVFKSEKSNREMKSKEKQRKGLWSPEEDEKLRSHVLKYGHGCWSTIPLQAGLQRNGKSCRLRWVNYLRPGLKKSLFTKQEETILLSLHSMLGNKWSQISKFLPGRTDNEIKNYWHSNLKKGVTLKQHETTKKHQTPLITNSLEALQSSTERSSSSINVGETSNAQTSSFSPNLVFSEWLDHSLLMDQSPQKSSYVQNLVLPEERGFIGPCGPRYLGNDSLPDFVPNSEFLLDDEISSEIEFCTSFSDNFLFDGLINELRPM

>AtMYB46_____At5g12870

MRKPEVAIAASTHQVKKMKKGLWSPEEDSKLMQYMLSNGQGCWSDVAKNAGLQRCGKSCRLRWINYLRPDLKRGAFSPQEEDLIIRFHSILGNRWSQIAARLPGRTDNEIKNFWNSTIKKRLKKMSDTSNLINNSSSSPNTASDSSSNSASSLDIKDIIGSFMSLQEQGFVNPSLTHIQTNNPFPTGNMISHPCNDDFTPYVDGIYGVNAGVQGELYFPPLECEEGDWYNANINNHLDELNTNGSGNAPEGMRPVEEFWDLDQLMNTEVPSFYFNFKQSI

>AtMYB47____At1g18710

MGRTTWFDVDGMKKGEWTAEEDQKLGAYINEHGVCDWRSLPKRAGLQRCGKSCRLRWLNYLKPGIRRGKFTPQEEEEIIQLHAVLGNRWAAMAKKMQNRTDNDIKNHWNSCLKKRLSRKGIDPMTHEPIIKHLTVNTTNADCGNSSTTTSPSTTESSPSSGSSRLLNKLAAGISSRQHSLDRIKYILSNSIIESSDQAKEEEEKEEEEEERDSMMGQKIDGSEGEDIQIWGEEEVRRLMEIDAMDMYEMTSYDAVMYESSHILDHLF

>AtMYB48

MMQEEGNRKGPWTEQEDILLVNFVHLFGDRRWDFIAKVSGLNRTGKSCRLRWVNYLHPGLKRGKMTPQEERLVLELHAKWGNRWSKIARKLPGRTDNEIKNYWRTHMRKKAQEKKRPVSPTSSFSNCSSSSVTTTTTNTQDTSCHSRKSSGEVSFYDTGGSRSTREMNQENEDVYSLDDIWREIDHSAVNIIKPVKDIYSEQSHCLSYPNLASPSWESSLDSIWNMDADKSKISSYFANDQFPFCFQHSRSPWSSG

>AtMYB49____At5g54230

MGKSSSSEESEVKKGPWTPEEDEKLVGYIQTHGPGKWRTLPKNAGLKRCGKSCRLRWTNYLRPDIKRGEFSLQEEETIIQLHRLLGNKWSAIAIHLPGRTDNEIKNYWNTHIKKKLLRMGIDPVTHCPRINLLQLSSFLTSSLFKSMSQPMNTPFDLTTSNINPDILNHLTASLNNVQTESYQPNQQLQNDLNTDQTTFTGLLNSTPPVQWQNNGEYLGDYHSYTGTGDPSNNKVPQAGNYSSAAFVSDHINDGENFKAGWNFSSSMLAGTSSSSSTPLNSSSTFYVNGGSEDDRESFGSDMLMFHHHHDHNNNALNLS

>AtMYB50____At1g57560

MKRHSCCYKQKLRKGLWSPEEDEKLLNYITKHGHGCWSSVPKLAGLERCGKSCRLRWINYLRPDLKRGAFSSEEQNLIVELHAVLGNRWSQIAARLPGRTDNEIKNLWNSCIKKKLMKKGIDPITHKPLSEVGKETNRSDNNNSTSFSSETNQDLFVKKTSDFAEYSAFQKEESNSVSLRNSLSSMIPTQFNIDDGSVSNAGFDTQVCVKPSIILLPPPNNTSSTVSGQDHVNVSEPNWESNSGTTSHLNNPGMEEMKWSEEYLNESLFSTQVYVKSETDFNSNIAFPWSQSQACDVFPKDLQRMAFSFGGQSFFPWVTTLRRGRQQVIGFSVDQIDRTTNSFKIVIINEVRNSNETTYEFEINVGYSWKLSETTLTCCTSNLDDRMKKPVYMKGGLHWLRNDGAIVAFNPETEKARLISIRFPKELCSKTLFTAADNNLILISATEEVFYVYAVENILTDPKWVVLKQIRNGVLDEKMLYSWYPEAYDGKCLMLREILKKDHYKQVLHGYDLRANKWEVIGSIPGWYTSALDFYQFTPSLSSVIGPDAKEEEEILACDHKKISSINSIIRMLDGISS

>AtMYB51____At1g18570

MVRTPCCKAELGLKKGAWTPEEDQKLLSYLNRHGEGGWRTLPEKAGLKRCGKSCRLRWANYLRPDIKRGEFTEDEERSIISLHALHGNKWSAIARGLPGRTDNEIKNYWNTHIKKRLIKKGIDPVTHKGITSGTDKSENLPEKQNVNLTTSDHDLDNDKAKKNNKNFGLSSASFLNKVANRFGKRINQSVLSEIIGSGGPLASTSHTTNTTTTSVSVDSESVKSTSSSFAPTSNLLCHGTVATTPVSSNFDVDGNVNLTCSSSTFSDSSVNNPLMYCDNFVGNNNVDDEDTIGFSTFLNDEDFMMLEESCVENTAFMKELTRFLHEDENDVVDVTPVYERQDLFDEIDNYFG

>AtMYB52____At1g17950

MMCSRGHWRPAEDEKLRELVEQFGPHNWNAIAQKLSGRSGKSCRLRWFNQLDPRINRNPFTEEEEERLLASHRIHGNRWSVIARFFPGRTDNAVKNHWHVIMARRGRERSKLRPRGLGHDGTVAATGMIGNYKDCDKERRLATTTAINFPYQFSHINHFQVLKEFLTGKIGFRNSTTPIQEGAIDQTKRPMEFYNFLQVNTDSKIHELIDNSRKDEEEDVDQNNRIPNENCVPFFDFLSVGNSASQGLC

>AtMYB53

MGRSPSSDETGLKKGPWLPEEDDKLINYIHKHGHSSWSALPKLAGLNRCGKSCRLRWTNYLRPDIKRGKFSAEEEETILNLHAVLGNKWSMIASHLPGRTDNEIKNFWNTHLKKKLIQMGFDPMTHQPRTDDIFSSLSQLMSLSNLRGLVDLQQQFPMEDQALLNLQTEMAKLQLFQYLLQPSPAPMSINNINPNILNLLIKENSVTSNIDLGFLSSHLQDFNNNNLPSLKTLDDNHFSQNTSPIWLHEPPSLNQTMLPTHDPCAQSVDGFGSNQASSSHDQEVAVTDSVDWPDHHLFDDSMFPDISYQS

>AtMYB54____At1g73410

MIMCSRGHWRPAEDEKLKDLVEQYGPHNWNAIALKLPGRSGKSCRLRWFNQLDPRINRNPFTEEEEERLLAAHRIHGNRWSIIARLFPGRTDNAVKNHWHVIMARRTRQTSKPRLLPSTTSSSSLMASEQIMMSSGGYNHNYSSDDRKKIFPADFINFPYKFSHINHLHFLKEFFTGKIALNHKANQSKKPMEFYNFLQVNTDSNKSEIIDQDSGQSKRSDSDTKHESHVPFFDFLSVGNSAS

>AtMYB55____At4g01680

MGRHSCCYKQKLRKGLWSPEEDEKLLRYITKYGHGCWSSVPKQAGLQRCGKSCRLRWINYLRPDLKRGAFSQDEENLIIELHAVLGNRWSQIAAQLPGRTDNEIKNLWNSCLKKKLRLRGIDPVTHKLLTEIETGTDDKTKPVEKSQQTYLVETDGSSSTTTCSTNQNNNTDHLYTGNFGFQRLSLENGSRIAAGSDLGIWIPQTGRNHHHHVDETIPSAVVLPGSMFSSGLTGYRSSNLGLIELENSFSTGPMMTEHQQIQESNYNNSTFFGNGNLNWGLTMEENQNPFTISNHSNSSLYSDIKSETNFFGTEATNVGMWPCNQLQPQQHAYGHI

>AtMYB56____At5g17800

MNPNLLEKDLRGKETTNGSIRYKEANNFRSLPNSHTAACKTSLNNPSISRNHPHNKSASVLESEDEHGNERGENEKSLRMRGKSGINTKVCSRGHWRPTEDAKLKELVAQFGPQNWNLISNHLLGRSGKSCRLRWFNQLDPRINKRAFTEEEEFRLLAAHRAYGNKWALISRLFPGRTDNAVKNHWHVIMARRTRESQRQRQQPPPTLSRDAEMTVSSSCRYNQGKFINEEDDDDDVSAVSTCTTELSLTPPSSAYQPRFFNYDSTLASGKDGQCVQRAEVNGIYGKKMDHQNHHTISVSERKVEMKMRSGYYYFDFLGVGAS

>AtMYB57_____At3g01530

METTMKKKGRVKATITSQKEEEGTVRKGPWTMEEDFILFNYILNHGEGLWNSVAKASGLKRTGKSCRLRWLNYLRPDVRRGNITEEEQLLIIQLHAKLGNRWSKIAKHLPGRTDNEIKNFWRTKIQRHMKVSSENMMNHQHHCSGNSQSSGMTTQGSSGKAIDTAESFSQAKTTTFNVVEQQSNENYWNVEDLWPVHLLNGDHHVI

>AtMYB58_____At1g16490

MGKGRAPCCDKTKVKRGPWSHDEDLKLISFIHKNGHENWRSLPKQAGLLRCGKSCRLRWINYLRPDVKRGNFSAEEEDTIIKLHQSFGNKWSKIASKLPGRTDNEIKNVWHTHLKKRLSSETNLNADEAGSKGSLNEEENSQESSPNASMSFAGSNISSKDDDAQISQMFEHILTYSEFTGMLQEVDKPELLEMPFDLDPDIWSFIDGSDSFQQPENRALQESEEDEVDKWFKHLESELGLEENDNQQQQQQHKQGTEDEHSSSLLESYELLIH

>AtMYB59______At5g59780

MKLVQEEYRKGPWTEQEDILLVNFVHLFGDRRWDFVAKVSGLNRTGKSCRLRWVNYLHPGLKRGKMTPQEERLVLELHAKWGNRWSKIARKLPGRTDNEIKNYWRTHMRKKAQEKKRPMSPTSSSSNCCSSSMTTTTSQDTGGSNGKMNQECEDGYYSMDDIWREIDQSGANVIKPVKDNYYSEQSCYLNFPPLASPTWESSLESIWNMDADESKMSSFAIDQFPLSFEHGSGRL

>AtMYB60_____At1g08810

MGRPPCCDKIGIKKGPWTPEEDIILVSYIQEHGPGNWRSVPTNTGLLRCSKSCRLRWTNYLRPGIKRGNFTPHEEGMIIHLQALLGNKWASIASYLPQRTDNDIKNYWNTHLKKKLNKSDSDERSRSENIALQTSSTRNTINHRSTYASSTENISRLLEGWMRASPKSSTSTTFLEHKMQNRTNNFIDHHSDQFPYEQLQGSWEEGHSKGINGDDDQGIKNSENNNGDDVHHEDGDHEDDDDHNATPPLTFIEKWLLEETSTTGGQMEEMSHLMELSNML

>AtMYB61

MGRHSCCYKQKLRKGLWSPEEDEKLLTHITNHGHGCWSSVPKLAGLQRCGKSCRLRWINYLRPDLKRGAFSPEEENLIVELHAVLGNRWSQIASRLPGRTDNEIKNLWNSSIKKKLKQRGIDPNTHKPISEVESFSDKDKPTTSNNKRSGNDHKSPSSSSATNQDFFLERPSDLSDYFGFQKLNFNSNLGLSVTTDSSLCSMIPPQFSPGNMVGSVLQTPVCVKPSISLPPDNNSSSPISGGDHVKLAAPNWEFQTNNNNTSNFFDNGGFSWSIPNSSTSSSQVKPNHNFEEIKWSEYLNTPFFIGSTVQSQTSQPIYIKSETDYLANVSNMTDPWSQNENLGTTETSDVFSKDLQRMAVSFDFMYSILDDSSERSVFAINLLKNNDERNKSVSVCFRERIVIHSHILHHFSDL

>AtMYB62_____At1g68320

MENSMKKKKSFKESEDEELRRGPWTLEEDTLLTNYILHNGEGRWNHVAKCAGLKRTGKSCRLRWLNYLKPDIRRGNLTPQEQLLILELHSKWGNRWSKIAQYLPGRTDNEIKNYWRTRVQKQARQLNIESNSDKFFDAVRSFWVPRLIEKMEQNSSTTTTYCCPQNNNNNSLLLPSQSHDSLSMQKDIDYSGFSNIDGSSSTSTCMSHLTTVPHFMDQSNTNIIDGSMCFHEGNVQEFGGYVPGMEDYMVNSDISMECHVADGYSAYEDVTQDPMWNVDDIWQFRE

>AtMYB63_____At1g79180

MGKGRAPCCDKTKVKRGPWSPEEDIKLISFIQKFGHENWRSLPKQSGLLRCGKSCRLRWINYLRPDLKRGNFTSEEEETIIKLHHNYGNKWSKIASQLPGRTDNEIKNVWHTHLKKRLAQSSGTADEPASPCSSDSVSRGKDDKSSHVEDSLNRETNHRNELSTSMSSGGSNQQDDPKIDELRFEYIEEAYSEFNDIIIQEVDKPDLLEIPFDSDPDIWSFLDTSNSFQQSTANENSSGSRATTEEESDEDEVKKWFKHLESELGLEEDDNQQQYKEEESSSSSLLKNYELMIH

>AtMYB64____At5g11050

MEEQKIQEKSLAHGAAPPLTAVERFLNGQKNEALCFKKQERSIDRPIVKTTRAIEIRNENKENMMFGPRKEKNLAVIGEIVVKGAAKDYTCKDITKKQPYKNIIKGQWTADEDRKLIKLVMQHGERKWAVISEKLEGRAGKQCRERWHNHLRPDIKKDSWSEEEERLLVEAHTRIGNKWAEIAKLIQGRTENSIKNHWNATKRRQNSKRKHKRSKNADSNSDIDDLSPSAKRPRILEDYIKNIENNDKNNGENIMTTSGNNVLSTSNYDQFNSEDSTSSLLDDPYDEELVFLKNIFENHSLENINLSQGTEITQSSSSGFMIENPKPKPNLYNNTFGTHLGAMVTEPANSSHLASDIYLSDLLNGTASSSSSLTFLSSNNNEHAGENELLLPQANSTSERREMDLIEMLSGSTQGSNIWFPLF

>AtMYB65

MSYTTATADSDDGMHSSIHNESPAPDSISNGCRSRGKRSVLKKGPWTSTEDGILIDYVKKHGEGNWNAVQKHTSLARCGKSCRLRWANHLRPNLKKGAFSQEEEQLIVEMHAKMGNKWAQMAEHLPGRTDNEIKNYWNTRIKRRQRAGLPLYPPEIYVDDLHWSEEYTKSNIIRVDRRRRHQDFLQLGNSKDNVLFDDLNFAASLLPAASDLSDLVACNMLGTGASSSRYESYMPPILPSPKQIWESGSRFPMCSSNIKHEFQSPEHFQNTAVQKNPRSCSISPCDVDHHPYENQHSSHMMMVPDSHTVTYGMHPTSKPLFGAVKLELPSFQYSETSAFDQWKTTPSPPHSDLLDSVDAYIQSPPPSQVEESDCFSSCDTGLLDMLLHEAKIKTSAKHSLLMSSPQKSFSSTTCTTNVTQNVPRGSENLIKSGEYEDSQKYLGRSEITSPSQLSAGGFSSAFAGNVVKTEELDQVWEPKRVDITRPDVLLASSWLDQGCYGIVSDTSSMSDALALLGGDDIGNSYVTVGSSSGQAPRGVGSYGWTNMPPVWSL

>AtMYB66_____At5g14750

MRKKVSSSGDEGNNEYKKGLWTVEEDKILMDYVKAHGKGHWNRIAKKTGLKRCGKSCRLRWMNYLSPNVKRGNFTEQEEDLIIRLHKLLGNRWSLIAKRVPGRTDNQVKNYWNTHLSKKLGIKDQKTKQSNGDIVYQINLPNPTETSEETKISNIVDNNNILGDEIQEDHQGSNYLSSLWVHEDEFELSTLTNMMDFIDGHCF

>AtMYB67____At3g12720

MGHRCCGKHKVKRGLWSPEEDEKLLRYITTHGHPSWSSVPKLAGLQRCGKSCRLRWINYLRPDLRRGSFNEEEEQIIIDVHRILGNKWAQIAKHLPGRTDNEVKNFWNSCIKKKLLSQGLDPSTHNLMPSHKRSSSSNNNNIPKPNKTTSIMKNPTDLDQSTTAFSITNINPPTSTKPNKLKSPNQTTIPSQTVIPINDNMSSTQTMIPINDPMSSLLDDENMIPHWSDVDGMAIHEAPMLPSDKAVVGVDDDDLNMDILFNTPSSSAFDPDFASIFSSAMSIDFNPMDDLGSWTF

>AtMYB68______At5g65790

MGRAPCCDKANVKKGPWSPEEDAKLKDYIENSGTGGNWIALPQKIGLRRCGKSCRLRWLNYLRPNIKHGGFSEEEDNIICNLYVTIGSRWSIIAAQLPGRTDNDIKNYWNTRLKKKLLNKQRKEFQEARMKQEMVMMKRQQQGQGQGQSNGSTDLYLNNMFGSSPWPLLPQLPPPHHQIPLGMMEPTSCNYYQTTPSCNLEQKPLITLKNMVKIEEEQERTNPDHHHQDSVTNPFDFSFSQLLLDPNYYLGSGGGGEGDFAIMSSSTNSPLPNTSSDQHPSQQQEILQWFGSSNFQTEAINDMFINNNNNIVNLETIENTKVYGDASVAGAAVRAALGGGTTSTSADQSTISWEDITSLVNSEDASYFNAPNHV

>AtMYB69_____At4g33450

MEMSRGSNSFDNKKPSCQRGHWRPVEDDNLRQLVEQYGPKNWNFIAQHLYGRSGKSCRLRWYNQLDPNITKKPFTEEEEERLLKAHRIQGNRWASIARLFPGRTDNAVKNHFHVIMARRKRENFSSTATSTFNQTWHTVLSPSSSLTRLNRSHFGLWRYRKDKSCGLWPYSFVSPPTNGQFGSSSVSNVHHEIYLERRKSKELVDPQNYTFHAATPDHKMTSNEDGPSMGDDGEKNDVTFIDFLGVGLAS

>AtMYB70_____At2g23290

MSGSTRKEMDRIKGPWSPEEDDLLQSLVQKHGPRNWSLISKSIPGRSGKSCRLRWCNQLSPEVEHRGFTAEEDDTIILAHARFGNKWATIARLLNGRTDNAIKNHWNSTLKRKCSGGGGGGEEGQSCDFGGNGGYDGNLTDEKPLKRRASGGGGVVVVTALSPTGSDVSEQSQSSGSVLPVSSSCHVFKPTARAGGVVIESSSPEEEEKDPMTCLRLSLPWVNESTTPPELFPVKREEEEEKEREISGLGGDFMTVVQEMIKTEVRSYMADLQLGNGGGAGGGASSCMVQGTNGRNVGFREFIGLGRIE

>AtMYB71_____At3g24310

MSLWGGMGGGWGMVEEGWRKGPWTAEEDRLLIDYVQLHGEGRWNSVARLAGLKRNGKSCRLRWVNYLRPDLKRGQITPHEETIILELHAKWGNRWSTIARSLPGRTDNEIKNYWRTHFKKKTKSPTNSAEKTKNRILKRQQFQQQRQMELQQEQQLLQFNQIDMKKIMSLLDDDNNNGDNTFSSSSSGESGALYVPHQITHSTTTSGCEPNSNGYYPVVPVTIPEANVNEDNAIWDGLWNLDFEGQGSFGGAACAPRKHYFQNMVIPFC

>AtMYB72_____At1g56160

MGKGRAPCCDKNKVKRGPWSPQEDLTLITFIQKHGHQNWRSLPKLAGLLRCGKSCRLRWINYLRPDVKRGNFSKKEEDAIIHYHQTLGNKWSKIASFLPGRTDNEIKNVWNTHLKKRLTPSSSSSSLSSTHDQSTKADHDKNCDGAQEEIHSGLNESQNSATSSHHQGECMHTKPELHEVNGLNEIQFLLDHDDFDDITSEFLQDNDILFPLDSLLHNHQTHISTQEMTREVTKSQSFDHPQPDIPCGFEDTNEESDLRRQLVESTTPNNEYDEWFNFIDNQTYFDDFNFVGEVCL

>AtMYB73_____At4g37260

MSNPTRKNMERIKGPWSPEEDDLLQRLVQKHGPRNWSLISKSIPGRSGKSCRLRWCNQLSPEVEHRAFSQEEDETIIRAHARFGNKWATISRLLNGRTDNAIKNHWNSTLKRKCSVEGQSCDFGGNGGYDGNLGEEQPLKRTASGGGGVSTGLYMSPGSPSGSDVSEQSSGGAHVFKPTVRSEVTASSSGEDPPTYLSLSLPWTDETVRVNEPVQLNQNTVMDGGYTAELFPVRKEEQVEVEEEEAKGISGGFGGEFMTVVQEMIRTEVRSYMADLQRGNVGGSSSGGGGGGSCMPQSVNSRRVGFREFIVNQIGIGKME

>AtMYB74____At4g05100

MGRSPCCEKKNGLKKGPWTPEEDQKLIDYINIHGYGNWRTLPKNAGLQRCGKSCRLRWTNYLRPDIKRGRFSFEEEETIIQLHSIMGNKWSAIAARLPGRTDNEIKNYWNTHIRKRLLKMGIDPVTHTPRLDLLDISSILSSSIYNSSHHHHHHHQQHMNMSRLMMSDGNHQPLVNPEILKLATSLFSNQNHPNNTHENNTVNQTEVNQYQTGYNMPGNEELQSWFPIMDQFTNFQDLMPMKTTVQNSLSYDDDCSKSNFVLEPYYSDFASVLTTPSSSPTPLNSSSSTYINSSTCSTEDEKESYYSDNITNYSFDVNGFLQFQ

>AtMYB75___PAP1

MEGSSKGLRKGAWTTEEDSLLRQCINKYGEGKWHQVPVRAGLNRCRKSCRLRWLNYLKPSIKRGKLSSDEVDLLLRLHRLLGNRWSLIAGRLPGRTANDVKNYWNTHLSKKHEPCCKIKMKKRDITPIPTTPALKNNVYKPRPRSFTVNNDCNHLNAPPKVDVNPPCLGLNINNVCDNSIIYNKDKKKDQLVNNLIDGDNMWLEKFLEESQEVDILVPEATTTEKGDTLAFDVDQLWSLFDGETVKFD

>AtMYB76_____At5g07700

MSKRPYCIGEGLKKGAWTTEEDKKLISYIHDHGEGGWRDIPEKAGLKRCGKSCRLRWTNYLKPDIKRGEFSYEEEQIIIMLHASRGNKWSVIARHLPKRTDNEVKNYWNTHLKKRLIDDGIDPVTHKPLASSNPNPVEPMKFDFQKKSNQDEHSSQSSSTTPASLPLSSNLNSVKSKISSGETQIESGHVSCKKRFGRSSSTSRLLNKVAARASSIGNILSTSIEGTLRSPASSSGLPDSFSQSYEYMIDNKEDLGTSIDLNIPEYDFPQFLEQLINDDDENENIVGPEQDLLMSDFPSTFVDEDDILGDITSWSTYLLDHPNFMYESDQDSDEKNFL

>AtMYB77_____At3g50060

MADRVKGPWSQEEDEQLRRMVEKYGPRNWSAISKSIPGRSGKSCRLRWCNQLSPEVEHRPFSPEEDETIVTARAQFGNKWATIARLLNGRTDNAVKNHWNSTLKRKCSGGVAVTTVTETEEDQDRPKKRRSVSFDSAFAPVDTGLYMSPESPNGIDVSDSSTIPSPSSPVAQLFKPMPISGGFTVVPQPLPVEMSSSSEDPPTSLSLSLPGAENTSSSHNNNNNALMFPRFESQMKINVEERGEGRRGEFMTVVQEMIKAEVRSYMAEMQKTSGGFVVGGLYESGGNGGFRDCGVITPKVE

>AtMYB78____At5g49620

MGDKGRSLKINKNMEEFTKVEEEMDVRRGPWTVEEDLELINYIASHGEGRWNSLARCAELKRTGKSCRLRWLNYLRPDVRRGNITLEEQLLILELHTRWGNRWSKIAQYLPGRTDNEIKNYWRTRVQKHAKQLKCDVNSQQFKDTMKYLWMPRLVERIQAASIGSVSMSSCVTTSSDQFVINNNNTNNVDNLALMSNPNGYITPDNSSVAVSPVSDLTECQVSSEVWKIGQDENLVDPKMTSPNYMDNSSGLLNGDFTKMQDQSDLNWFENINGMVPNYSDSFWNIGNDEDFWLLQQHQQVHDNGSF

>AtMYB79_____At4g13480

MVEEVWRKGPWTAEEDRLLIEYVRVHGEGRWNSVSKLAGLKRNGKSCRLRWVNYLRPDLKRGQITPHEESIILELHAKWGNRWSTIARSLPGRTDNEIKNYWRTHFKKKAKPTTNNAEKIKSRLLKRQHFKEQREIELQQEQQLFQFDQLGMKKIISLLEENNSSSSSDGGGDVFYYPDQITHSSKPFGYNSNSLEEQLQGRFSPVNIPDANTMNEDNAIWDGFWNMDVVNGHGGNLGVVAATAACGPRKPYFHNLVIPFC

>AtMYB80_____At5g56110

MGRIPCCEKENVKRGQWTPEEDNKLASYIAQHGTRNWRLIPKNAGLQRCGKSCRLRWTNYLRPDLKHGQFSEAEEHIIVKFHSVLGNRWSLIAAQLPGRTDNDVKNYWNTKLKKKLSGMGIDPVTHKPFSHLMAEITTTLNPPQVSHLAEAALGCFKDEMLHLLTKKRVDLNQINFSNHNPNPNNFHEIADNEAGKIKMDGLDHGNGIMKLWDMGNGFSYGSSSSSFGNEERNDGSASPAVAAWRGHGGIRTAVAETAAAEEEERRKLKGEVVDQEEIGSEGGRGDGMTMMRNHHHHQHVFNVDNVLWDLQADDLINHMV

>AtMYB81____At2g26960

MGKVRQDSGSDDDNSIKKSFTKGPWTQAEDNLLIAYVDKHGDGNWNAVQNNSGLSRCGKSCRLRWVNHLRPDLKKGAFTEKEEKRVIELHALLGNKWARMAEELPGRTDNEIKNFWNTRLKRLQRLGLPVYPDEVREHAMNAATHSGLNTDSLDGHHSQEYMEADTVEIPEVDFEHLPLNRSSSYYQSMLRHVPPTNVFVRQKPCFFQPPNVYNLIPPSPYMSTGKRPREPETAFPCPGGYTMNEQSPRLWNYPFVENVSEQLPDSHLLGNAAYSSPPGPLVHGVENFEFPSFQYHEEPGGWGADQPNPMPEHESDNTLVQSPLTAQTPSDCPSSSLYDGLLESVVYGSSGEKPATDTDSESSLFQSFTPANENITGKTCFLTLYALHALHCLCNQFKKSPLLHLHDKLNWCNKFRFNSFKSGTHIL

>AtMYB82

MECKREEGKSYVKRGLWKPEEDMILKSYVETHGEGNWADISRRSGLKRGGKSCRLRWKNYLRPNIKRGSMSPQEQDLIIRMHKLLGNRWSLIAGRLPGRTDNEVKNYWNTHLNKKPNSRRQNAPESIVGATPFTDKPVMSTELRRSHGEGGEEESNTWMEETNHFGYDVHVGSPLPLISHYPDNTLVFDPCFSFTDFFPLL

>AtMYB83_____At3g08500

MMMRKPDITTIRDKGKPNHACGGNNNKPKLRKGLWSPDEDEKLIRYMLTNGQGCWSDIARNAGLLRCGKSCRLRWINYLRPDLKRGSFSPQEEDLIFHLHSILGNRWSQIATRLPGRTDNEIKNFWNSTLKKRLKNNSNNNTSSGSSPNNSNSNSLDPRDQHVDMGGNSTSLMDDYHHDENMMTVGNTMRMDSSSPFNVGPMVNSVGLNQLYDPLMISVPDNGYHQMGNTVNVFSVNGLGDYGNTILDPISKRVSVEGDDWFIPPSENTNVIACSTSNNLNLQALDPCFNSKNLCHSESFKVGNVLGIENGSWEIENPKIGDWDLDGLIDNNSSFPFLDFQVD

>AtMYB84_____At3g49690_RAX3

MGRAPCCDKANVKKGPWSPEEDAKLKSYIENSGTGGNWIALPQKIGLKRCGKSCRLRWLNYLRPNIKHGGFSEEEENIICSLYLTIGSRWSIIAAQLPGRTDNDIKNYWNTRLKKKLINKQRKELQEACMEQQEMMVMMKRQHQQQQIQTSFMMRQDQTMFTWPLHHHNVQVPALFMNQTNSFCDQEDVKPVLIKNMVKIEDQELEKTNPHHHQDSMTNAFDHLSFSQLLLDPNHNHLGSGEGFSMNSILSANTNSPLLNTSNDNQWFGNFQAETVNLFSGASTSTSADQSTISWEDISSLVYSDSKQFF

>AtMYB85____At4g22680

MGRQPCCDKLGVKKGPWTVEEDKKLINFILTNGHCCWRALPKLAGLRRCGKSCRLRWTNYLRPDLKRGLLSHDEEQLVIDLHANLGNNYLWSDETTKDEASWSDSNFGVGGTLYDHNISGADADFPIWSPERINDEKMFLDYCQDFGVHDFGF

>AtMYB86

MGRHSCCFKQKLRKGLWSPEEDEKLLNYITRHGHGCWSSVPKLAGLQRCGKSCRLRWINYLRPDLKRGAFSQDEESLIIELHAALGNRWSQIATRLPGRTDNEIKNFWNSCLKKKLRRKGIDPTTHKPLITNELQSLNVIDQKLTSSEVVKSTGSINNLHDQSMVVSSQQGPWWFPANTTTTNQNSAFCFSSSNTTTVSDQIVSLISSMSTSSSPTPMTSNFSPAPNNWEQLNYCNTVPSQSNSIYSAFFGNQYTEASQTMNNNNPLVDQHHHHQDMKSWASEILHYTEHNQSSETVIEAEVKPDIANYYWRSASSSSSPNQEAATLLHDANVEVYGKNLQKLNNMVFDQSL

>AtMYB87_____At4g37780

MAVKKGPWSTEEDAVLKSYIEKHGTGNNWISLPQRIGIKRCGKSCRLRWLNYLRPNLKHGGFTDEEDYIICSLYITIGSRWSIIASQLPGRTDNDIKNYWNTRLKKKLLSKQGKAFHQQLNVKFERGTTSSSSSQNQIQIFHDENTKSNQTLYNQVVDPSMRAFAMEEQSMIKNQILEPFSWEPNKVLFDVDYDAAASSYHHHASPSLNSMSSTSSIGTNNSSLQMSHYTVNHNDHDQPDMFFMDGFENFQAELFDEIANNNTVENGFDGTEILINNNYLDHDISSFIDYPLYDNE

>AtMYB88_____At2g02820

MKKKKKILLHSDDSKKKERHIVTWSPEEDDILRKQISLQGTENWAIIASKFNDKSTRQCRRRWYTYLNSDFKRGGWSPEEDTLLCEAQRLFGNRWTEIAKVVSGRTDNAVKNRFTTLCKKRAKHEAMAKENRIACCVNSDNKRLLFPDGISTPLKAESESPLTKKMRRSHIPNLTEIKSYGDRSHIKVESTMNQQRRHPFSVVAHNATSSDGTEEQKQIGNVKESDGEDKSNQEVFLKKDDSKVTALMQQAELLSSLAQKVNADNTDQSMENAWKVLQDFLNKSKENDLFRYGIPDIDFQLDEFKDLVEDLRSSNEDSQSSWRQPDLHDSPASSEYSSGSGSGSTIMTHPSGDKTQQLMSDTQTTSHQQNGGELLQDNGIVSDATVEQVGLLSTGHDVLKNSNETVPIPGEEEFNSPVQVTPLFRSLAAGIPSPQFSESVSIHLTN

>AtMYB89____At5g39700

MYLFMYKCNIVLEETHVFQNTPCDVSLQRPFNGFGENNALPLRKMHQEEKKKKHRGGHWTLSEDLKLKELVAVFGPQNWKFIGEKMEPRTSLSCRQRWFNQLDPKINKRNFTDEEEEKLLRAHILYGNKWSKIAKLFNRRTDHAVKNHWHSLMNRIIRKQSASDIRSFDNIQNYQTSNFLPGSVSHESTNLNAQYLQEKNYSSCMPLQHSCHHHHFSTFPANSLALFTPHVSISQPSSSSLLSSSEAEDIMTTKTPRYIDFLGVGDS

>AtMYB90____At1g66390_PAP2

MEGSSKGLRKGAWTAEEDSLLRLCIDKYGEGKWHQVPLRAGLNRCRKSCRLRWLNYLKPSIKRGRLSNDEVDLLLRLHKLLGNRWSLIAGRLPGRTANDVKNYWNTHLSKKHESSCCKSKMKKKNIISPPTTPVQKIGVFKPRPRSFSVNNGCSHLNGLPEVDLIPSCLGLKKNNVCENSITCNKDDEKDDFVNNLMNGDNMWLENLLGENQEADAIVPEATTAEHGATLAFDVEQLWSLFDGETVELD

>AtMYB91____AS1

MKERQRWSGEEDALLRAYVRQFGPREWHLVSERMNKPLNRDAKSCLERWKNYLKPGIKKGSLTEEEQRLVIRLQEKHGNKWKKIAAEVPGRTAKRLGKWWEVFKEKQQREEKESNKRVEPIDESKYDRILESFAEKLVKERSNVVPAAAAAATVVMANSNGGFLHSEQQVQPPNPVIPPWLATSNNGNNVVARPPSVTLTLSPSTVAAAAPQPPIPWLQQQQPERAENGPGGLVLGSMMPSCSGSSESVFLSELVECCRELEEGHRAWADHKKEAAWRLRRLELQLESEKTCRQREKMEEIEAKMKALREEQKNAMEKIEGEYREQLVGLRRDAEAKDQKLADQWTSRHIRLTKFLEQQMGCRLDRP

>AtMYB92____At5g10280

MGRSPISDDSGLKKGPWTPDEDEKLVNYVQKHGHSSWRALPKLAGLNRCGKSCRLRWTNYLRPDIKRGRFSPDEEQTILNLHSVLGNKWSTIANQLPGRTDNEIKNFWNTHLKKKLIQMGFDPMTHRPRTDIFSGLSQLMSLSSNLRGFVDLQQQFPIDQEHTILKLQTEMAKLQLFQYLLQPSSMSNNVNPNDFDTLSLLNSIASFKETSNNTTSNNLDLGFLGSYLQDFHSLPSLKTLNSNMEPSSVFPQNLDDNHFKFSTQRENLPVSPIWLSDPSSTTPAHVNDDLIFNQYGIEDVNSNITSSSGQESGASASAAWPDHLLDDSIFSDIP

>AtMYB93____At1g34670

MGRSPCCDENGLKKGPWTPEEDQKLIDYIHKHGHGSWRALPKLADLNRCGKSCRLRWTNYLRPDIKRGKFSAEEEQTILHLHSILGNKWSAIATHLQGRTDNEIKNFWNTHLKKKLIQMGIDPVTHQPRTDLFASLPQLIALANLKDLIEQTSQFSSMQGEAAQLANLQYLQRMFNSSASLTNNNGNNFSPSSILDIDQHHAMNLLNSMVSWNKDQNPAFDPVLELEANDQNQDLFPLGFIIDQPTQPLQQQKYHLNNSPSELPSQGDPLLDHVPFSLQTPLNSEDHFIDNLVKHPTDHEHEHDDNPSSWVLPSLIDNNPKTVTSSLPHNNPADASSSSSYGGCEAASFYWPDICFDESLMNVIS

>AtMYB94______At3g47600

MGRPPCCDKIGVKKGPWTPEEDIILVSYIQEHGPGNWRSVPTHTGLRRCSKSCRLRWTNYLRPGIKRGNFTEHEEKMILHLQALLGNRWAAIASYLPERTDNDIKNYWNTHLKKKLKKMNDSCDSTINNGLDNKDFSISNKNTTSHQSSNSSKGQWERRLQTDINMAKQALCDALSIDKPQNPTNFSIPDLGYGPSSSSSSTTTTTTTTRNTNPYPSGVYASSAENIARLLQNFMKDTPKTSVPLPVAATEMAITTAASSPSTTEGDGEGIDHSLFSFNSIDEAEEKPKLIDHDINGLITQGSLSLFEKWLFDEQSHDMIINNMSLEGQEVLF

>AtMYB95____At1g74430

MGRTTWFDVDGLRKGEWTAEEDRKLVVYINEHGLGEWGSLPKRAGLQRCGKSCRLRWLNYLRPGIKRGKFTPQEEEEIIKYHALLGNRWAAIAKQMPNRTDNDIKNHWNSCLKKRLAKKGIDPMTHEPTTTTSLTVDVTSSSTTSSPTPSPTSSSFSSCSSTGSARFLNKLAAGISSRKHGLESIKTVILAEQPREAVDEEKMMTINMKEKELISCYMEIDETMSIDELPCDDSTSGFVAFDDYSLIDPYRDGVYVSDFYDETEHLDLFLL

>AtMYB96

MGRPPCCEKIGVKKGPWTPEEDIILVSYIQEHGPGNWRSVPTHTGLRCSKSCRLRWTNYLRPGIKRGNFTEHEEKTIVHLQALLGNRWAAIASYLPERTDNDIKNYWNTHLKKKLKKINESGEEDNDGVSSSNTSSQKNHQSTNKGQWERRLQTDINMAKQALCEALSLDKPSSTLSSSSSLPTPVITQQNIRNFSSALLDRCYDPSSSSSSTTTTTTSNTTNPYPSGVYASSAENIARLLQDFMKDTPKALTLSSSSPVSETGPLTAAVSEEGGEGFEQSFFSFNSMDETQNLTQETSFFHDQVIKPEITMDQDHGLISQGSLSLFEKWLFDEQSHEMVGMALAGQEGMF

>AtMYB97____At4g26930

MIVYGGGASEDGEGGGVVLKKGPWTVAEDETLAAYVREYGEGNWNSVQKKTWLARCGKSCRLRWANHLRPNLRKGSFTPEEERLIIQLHSQLGNKWARMAAQLPGRTDNEIKNYWNTRLKRFQRQGLPLYPPEYSQNNHQQQMYPQQPSSPLPSQTPASSFTFPLLQPPSLCPKRCYNTAFSPKASYISSPTNFLVSSPTFLHTHSSLSSYQSTNPVYSMKHELSSNQIPYSASLGVYQVSKFSDNGDCNQNLNTGLHTNTCQLLEDLMEEAEALADSFRAPKRRQIMAALEDNNNNNNFFSGGFGHRVSSNSLCSLQGLTPKEDESLQMNTMQDEDITKLLDWGSESEEISNGQSSVITTENNLVLDDHQFAFLFPVDDDTNNLPGIC

>AtMYB98____At4g18770_PHAN

MENFVDENGFASLNQNIFTRDQEHMKEEDFPFEVVDQSKPTSFLQDFHHLDHDHQFDHHHHHGSSSSHPLLSVQTTSSCINNAPFEHCSYQENMVDFYETKPNLMNHHHFQAVENSYFTRNHHHHQEINLVDEHDDPMDLEQNNMMMMRMIPFDYPPTETFKPMNFVMPDEISCVSADNDCYRATSFNKTKPFLTRKLSSSSSSSSWKETKKSTLVKGQWTAEEDRVLIQLVEKYGLRKWSHIAQVLPGRIGKQCRERWHNHLRPDIKKETWSEEEDRVLIEFHKEIGNKWAEIAKRLPGRTENSIKNHWNATKRRQFSKRKCRSKYPRPSLLQDYIKSLNMGALMASSVPARGRRRESNNKKKDVVVAVEEKKKEEEVYGQDRIVPECVFTDDFGFNEKLLEEGCSIDSLLDDIPQPDIDAFVHGL

>AtMYB99

MGGRKPCCDEVGLRKGPWTVEEDGKLVDFLRARGNCGGGGGGWCWRDVPKLAGLRRCGKSCRLRWTNYLRPDLKRGLFTEEEIQLVIDLHARLGNRWSKIAVELPGRTDNDIKNYWNTHIKRKLIRMGIDPNTHRRFDQQKVNEEETILVNDPKPLSETEVSVALKNDTSAVLSGNLNQLADVDGDDQPWSFLMENDEGGGGDAAGELTMLLSGDITSSCSSSSSLWMKYGEFGYEDLELGCFDV

>AtMYB100

MKKNYQKKNIKVVSTSKYLKKSDIDKVNWTESEDIKLKEIMALGPKNKWTKVAKKFEGRTGKQCRERWYNHARPNIKKTAWSEEEDQILIEAHKVLGTKWVEIAQQLPGRSDNNIKNHWNTTKRRVQNKRGGTVNPVGNNILENYIRCITINNEDFLKTDGSYGEPTNIESDDDSKDMLYGEMNLSLETITTQTTKPLTNASTTSPYVQMPEDNYTMEDCESLEDILELLRWWE

>AtMYB101____AtM1_At2g32460

MDGGGETTATATMEGRGLKKGPWTTTEDAILTEYVRKHGEGNWNAVQKNSGLLRCGKSCRLRWANHLRPNLKKGSFTPDEEKIIIDLHAKLGNKWARMASQLPGRTDNEIKNYWNTRMKRRQRAGLPLYPHEIQHQGIDIDDEFEFDLTSFQFQNQDLDHNHQNMIQYTNSSNTSSSSSSFSSSSSQPSKRLRPDPLVSTNPGLNPIPDSSMDFQMFSLYNNSLENDNNQFGFSVPLSSSSSSNEVCNPNHILEYISENSDTRNTNKKDIDAMSYSSLLMGDLEIRSSSFPLGLDNSVLELPSNQRPTHSFSSSPIIDNGVHLEPPSGNSGLLDALLEESQALSRGGLFKDVRVSSSDLCEVQDKRVKMDFENLLIDHLNSSNHSSLGANPNIHNKYNEPTMVKVTVDDDDELLTSLLNNFPSTTTPLPDWYRVTEMQNEASYLAPPSGILMGNHQGNGRVEPPTVPPSSSVDPMASLGSCYWSNMPSIC

>AtMYB102____AtM4_At4g21440

MARSPCCEKNGLKKGPWTSEEDQKLVDYIQKHGYGNWRTLPKNAGTCLQRCGKSCRLRWTNYLRPDIKRGRFSFEEEETIIQLHSFLGNKWSAIAARLPGRTDNEIKNFWNTHIRKKLLRMGIDPVTHSPRLDLLDISSILASSLYNSSSHHMNMSRLMMDTNRRHHQQHPLVNPEILKLATSLFSQNQNQNLVVDHDSRTQEKQTVYSQTGVNQYQTNQYFENTITQELQSSMPPFPNEARQFNNMDHHFNGFGEQNLVSTSTTSVQDCYNPSFNDYSSSNFVLDPSYSDQSFNFANSVLNTPSSSPSPTTLNSSYINSSSCSTEDEIESYCSNLMKFDIPDFLDVNGFII

>AtMYB103

MGHHSCCNQQKVKRGLWSPEEDEKLIRYITTHGYGCWSEVPEKAGLQRCGKSCRLRWINYLRPDIRRGRFSPEEEKLIISLHGVVGNRWAHIASHLPGRTDNEIKNYWNSWIKKKIRKPHHHYSRHQPSVTTVTLNADTTSIATTIEASTTTTSTIDNLHFDGFTDSPNQLNFTNDQETNIKIQETFFSHKPPLFMVDTTLPILEGMFSENIITNNNKNNDHDDTQRGGRENVCEQAFLTTNTEEWDMNLRQQEPFQVPTLASHVFNNSSNSNIDTVISYNLPALIEGNVDNIVHNENSNVQDGEMASTFECLKRQELSYDQWDDSQQCSNFFFWDNLNINVEGSSLVGNQDPSMNLGSSALSSSFPSSF

>AtMYB104____At2g26950

MKKTFTKSKWKPEEDRILKDYVIQYGDRTWTHVPKRTGLPHNPASCRFRWMNHLKPSLKKGPFTDEEEKRVLQLHAVLGNKWSQMAREFPGRTDNEIKNFWNARRMRLKGKGLPVYPDEVREQAIRTAAQYGVKVELLNAHYSQDSLMAGNVEKPQELNNLALNQCSPYYQSTLANVQPSRNRVMEPETTFPFTGGSAMNEQNPTLLCNPYVESTQEQLPDSHLFGNVTYSSPPMPLIHEVENLELPSFQGFDFHEEPSSFGAEQYNPMLNLEPHNTLVQSPLIGQTPTDFPSSFYDELLDELLESVVNGSLGEIPKTDTSSESQLFQSSLRSHTDATPDIANTTGKPFLVSTHYITITSFFVCSIQEVSTNLYA

>AtMYB105_____At1g69560_LOF2

MNPNLSDYSNCNKKDTTVYRSCGHSSKASVSRGHWRPAEDTKLKELVAVYGPQNWNLIAEKLQGRSGKSCRLRWFNQLDPRINRRAFTEEEEERLMQAHRLYGNKWAMIARLFPGRTDNSVKNHWHVIMARKFREQSSSYRRRKTMVSLKPLINPNPHIFNDFDPTRLALTHLASSDHKQLMLPVPCFPGYDHENESPLMVDMFETQMMVGDYIAWTQEATTFDFLNQTGKSEIFERINEEKKPPFFDFLGLGTV

>AtMYB106____At3g01140_NOECK

MGRSPCCDKAGLKKGPWTPEEDQKLLAYIEEHGHGSWRSLPEKAGLQRCGKSCRLRWTNYLRPDIKRGKFTVQEEQTIIQLHALLGNRWSAIATHLPKRTDNEIKNYWNTHLKKRLIKMGIDPVTHKHKNETLSSSTGQSKNAATLSHMAQWESARLEAEARLARESKLLHLQHYQNNNNLNKSAAPQQHCFTQKTSTNWTKPNQGNGDQQLESPTSTVTFSENLLMPLGIPTDSSRNRNNNNNESSAMIELAVSSSTSSDVSLVKEHEHDWIRQINCGSGGIGEGFTSLLIGDSVGRGLPTGKNEATAGVGNESEYNYYEDNKNYWNSILNLVDSSPSDSATMF

>AtMYB107____At3g02940

MGRSPCCDESGLKKGPWTPEEDQKLINHIRKHGHGSWRALPKQAGLNRCGKSCRLRWTNYLRPDIKRGNFTAEEEQTIINLHSLLGNKWSSIAGHLPGRTDNEIKNYWNTHIRKKLIQMGIDPVTHRPRTDHLNVLAALPQLLAAANFNNLLNLNQNIQLDATSVAKAQLLHSMIQVLSNNNTSSSFDIHHTTNNLFGQSSFLENLPNIENPYDQTQGLSHIDDQPLDSFSSPIRVVAYQHDQNFIPPLISTSPDESKETQMMVKNKEIMKYNDHTSNPSSTSTFTQDHQPWCDIIDDEASDSYWKEIIEQTCSEPWPFRE

>AtMYB108____At3g06490_BOS1

MDEKGRSLKNNNMEDEMDLKRGPWTAEEDFKLMNYIATNGEGRWNSLSRCAGLQRTGKSCRLRWLNYLRPDVRRGNITLEEQLLILELHSRWGNRWSKIAQYLPGRTDNEIKNYWRTRVQKHAKQLKCDVNSQQFKDTMKYLWMPRLVERIQSASASSAAAATTTTTTTTGSAGTSSCITTSNNQFMNYDYNNNNMGQQFGVMSNNDYITPENSSVAVSPASDLTEYYSAPNPNPEYYSGQMGNSYYPDQNLVSSQLLPDNYFDYSGLLDEDLTAMQEQSNLSWFENINGAASSSDSLWNIGETDEEFWFLQQQQQFNNNGSF

>AtMYB109_____At3g55730

MEGETHQSEPLPLASGDSDEGISAAIEAELAELAAGDSSGGGGCGGGGGGIRSKVKGPWSTEEDAVLTKLVRKLGPRNWSLIARGIPGRSGKSCRLRWCNQLDPCLKRKPFSDEEDRMIISAHAVHGNKWAVIAKLLTGRTDNAIKNHWNSTLRRKYADLWNNGQWMANSVTTASVKNENVDETTNPPSSKQQLPQGDINSSPPKPPQVSDVVMEEAANEPQEPQEQQEQAPPVVSNVPTENNVFRPVARVGAFSIYNPTSQKNGYRDYNIVPCEGPLIQAAKPDSLAGKFLQSLCDEPQIPSKCGHGCSTLPAETKFSRNSVLGPEFVDYEEPSAVFNQELISIATDLNNIAWIKSGLDNAVVREAEQSLKMDNYNYNDPRIKFTGMMPRQDFFCARS

>AtMYB110____At3g29020

MDFSCFQEYPFEFHCRGTTFNGFRENNAVSETVEEFCNKRRMQKKSDDLKTKKKKKQSVSRVCSRGHWRISEDTQLMELVSVYGPQNWNHIAESMQGRTGKSCRLRWFNQLDPRINKRAFSDEEEERLLAAHRAFGNKWAMIAKLFNGRTDNALKNHWHVLMARKMRQQSSSYVQRFNGSAHESNTDHKIFNLSPGNVDDDEDVNLKKCSWEMLKEGTTNLKAQYLQEEYSSSRMPMQGPHHHYSTFPADSLALTLHVSIQEPSSSSSLSLPSSSTTGEHTMVTRYFETIKPPAFIDFLGVGH

>AtMYB111_____At5g49330

MGRAPCCEKIGLKRGRWTAEEDEILTKYIQTNGEGSWRSLPKKAGLLRCGKSCRLRWINYLRRDLKRGNITSDEEEIIVKLHSLLGNRWSLIATHLPGRTDNEIKNYWNSHLSRKIYAFTAVSGDGHNLLVNDVVLKKSCSSSSGAKNNNKTKKKKKGRTSRSSMKKHKQMVTASQCFSQPKELESDFSEGGQNGNFEGESLGPYEWLDGELERLLSSCVWECTSEEAVIGVNDEKVCESGDNSSCCVNLFEEEQGSETKIGHVGITEVDHDMTVEREREGSFLSSNSNENNDKDWWVGLCNSSEVGFGVDEELLDWEFQGNVTCQSDDLWDLSDIGEITLE

>AtMYB112____At1g48000

MEIEIRRGPWTVEEDMKLVSYISLHGEGRWNSLSRSAGLNRTGKSCRLRWLNYLRPDIRRGDISLQEQFIILELHSRWGNRWSKIAQHLPGRTDNEIKNYWRTRVQKHAKLLKCDVNSKQFKDTIKHLWMPRLIERIAATQSVQFTSNHYSPENSSVATATSSTSSSEAVRSSFYGGDQVEFGTLDHMTNGGYWFNGGDTFETLCSFDELNKWLIQ

>AtMYB113_____At1g66370

MGESPKGLRKGTWTTEEDILLRQCIDKYGEGKWHRVPLRTGLNRCRKSCRLRWLNYLKPSIKRGKLCSDEVDLVLRLHKLLGNRWSLIAGRLPGRTANDVKNYWNTHLSKKHDERCCKTKMINKNITSHPTSSAQKIDVLKPRPRSFSDKNSCNDVNILPKVDVVPLHLGLNNNYVCESSITCNKDEQKDKLININLLDGDNMWWESLLEADVLGPEATETAKGVTLPLDFEQIWARFDEETLELN

>AtMYB114_At1g66380

MEGSSKGLRKGAWTAEEDSLLRQCIGKYGEGKWHQVPLRAGLNRCRKSCRLRWLNYLKPSIKRGKFSSDEVDLLLRLHKLLGNRWSLIAGRLPGRTANDVKNYWNTHLSKKHEPCCKTKIKRINIITPPNTPAQKVDIF

>AtMYB115_____At5g40360

MCGYTAHNQGLIIGCHEPVLVHAVVESQQFNVPQSEDINLVSQSERVTEDKVMFKTDHKKKDIIGKGQWTPTEDELLVRMVKSKGTKNWTSIAKMFQGRVGKQCRERWHNHLRPNIKKNDWSEEEDQILIEVHKIVGNKWTEIAKRLPGRSENIVKNHWNATKRRLHSVRTKRSDAFSPRNNALENYIRSITINNNALMNREVDSITANSEIDSTRCENIVDEVMNLNLHATTSVYVPEQAVLTWGYDFTKCYEPMDDTWMLMNGWN

>AtMYB116____At1g25340

MSNITKKKCNGNEEGAEQRKGPWTLEEDTLLTNYISHNGEGRWNLLAKSSGKSCRLRWLNYLKPDIKRGNLTPQEQLLILELHSKWGNRWSKISKYLPGRTDNDIKNYWRTRVQKQARQLNIDSNSHKFIEVVRSFWFPRLINEIKDNSYTNNIKANAPDLLGPILRDSKDLGFNNMDCSTSMSEDLKKTSQFMDFSDLETTMSLEGSRGGSSQCVSEVYSSFPCLEEEYMVAVMGSSDISALHDCHVADSKYEDDVTQDLMWNMDDIWQFNEYAHFN

>AtMYB117____LOF1

MDEIVARRASSSWDFPFNDINIHQHHHRHCNTSHEFEILKSPLGDVAVHEEESNNNNPNFSNSESGKKETTDSGQSWSSSSSKPSVLGRGHWRPAEDVKLKELVSIYGPQNWNLIAEKLQGRSGKSCRLRWFNQLDPRINRRAFTEEEEERLMQAHRLYGNKWAMIARLFPGRTDNSVKNHWHVVMARKYREHSSAYRRRKLMSNNPLKPHLTNNHHPNPNPNYHSFISTNHYFAQPFPEFNLTHHLVNNAPITSDHNQLVLPFHCFQGYENNEPPMVVSMFGNQMMVGDNVGATSDALCNIPHIDPSNQEKPEPNDAMHWIGMDAVDEEVFEKAKQQPHFFDFLGLGTA

>AtMYB118_____At3g27780

MQPHTYEIPSKETIRGITPSPCTEAFEACFHGTSNDHVFFGMAYTTPPTIEPNVSHVSHDNTMWENDQNQGFIFGTESTLNQAMADSNQFNMPKPLLSANEDTIMNRRQNNQVMIKTEQIKKKNKRFQMRRICKPTKKASIIKGQWTPEEDKLLVQLVDLHGTKKWSQIAKMLQGRVGKQCRERWHNHLRPDIKKDGWTEEEDIILIKAHKEIGNRWAEIARKLPGRTENTIKNHWNATKRRQHSRRTKGKDEISLSLGSNTLQNYIRSVTYNDDPFMTANANANIGPRNMRGKGKNVMVAVSEYDEGECKYIVDGVNNLGLEDGRIKMPSLAAMSASGSASTSGSASGSGSGVTMEIDEPMTDSWMVMHGCDEVMMNEIALLEMIAHGRL

>AtMYB119____At5g58850

MEDRRLVHGAAPPLTAVERFLYGQKNDALCSKKQESSRDQPIVKTKISIETRSDNKENTTFGPTREKHLVLNGGNRNPTGEVVARSAARDYQNSTKKRSSKNLIKGQWTAEEDRKLIRLVRQHGERKWAMISEKLEGRAGKQCRERWHNHLRPDIKGSDISPSISTFLPKQKKNQKDGWSEEEERVLVESHMRIGNKWAEIAKLIPGRTENSIKNHWNATKRRQNSKRKHKRESNADNNDRDASPSAKRPCILQDYIKSIERNNINKDNDEKKNENTISVISTPNLDQIYSDGDSASSILGGPYDEELDYFQNIFANHPISLENLGLSQTSDEVTQSSSSGFMIKNPNPNLHDSVGIHHQEATITAPANTPHLASDIYLSYLLNGTTSSYSDTHFPSSSSSTSSTTVEHGGHNEFLEPQANSTSERREMDLIEMLSGSIQGSNICFPLV

>AtMYB120____At5g55020

MIMYGGGGAGKDGGSTNHLSDGGVILKKGPWTAAEDEILAAYVRENGEGNWNAVQKNTGLARCGKSCRLRWANHLRPNLKKGSFTGDEERLIIQLHAQLGNKWARMAAQLPGRTDNEIKNYWNTRLKRLLRQGLPLYPPDIIPNHQLHPHPHHQQQQQHNHHHHHHQQQQQHQQMYFQPQSSQRNTPSSSPLPSPTPANAKSSSSFTFHTTTANLLHPLSPHTPNTPSQLSSTPPPPPLSSPLCSPRNNQYPTLPLFALPRSQINNNNNGNFTFPRPPPLLQPPSSLFAKRYNNANTPLNCINRVSTAPFSPVSRDSYTSFLTLPYPSPTAQTATYHNTNNPYSSSPSFSLNPSSSSYPTSTSSPSFLHSHYTPSSTSFHTNPVYSMKQEQLPSNQIPQIDGFNNVNNFTDNERQNHNLNSSGAHRRSSSCSLLEDVFEEAEALASGGRGRPPKRRQLTASLPNHNNNTNNNDNFFSVSFGHYDSSDNLCSLQDLKSKEEESLQMNTMQEDIAKLLDWGSDSGEISNGQSSVVTDDNLVLDVHQLASLFPADSTAVVAATNDQHNKNNNNNCSWDDMQGIR

>AtMYB121_____At3g30210

MLDWGVQGHHQKHDHDIYQQQHQQQGCRKGPWTLEEDKLLAEYVTSHGEGRWSTVAKCAGLNRSGKSCRLRWVNYLRPGLKRGQITPQEEGIILELHSLWGNKWSTIARYLPGRTDNEIKNYWRTHYKKNQKSSSKQDKVKKSLSRKQQQVDLKPQPQAQSENHQSQLVSQDHMNIDNDHNIASSLYYPTSVFDDKLYMPQSVATTSSDHSMIDEGHLWGSLWNLDEDDPHSFGGGSGQGTAADIDEKFPDSGIEAPSCGSGDYSYTGVYMGGYIF

>AtMYB122____At1g74080

MVRTPCCRAEGLKKGAWTQEEDQKLIAYVQRHGEGGWRTLPDKAGLKRCGKSCRLRWANYLRPDIKRGEFSQDEEDSIINLHAIHGNKWSAIARKIPRRTDNEIKNHWNTHIKKCLVKKGIDPLTHKSLLDGAGKSSDHSAHPEKSSVHDDKDDQNSNNKKLSGSSSARFLNRVANRFGHRINHNVLSDIIGSNGLLTSHTTPTTSVSEGERSTSSSSTHTSSNLPINRSITVDATSLSSSTFSDSPDPCLYEEIVGDIEDMTRFSSRCLSHVLSHEDLLMSVESCLENTSFMREITMIFQEDKIETTSFNDSYVTPINEVDDSCEGIDNYFG

>AtMYB123____At5g35550_TT2

MGKRATTSVRREELNRGAWTDHEDKILRDYITTHGEGKWSTLPNQAGLKRCGKSCRLRWKNYLRPGIKRGNISSDEEELIIRLHNLLGNRWSLIAGRLPGRTDNEIKNHWNSNLRKRLPKTQTKQPKRIKHSTNNENNVCVIRTKAIRCSKTLLFSDLSLQKKSSTSPLPLKEQEMDQGGSSLMGDLEFDFDRIHSEFHFPDLMDFDGLDCGNVTSLVSSNEILGELVPAQGNLDLNRPFTSCHHRGDDEDWLRDFTC

>AtMYB124_____At1g14350_FLPs

MEDTKKKKKKNINNNQDSKKKERHIVTWSQEEDVILREQITLHGTENWAIIASKFKDKSTRQCRRRWYTYLNSDFKRGGWSPEEDMLLCEAQRVFGNRWTEIAKVVSGRTDNAVKNRFTTLCKKRAKHEAMTKDSNSNTKRMLFLDGISTPRKSENETPIAKKLKRSHILDLTEISNYGRAEACVNQQIRSPFSVLARNATGIDSLEEQNQTSNVNESDGEGMFLKKDDPKVTALMQQAELLSSLAQKVNADNTEQSMENAWKVLQDFLNKGKENDLFRYGIPDIDFKIEEFKDLIEDLRSGYEDNQLSWRQPDLHDSPASSEYSSGSTIMVDQSGDKTQPFSADTQTEHKQVGEELLVPKNPDENMPISGEEKFSSPIQVTPLFRSLADGIPSPQFSESVSFAPSLFIIQYLGFEKTMSLKRLIAFVGEELPAKNTRDRVLISMSKC

>AtMYBL2

MNKTRLRALSPPSGMQHRKRCRLRGRNYVRPEVKQRNFSKDEDDLILKLHALLGNRWSLIAGRLPGRTDNEVRIHWETYLKRKLVKMGIDPTNHRLHHHTNYISRRHLHSSHKEHETKIISDQSSSVSESCGVTILPIPSTNCSEDSTSTGRSHLPDLNIGLIPAVTSLPALCLQDSSESSTNGSTGQETLLLFR

>MdMYB2

MEMEVADDPAAVEIDAGDCGDCGEEAAAGGGAGSRRKSGRDNVKGPWSPEEDAVLSRLVSNFGARNWSLIARGIDGRSGKSCRLRWCNQLDPAVKRKPFTDAEDRMIVAAHAVHGNKWAAIARLLPGRTDNAIKNHWNSTLKRRCMDPEKIKLESGNMVEDVSLDKTKGSSEETFSYGDVNSSKSMEGREVSSFQHMDDQDEYNGSSEVQFSHEATEQPNLFRPVARISAFSVYNQSNSQEPESSFPRPIPAPGPLVRESKLDVGISKFLKGVYGELSVPDQCAHGCCCAQNGMNFQNSLLGPEFVEFSEPPSFPSFELAAIATDISNLAWLKSGLENNRVRGMGDAAGRITTHESQGQMGRFEDSRTNHLFPVEERNDRRMGMKTNVLST

>MdMYB3

MVQRELDRVKGPWSPDEDEKLRQIVQRYGARNWSVISKSVPGRSGKSCRLRWCNQLSPEVEHRAFTPEEDEIIAGAHAKYGNKWATIARLLNGRTDNAIKNHWNSTLKRKFSALSPNEDGATIDEGILRPEKKTATAAVSFSGLCYSPGSPSGSDVSESDVPASSSSQVFRPVARTAAIVTRESPPSQTEWCEPSTLLTLSLPGAESALHELPTNESAPHRGQRTASLQPEKETNVNNSNTMSFGPEFMSVMQEMIRKEVRSYMEGAACSQGEGVRNAGVKRIGISRLD

>MdMYB4

MAGAKLEECCLENKQSMATSSSSVSEGSGSAIIKSPGACSPASASPTQRRTSGPIRRAKGGWTPQEDETLRNAVAAFKGKSWKKIAEFFPDRSEVQCLHRWQKVLNPELVKGPWTQEEDDKIIELVAKYGPTKWSLIAKSLPGRIGKQCRERWHNHLNPDIKKEAWTLEEELALMKAHQMHGNKWAEIAKVLPGRTDNAIKNHWNSSLKKKLDFYLATGKLPPPPKYGAKEPRSNATKKILVRSTKGSDSTAQTSSGNADMSKLDEDGKDQFESAAPPQDMGASSSVHPNECADSEGVECDLRLSFMDLSCSNSDSGPKFDNFGVKRKPQIENSVSNGRAESENCGFSNQMDEDSVKTTSFPFETPRYGSLYYEPPPLESSVQFNSGFSSMHRLQNDYTSSPNLSPISFFTPPCVKGSGLCNQIPESILKIAAKTFPNTPSILRKRKTIGQGKSPCHKVAVVDCESVREMLNVSSEHKNSAEVSGSQDGSLCESPTSNGISTIGPNGKAFNASPPYRLRARRTAVFKSVEKQLEFTCDEEYDANSMSVELSANGGSVVIKDCTHPTKMAVT

>MdMYB5

MASTKKVVDRIKGPWSPEEDEALQNLVKNYGPRNWSLISKSIPGRSGKSCRLRWCNQLSPEVEHRPFSPEEDDTIIRAHARFGNKWATIARLLNGRTDNAIKNHWNSTLKRKSSSMSEDLSSDVQAHPPHKRSASVGAVPVTGLYFNPGSPSGSDLSDSSLPGGVSPSSQVFTPFARPLPPPIALPPMEAVTSAMAVDPPTSLSLSLPGSESCDGSSHMASGFGTNSIVGPAQIVQQAPEVTAPPRAVGLPPAPRQSNQNSETGHDQQFFSSEFLDMMQEMIRKEVRNYMTGIEQKGLCMHTEAIRNAVVKRIGISRIE

>MdMYB6

MDRIKGPWSPEEDDSLQRLVQKHGPRNWSLISKSIPGRSGKSCRLRWCNQLSPQVEHRAFTPEEDDMIIRAHARFGNKWATIARLLNGRTDNAIKNHWNSTLKRKCSDGGGVDLNGGYDGHFLRDHEQPPLKRSVSAGSGVPVSTGLYMSPGSPSGSDASDSSAQVMSLSDCHVYRPLARTGGVLPPAETTSSSNNNSSNSEKNDPPTSLSLSLPGVDSEEVSNQVAPVTESTQAPAPAPVSTTSVAVQNIPVELLAVMQGMIRKEVRNYMAGLEQSGVCFQQAAAGNEGFWNVGVKRIGFSRIE

>MdMYB7

MAAPTTPNEENEFRRGPWTLEEDNLLIHYIVNHGEGHWNSVAKLAGLKRTGKSCRLRWLNYLKPDIKRGNLTPQEQLMILELHSKWGNRWSKIAQHLPGRTGNEIKNYWRTRVQKQARQLNIESNSEQFLDAVRGFWVPTLLQKMEQSSSSCSSTLSTSQNSASPCLSPNHAAPSVPLSTSPPSNATNVLDNYHISGNSNLATVPSNILSADSFVSHVPQMAEPSTSFPPAYYRLGYSSLSPDGSHYVDSSSYDVEGLSLDPVSPMGNLGNSQFDCQMGGNDWMLDNVTDSLWNMDGP

>MdMYB8

MSTNTKTLSSNYSGEDDSELRRGQWILEEDSLLIQYIERHGEGQWNLLAKRSGLRRTGKSCRLRWLNYLKPDVKRGNLSPEEQLLILDLHSKMGNRWSKIARYLPGRTDNEIKNYWRTRVHKQARHLNIDTKSREFQNMIRCYWMPRLKQKIGRETSISSAVLNQNPTISQPRENNTFQHFTATISPPPQILVQEEINMSGTMYNLDVEKQNTEADYCRSSFIFPSEPMDMSKTAQFPECPPFYCGDNNGYDMESFNLQESVSADSAPNFPGNSAGDCYVAENNWFDSDFFM

>MdMYB9

MEKPSQKSCLKRCGKSCRLRWLNYLRPDIKRGNISGDEEELIVRLHNLLGNRWSLIAGRLPGRTDNEIKNYWNTTLGKKSKVDSFSGSSKETSLNPCKSIAKKKDVESKTSTAAAQPLVIRTKATRLTKILVPQNIPSDENYTAAAANPLELQTQSAEKGGSTEEFPRTNAGDCSNILKNFGCDDDDIDAKGDQYCNEFQLLNSIPLDEAMINDGCWTGGNGCDLEDYGASLDLDSLAFLLDSEEWPSQENVVV

>MdMYB10

MEGYNENLSVRKGAWTREEDNLLRQCVEIHGEGKWNQVSYKAGLNRCRKSCRLRWLNYLKPNIKRGDFKEDEVDLIIRLHRLLGNRWSLIARRLPGRTANAVKNYWNTRLRIDSRMKTVKNKSQEMRETNVIRPQPQKFNRSSYYLSSKEPILDHIQSAEDLSTPPQTSSSTKNGNDWWETLLEGEDTFERAAYPSIELEEELFTSFWFDDRLSPRSCANFPEGHSRSEFSFSTDLWNHSKEE

>MdMYB11

MGRSPCCSKDEGLNRGAWTAMEDKVLTEYIGNHGEGKWRNLPKRAGLKRCGKSCRLRWLNYLRPDIKRGNITRDEEELIIRLHKLLGNRWSLIAGRLPGRTDNEIKNYWNTTIGKRIQVEGRSCSDGNRRPTQEKPKPTLSPKPSTNISCTKVVRTKASRCTKVVLPHESQKFGYSTEQVVNAAPTLDQAVNNPMVGIDDPLLPMSFLDDENNNSCEFLVDFKMDENFLSDFLNVDFSVLYNNEGAGKAAAAATTEDTSNKLHGPDLRSSKAPIIESELDCWLVDN

>MdMYB12

MTTASPSSSKAGIAGGSKTPCCVKVGLKRGPWTPEEDELLANYIKKEGEGRWRTLPKQAGLLRCGKSCRLRWMNYLRPSVKRGQIAPDEEDLILRLHRLLGNRWSLIAGRIPGRTDNEIKNYWNTHLSKKLISQGIDPRTHKPLNPDHHSAAADADVGNTNKSAAAAASSKANNRFSNPNPSPPPSDRLVHQGADPSINGNDGNIAIDDHDLGTIVHSCANMTTSINNPDASSSAAAMGTLSLRTNNNSHAGVLLGGGGNEEDEDINCCADDVFSSFLNSLINEDPFSVQHQLQQQVLHNGNVSTHAAGAGSDHVPLISMTSASTMAPSTFGWDSAVLMSSAFNQNDHQRVTDQTEQ

>MdMYB13

MGRSPYCENAGLKKGPWKPEEDQKLLAYIEEYGHGSWRTLSAKTGLQRCGKSCRLRWTNYLRPDIKRGNFSLQEEQTIIKLHALLGNRWSAIRAHLPKRTDNEIKNHWNTHLKKRLTKMGIDPMTHKPKSHALCPSQLKDAANLSHMAQWENARLEAEARLVRKSNLHVSTPFQTQLGSSAAPARLVNKDLAPPPPPPPPLCLDVLTAWQGSFTNSTSMPSFQYNNGGACDETMGMRVEDHGFGKSNKFSELKEIIDAYNIGEVWKAENANVLLPNFMEDLSDLLFESSNELYGNSNNVVGISCYGDFEENNKIDSHWNNMVNLVNCSK

>MdMYB14

MGRTPCCDNKKDLKKGPWSPEEDELLLNYISKNHGHGSWRSLPKLAGLQRCGKSCRLRWTNYLKPDIKRGPFSKDEEKLIIQLHGMLGNRWATIASQLPGRTDNEIKNLWNTHLRKGLVSMGIDPQTHEPLSSNCLSNNASASAATRHMAQWESARLEAEARLSRDSSLFSRVPEKSESDHFLRLWNSEVGKSFRKLSTSDKFACQSPISQASSSTKCGSLSAVTTEVGNLTGSSTMAGNHNEDLIYRPFLINTEDVEYKPFQFNTEEVGYKPFQSITEEVEFKICQSNTEDVMAGSVSSCSNDLEDSSDTALQLLLDYPTNDDMSFLENNANDYATTPAELTPNSFICPL

>MdMYB15

MGRSPCCEKAHTNKGAWSKEEDQRLIDYIRQHGEGCWRSLPKAAGLLRCGKSCRLRWINYLRPDLKRGNFTQEEDELIIKLHSLLGNKWSLIAARLPGRTDNEIKNYWNTHVKRNLISRGLDPQTHRPLNQATTATSTAPASRLGLRNRPPPSSVLFDHKPVNNHSKIELLKHPKMEQDYYNYKIESEANCSTATGSGTTTEEDQQQQQQKYKCGDLNLDLSIGLEPFQSKPTRASSGNSAESRLQQIIVPPNSNNNHLFF

>MdMYB16

MGRSPCCEKAHTNKGAWTKEEDDRLIAYIRAHGEGCWRSLPKAAGLLRCGKSCRLRWINYLRPDLKRGNFTEEEDELIIKLHSLLGNKWSLIAGRLPGRTDNEIKNYWNTHIRRKLLTRGIDPTTHRPLNETPQESATTISFAAASANIKEEDKKISITNGLVCKDSKNPVQERCPDLNLDLQISPPCQPQQPSDGLKSGGRGLCFSCSLGLQDAKNCSCGRDAIGGATSGTTNIGYDFLGLKNGVLDYRSLEMK

>MdMYB17

MRKPCCEKKKTNKGAWSKQEDEKLTEFVEKNGEGSWRSLPLAAGLLRCGKSCRLRWVNYLRPNLKRGNFGEDEEDLIIKLHALLGNRWSLIAGRLPGRTDNEVKNYWNTHLRRKLIQMGVDPNNHRIGHTQNIGLSKSSFGSRKANHPCKAANSQGDNDSDDHQKPFTDSASGPESNTSCSGLPDLNLDLTIGLPS

>MdMYB18

MGRAPCCDKNGLKKGPWTTEEDAMLVNYIQKHGPGNWRNLPKNAGLQRCGKSCRLRWTNYLRPDIKRGRFSFEEEETIIQLHSILGNKWSAIAARLPGRTDNEIKNYWNTHIRKRLLRMGIDPVTHAPRIDLLDLSSILSSYVCNNPAAALLNLSNLLNSTHQRQPLVNPEMLRLATSLLSIKQANPEMCSQNYNLHQNQISNSQEQNNQVLPPLQSNDQFQNLIQGGDFSADMAKHLMQQINVEGFSPNMTNLSCPLSQENIVPPNLSADHHQTAVSQANYVPCSTTSGNPGPDFPENSYFQSFNYNKNHDFSFDSVMSTPYSSPTPLNSSGTYINSSTEDEKESYCSSWLKFEIPESTLDISDIM

>MdMYB19

MGRQPCCDKLGVKKGPWTAEEDKKLVNFLLTHGQCCWRAVPKLAGLRRCGKSCRLRWINYLRPDLKRGLLNDAEEQLVIDLHARLGNRWSKIAARLPGRTDNEIKNHWNTHIKKKLVKMGIDPITHEPLHKQVTTPQEMPCEASNQPANSDMSIQQMNTNIPEHGISTNSDGNSTSENSPSNDSEPAEPNPNYSEEEDPLVSFILSDTFLEDLTWDFSTSSEYSSADNPTEENSLAWFMDCNDFGVEDFEL

>MdMYB20

MRKPEPSSAAAGKNNKENNSNSKLRKGLWSPEEDDKLMRYMINNGQGCWSDVARNAGLQRCGKSCRLRWINYLRPDLKRGAFLPQEEELIIHLHSLLGNRWSQIAARLPGRTDNEIKNFWNSTIKKRLKNLSSSNGSPNTSDSSPEAKDHRVVAASRFIIPGQEHGMVPIYMDSTSSFMQSAVLSHMFDPFPALDIDQGGLTLPGAGGYYNANPCITQREIGVGGGDDCYNFGGNGGFGSGDVDIGVEGEIFVPPLESVSIEDQNIKTETTYGDSKNNNIYYNNINSILTCNKTNKNIKGESIIGVGNYFDDDQEELTMGDWDLEDLMKDVSSSSFPFLDYQS

>MdMYB21

MAKRTQRCEKTVAVKKGPWSPEEDHKLVAYIQRYGIWNWSRMPKPAGLARSGKSCRLRWVNYLRPDIKRGNFGKDEEETILELHQAIGNRWSAIAARLPGRTDNEIKNYWHTHMKKRFKAGIESVHEEAKGSGDKEASKNKLSEAGQLFLVDEATKVASLKSEATCSASSSVYFPVWEFDDQKGQIAEQSVCASDVTFGELQSLDSQVWQQEPIFPCDSYINHVTSDYWVSLIDKTG

>MdMYB22

MGRAPCCEKVGLKKGRWTAEEDEILLNYIQANGEGSWRSLPKNAGLLRCGKSCRLRWINYLRADLKRGNISSQEEDIIIKLHASLGNRWSLIASQLPGRTDNEIKNYWNSHLSRKIGTFRRPATTTVITTEISTSVPPAGDEVSAAMELGPPKRRGGRTSRWAMKKNKTYSTTKPKGLKARLRQSHHKHDNIAAAAADDDRLNNAHEAIALPTKSNNKNVDTMQDGYVLLMEVPDQQQETRGGGIAMPATVIDHQKETDGEKLILGPHPHGHDDDDMNEYVGINGGLLGFTDYLMDDINEDEILDPNGVMALSSSEINIHQDADHFPDAVISDHQDTELPSSCDQLVICPNKVMTTTTTYYGIQ

>MdMYB23

MGRAPCCEKMGLKKGPWTSEEDQILTSFIHKYGHGNWRALPKQAGLLRCGKSCRLRWINYLRPDIKRGNFTREEEEAIIKLHEMLGNRWSAIAARLPGRTDNEIKNVWHTHLKKRLKDHTTTTSSTTSTRSSSNSTSNVKSQFVDEPENLNYPQPSSSDVSSSVTKVSAALSTDQDTIVVKGENMELSETFPDIDDDSFWSEALSTDNSSVPLQFPKASNDEPISEFPITKNDSEEFGFSFGLNMDDGMEFWYDLFIRTGHGGTPELPEF

>MdMYB24

MGRAPCCDKANVKKGPWSPEEDSKLKEYIEKYGTGGNWIALPQKAGLKRCGKSCRLRWLNYLRPNIKHGEFSDEEDRIICSLFASIGSRWSVIAAQLPGRTDNDIKNYWNTKLKKKLMGMHMGPPRPHNHHKNLLKPPPFPSSSHHNYQNQPLIPSEPLSSLYKDLSNYNRSFLGFEAPVPLPPQVSLTSNNFSNISTNSSIFQTLNYPAGVKENNNNNTLLVFGSEGSCSTSSDGSCNNQISYDYCSRSEINNINQEEMGFDHQGFMMLNYGNQWTERPNGFYFGSENNTLEFTDLDEDVKQQLISTRSKNNYNNNTIINESSKSNSFLFNVNESKTEDDEKVMYFY

>MdMYB25

MVRTPCRDENGMKKGTWTPEEDRKLIVYVTRYGCWNWRQLPKFAGLSRCGKSCRLRWMNYLRPNIKRGNYSKEEEETIIKLHEKLGNRWSAIAAQLPGRTDNEIKNHWHTNLKKRTNKKQCNSSFSSSATNTEETPSSSNLEAAVDQPIKKAIFPNAESTTALQVIKKSDDRVDNSSQLSSSPQPSSSEVSSMSADDNWVNFVEDFNLSSVEAYADNQFIDDFWTKPFLADNSYIPSGFYTPLMDSEFLYPLF

>MdMYB26

MGRAPCCSKVGLHRGPWTPREDTLLTKYIEAHGEGPLEILAKKSWPPQVWEELQAKVDELSKTRHKERQHNPPXEDDLIIRLHSLLGNRWSLIAGRLPGRTDNEIKNYWNTHLSKRLRNEGTDPNTHKKLSEPIARENKRRKNQRSKSNNNKKEMVMTKDKNNKTAQHVEPQKPKVHLPKPTRFTSFLSLPRNDSFTSSTTVTTGSSSQDLNGGGGRGGGGGGFGVNTWCNN

>MdMYB27B

MRKPGCDKIETNKGAWSKQEDQKLIDYIQKHGEGRWNSLPKAAGLRRCGKSCRLRWINYLRPDLKRGSIGEDEEDLIIRLHALLGNRWSLIAGRLPGRTDNEVKNYWNSHIRKKLSKMGSTLDPKKPHDHHDPHLRRGTTAGVPVLQPDARKPILFASSSSDSMSTGAKIHSNESGLPDLNLDLSL*

>MdMYB28

MRKPCCDKQDTNKGAWSKQEDLKLIEYIRKNGEGCWRTLPQAAGLLRCGKSCRLRWINYLRPDLKRGNFAEDEEDLIIKLHALLGNRWSLIAGRLPGRTDNEVKNYWNSHLRRKLMNMGIDPNNHRPNNVNLPRLHHQNSQTVSSTATLSEALKNPTNYQQQARSGGNNNCDHESDGTSCLEDDSCGRLPDLNLDLTITAPWSNPVPDNLKEEQNFESKVSKKSSEFASSTILPLFR

>MdMYB29

MGRSPCCDKVGLKKGPWTPEEDQKLLAYIEEHGHGSWRALPTKAGLQRCGKSCRLRWTNYLRPDIKRGKFSLQEEQTIIQLHALLGNRWSAIATHLPKRTDNEIKNYWNTHLKKRLAKMGIDPVTHKPKKDNLLSSVDGQSKNASNLSHMAQWESARLEAEARLVRESRLRSQSSSLLHQLTTTNPNTYVPVNSSSSGSTSAQLQLKWPSSKXILIIXVDLEXSDIXRSLIIPIRLTLIMHRPPAMGAAIMQPAMIEFVGSSGSSETKEEGGDNEQDWKSHLSFTPLGLVHDHHQNISMSMEAAGAWTTNDHQVGNVAGDMEAADQEGFTNLLLNNSDEVQSLSDGDRGVDFDNGGGSGSGRGSDYYEDNKHYW

>MdMYB30

MGRPPCCDKEGVKKGPWTPEEDIILVSYIQERGPGNWRAVPTNTGLHRCSKSCRLRWTNYLRPGIKRGNFTDQEEKMIIHLQALLGNRWAAIASYLPQRTDNDIKNYWNTHLRKKLSKLQAAGGGTEGLHSKDQGLNSNSSSQPISRGQWERRLQTDIHMARQALRDALSPEKPLTLSSDLNPTDGFSISSPKKPSLDQSSSSTYASSTENISRLLKGWMKNPPKKSASFTNLANNKTDYQHYSSSEGTTSVANTANSGNVELSDTFESLFGFESSNSYLSPSMSPEASLFQGESKPDLISDKLPLSFLEKWLFDESASPAALGKDHFFSDMLPDHGNANFF

>MdMYB31

MGRPPCCDKVGVKKGPWTPEEDIILVSYIQEHGPGNWRSVPTNTGLLRCSKSCRLRWTNYLRPGIKRGNFTDHEEKMIIHLQALLGNRWAAIASYLPQRTDNDIKNYWNTHLKKKLKKLQTGLDGHDHHNHQDGNSHDQPISKGQWERRLQTDIHMAKQALCEALSIDKPNVISTTTVSHDLHLQDLKPSINIGNYNINDNPSTSTRPHHTSTYASNTENIAKLLENWMKNSPKVSAHQTNSDQTNADQTNSFNNNNNNDNDNKGGATATTRGRSMSTSSEGAHSATTTPDQAFDSLFSFNSSPSDVSQSMSVDENNANFIADQASCLFQDESKPNLEGQVPLSLLEKWLLDDAAPHAHEDLINISLENGAGLF

>MdMYB32

MKGSDYISSDHDKNPNESGGKVSEKETESGQSKLCARGHWRPAEDSKLKELVALYGPQNWNLIAEKLEGRSGKSCRLRWFNQLDPRINRRAFTEEEEERLMQAHRIYGNKWAMIARLFPGRTDNAVKNHWHVIMARKYREQSSAYRRRKLNQTVYRSMEENPSFNAASRSTEPPPPYNGLSLPNGGLGGSGSISSFSYGTSYDGVVGYGADYYGSNGSQNLISVEEEAIARKKLLSYNNSGFCSQQTPEFFRGRKSNEVMGMMSQNQCWDGSGEMEGHSYGGMYTHHPPPNMNVMTMQQSNFHSLVHQKYSDSSCQFSGGKPSPSASAADRVVGSGGGSSRRETSIQPPPFIDFLGVGAT

>MdMYB33

MDRIKGPWSPEEDDSLQKLVQKHGPRNWSLISKSIPGRSGKSCRLRWCNQLSPQVEHRAFTPEEDDTIIRAHARFGNKWATIARLLNGRTDNAIKNHWNSTLKRKCSDVGGVVLNGGYDGHYLLDHEQPPLKRSVSAGSGVPVSTGLYMSPGSPSGSDVSDSSVQVMSLPDCHVYRPVARSGGVLPPVETTSSSNNSSNGEKENDPPTSLSLSLPGVDAGEVSNRVAAATESIPAPAPVPVPIPAAPVLNMPSELLGVMQGMIRKEVRSYMARLEQGGVCFQQAAAAGNDGFRNVGMNRIGYSRIE

>MdMYB34

MASTEKVVDRIKGPWSPEEDEALQNLVKIYGPRNWSLISKSIPGRSGKSCRLRWCNQLSPEVEHRPFSPEEDDTIIRAHARFGNKWATIARLLNGRTDNAIKNHWNSTLKRKCSSMSEDLCSDVHDHPPHKRSASVGAVSGTGLYFNPGSPSGSDLSDSSLHGGVSPSSQVFTPLARPVPPPIAPPPMEAATSAVAVDPPTSLSLSLPGSESESCDGSNHMGSGFGSNPIVGPTQMVEQPHEVAAPPPVVGLLPPPRQSNLNNNQQFFSSEFLDVMQEMIRKEVRNYMTGIEQKGQCMQTEAIRNAVIKRIAISKIE

>MdMYB35

METEVADDPAAVDIEAGYGGVDSGEEAVAGGGSRRKSGRDNVKGPWSPEEDAVLTWLVSNFGARNWSLIARGIDGRSGKSCRLRWCNQLDPAVKRKPFTHEEDRMIVAAHAIHGNKWAAIARLLPGRTDNAIKNHWNSTLKRRCMDTDKIKLESGNMVEDVSFDKTKGSSEETSYGDVNSSKSMEGREVSSLEHMDDQDEYNGSSEVQFSHEATEQPTLFRPMARISAFSVYNQSNSQEPASSFPRPIPVQGPLVQASKLDVGINKFLEGVYGEQSVPDRCGHGCCRAESGMNFQNSLLGPEFVEFSEPPLFPSFELAAIATDISNLAWLKSGLENNIVRAVGETAGRITTHGSQDQTGRFEESRMNHLFPTEERKDRLMGMKTNVLST

>MdMYB36

MGRAPCCDKNGLKKGPWTPEEDQKLMDYIQKHGYGNWRTLPKNAGLQRCGKSCRLRWTNYLRPDIKRGRFSFEEEETIIQLHSILGNKWSAIAARLPGRTDNEIKNYWNTHIRKRLLRMGIDPVTHSPRLDLLDFSSILYNSSHHHHQMNNFSRLLGQPIGLNPELLRLATSLIQSRRENNSNQNFVLQNAQENDYHQICNPQIQPQQPVQDNVPYPNEVSQLMQQQPNVEYPSSLSDFRSQNSQLNEWQSNVGTSNFTEEYVELPSFAYFGSEQHQTVLDLSPENSNFHSNNSNQNFSFTSVFSTPSSSPTTLNSNSTTYFNSGTEDERESYCSNMLKFGIPDILGVNEFM

>MdMYB37

MGRSPCCAKEGMNRGAWTAHEDKVLTEYIKLHGEGRWRNLPKNAGLNRCGKSCRLRWLNYLRPDIKRGNISPDEEELIIRLHKLLGNRWSLIAGRLPGRTDNEIKNYWNTNLGKKVHDHQQQGSASDLKHHKNGEPNSKKAKMMDMATPSSHVVRTKAAKCTKVFINPHPHKVPLGHNHQHYTEETNAGGLMFDDKPAAMDDDHINRTRSFSSFSNINADQENSTSDFLVDFDMDEISLANLLNSDFPEINHDDLNHISSDNVMSRCVDETAQFFSEGMLQHWKNGDSDNGDDRVQVQPNFILNFHSFNSFLGSDHQGEECLGVGKSS

>MdMYB38

MGRAPCCDKANVKKGPWSPEEDAKLKEYIEKYGTGGNWIALPQKAGLRRCGKSCRLRWLNYLRPNIKHGEFSDEEDRIICNLFTNIGSRWSIIAAQLPGRTDNDIKNYWNTKQXKEAHGHKHSPIPAAKISPISSPSNFIHILFAVIIPRKQHQHYILHTNQVFHRHFGAHFFFTKSNEQQFH

>MdMYB39

MDKNPFNSSSQDVEVRKGPWTMEEDLILINYIANHGEGVWNSLAKAAGLKRTGKSCRLRWLNYLRPDVRRGNITPEEQLLIMELHAKWGNRWSKIAKHLPGRTDNEIKNYWRTRIQKHIKQAENITPGQSSEVNDQASTSQVSISNTVDTMDISHSAPTHQANMDAYPPPLPADQSNDNYWSMEDLWSMQLLNGE

>MdMYB40

MGRHSCCYKQKLRKGLWSPEEDEKLLNYITKHGHGCWSSVPKLAGLQRCGKSCRLRWINYLRPDLKRGPFSQQEENLIIELHAVLGNRWSQIAAQLPGRTDNEIKNLWNSCIKKKLRLRGIDPNTHKPLSEVLDQNIGKEINNNNNIKLSPTYKSNEKASVGSNELSLVEAVSSRHPTSASKNRFNPVEVSSTSKLISSNGGSSKSLTHEGSPSSCRPCDFVGYFSFPHHNNQSNYGSSSDMGLQAVNQNSTFSFLNQNPEFLQPSMSSSSAIFTAPPPPPTRVKPSISLPSDNSSTWDSNANNSNSSGGGYFDSSAAFSWWPPEAVKSDDPEEIKWSEYLHNTSFLMGTHQASQPATYSEIKPESQSHILSNSLSATSWHHQNHQQQQQQALQASEMYTKDLQRLAVSFGQTL

>MdMYB41

MGRAPCCEKVGVKRGRWTAEEDEILTNHIKAHGEGSWRSLPKNAXIAEVWEELQIEVDKLSEADLKRGNITSEEEEIIVKLHTALGNRWSLIAARLPGRTDNEIKNYWNSHLSRKIYSFTKMGNEYLPTILNDEKKIASCNKRRRGAQASRSTMNNKSKNKNLLAPFNPIKSEAKPQLEKPSPPTGTTINRGQVGDEDSKLMG

>MdMYB42

MGRKPCSDKEGLNRGAWLAWEDKILTNYIETNGEGKWRDLPRRAGLNRCGKSCRLRWLNYLRPDIKRGNISSEEEELIIRLHKLLGNRWSLIAGRLPGRTDNEIKNYWNTTLSKKTEQGSDKAPHHELTSTTNSKEYTPKPKPASKSHQTVQVIRTKAFKCRKVVIPSHLDGHDQMIDRLNVNPGLFPCESPSSSASQDPQEDSPPCGFLKIFDIDDLFISDLLNTDNFLQX

>MdMYB43

MSRTTNESEDGMLYKDQIDSPLMDESNGGSGNGGIVLKKGPWTSAEDTILVEYVNRHGEGNWNAVQKHSGLFRCGKSCRLRWANHLRPNLKKGAFTPDEERLIVELHAKMGNKWARMAAHVSTWFIFVCFYMVHTKLYFLVLPQTSILYGSFFWDFLSKL

>MdMYB44

GRQPCCDKLGVKKGPWTAEEDKKLVNFLLTMANVVGGLSPSSLASVAAARAAASGGLITSAXDLKRGLLNDAEEQLVIDLHARLGNRWSKIAARLPGRTDNEIKNHWNTHIKKKLIKMGIDPITHEPLHKQAITPEEMPCEASNQPAGSDVNIPQNMDIPEHEVYSNSEASNQPAGLDMNIPRNMNIPEHEISSNSDGGGSASENSPSNESQSAEPNPNYSEEDDPLLSFILSDTFLEDLTWDFSTSSEDGSADNPTEENSLAWFMDCKDFGVQDFEL

>MdMYB45

MGRSPCCSKDEGLNRGAWTAMEDKVLTEYIRIHGEGKWRNLPKRAGLKRCGKSCRLRWLNYLRPDIKRGNITHDEEELIIRLHKLLGNRWSLIAGRLPGRTDNEIKNYWNTTIGKRIQVEGRSCSDGNRRRPTQEKTKKPTQSSPEPSTNNSSPKWSEPKRQGAQKWSYPMSAYKCGDSTEQVIDAAPIFX

>MdMYB46

MGRQPCCDKVGLKKGPWTAEEDKKLIKFILANGQCCWRAVPKLAGLLRCGKSCRLRWTNYLRPDLKRGLLSEYEEKMVIDLHAQLGNRWSKIASHLPGRTDNEIKNHWNTHIKKKLRKMGIDPLTHKPIANVMIKANNHKVKNKEEKKNKSCAANDSFEIGQNTPNSSQRRX

>MdMYB47

MTAPNGAVPKQADDRPGTEAELNEGAVPNGKVRGPWSPEEDAVLSRLVSNFGARNWSLIARGIPGRSGKSCRLRWCNQLDPCVKRKPFSEEEDRIIVSAHAIHGNKWAVIAKLLPGRTDNGIKNHWNSTLRRKCFDKGRFNTGPGEMMEDDTFDRKNASSEETLSVGNISSFKTHEGREVLMENRPNQFDVRSHAKEGSGAAESKHNSTLIAEPRDHPTLQSTICCPVARVSAFSVYNRPSVPANASSLSRTVPSHGPLVPITKLDFGFDNFLEGACNEPTVPQRCGHGCCDRIEGHSQSSLLGPEFVEYDEPLPFSSHELISIATDLNKIAWIKGGLESNGIRMPEHVASQRVFQAAATTLQMGLPANTPMNDHMRFEEGRNKLMGMMTDVLSTQVPRQTFAMPTEVEGLS

>MdMYB88

MPQEESKKKERHIVTWSQEEDDILRNQISTHGTENWAIIASKFKDKTTRQCRRRWYTYLNSDFKKGGWSPEEDMLLCEAQKIFGNRWTEIAKVVSGRTDNAVKNRFSTLCKKRAKYEALAKENAASHINQNDKRVIIRNGFNTDGTAETTAPSKKMRRSHIPSLSEGDRLLEQCGKMSQQLRAPFAVLIQNVHNVENLPDQNNVNGTKEVPVNAVQKSKFHGSFLKKDDPKIIALMQQAELLSSLALKVNAENTDQSLENAWKVLQDFLNQSKDSDILSYGINDFDFQLEDLKYLLEDLRSTTEGSRPSWQHCRQPDLYEESPGSSEYSTGSTLLSQTEYYQVEKNEVEIGSLNQEIRPGSQSIPIEGKNGVGDCEKGIFSEKQEIFPSCDEATKDYAVVSALSSIEFNSPIKVTPLFRSLAAGIPSPKFSESERNFLLKTLGEDSPCPNPSTNPSQPPPCKRSLLQSL

>MdMYB89

NKPWSFQALENGGPKNISDLGVLSGVDQKRPTPLNLNLVGEEDDDEGRSPAGGRTSAKSCIRGHWRPAEDSKLKELVGQYGPQNWNVIAEHLDGRSGKSCRLRWFNQLDPRINRGAFSEEEEERLLAAHRLYGNKWAMIARLFPGRTDNAVKNHWHVIMARRHREQSNVFKRRKPSSPPPPSPLPHVVANFPKNPSTSTESTVSISTINDDSTQSASTCTDLSLTPSNKPPPTFFSKFHPFHRPQIIGSPMGPSSSDGGFNKMIGSNGNGFCKSTGPVRGVGAAAAAMKGVDNSGHQSDSEISGDESVTTTRTNLSLSGEDQDLHHEKTTTIHFIDFLGVGTA

>MdMYB90

MGKSSCYAAASGIKRGPWTPEEDRKLLAYIQLHGHGSWRSLPQKAGLKRCGKSCRLRWRNYLRPDIKRGNFSLHEDQTIIQLHALLGNRWSAIAAHLPRRTDNEVKNYWNTHLKKRLAKIGFDPVTHKPKAAILGSANGDPKNWSNLSHIAQWESARLQAEARFVKESKLRMLESAPAPSLSDEHHQFAPPLVPQCLDILQASAWESX

>MdMYB91

MKERQRWSAEEDALLRAYVKQYGPREWNLVSQRMNTPLDRDAKSCLERWKNYLKPGIKKGSLTEEEQRLVICLQAKHGNKWKKIAAEVPGRTAKRLGKWWEVFKEKQQREQKNKKITDPIVEGKYDTILETFAEKLVKERAPTYLMATSNGAYLHTETSSPAPTILPPWLSNSNVSPNVRPPSPSVTLSLSPTVAPSPPIPWLQQDRGSDGSFVVGNLPHHGVVPACGENLVISELVECSRELEEMHRAWAAHKKEASWRLRRVELQLDSEKPCRRREKMEEIEAKVKALREEQKAALDRIEAEYRDQLAGLRRDAEAKEQKLAEQWAAKHLRLSQFLEQMGGRPRIVEPNGR

>MdMYB92

MKERQRWQPEEDALLRAYVKQYGPKEWSLVSQRMGKPLLRDPKSCLERWKNYLKPGLKKGSLTPEEQALVVSLQAKHGNKWKKIAAELPGRTPKRLGKWWEVFKEKQLKLQSQRQKNKHLLCSTSSSSTQLPPDMNIPVAGIGSPEKAVKGPYDHILETFAEKYVVQPKVYGAAFQSTTMMQEPDPVLSLGSVGSTATSVTAPSVMPQWMNINPSSTTSSTSSTTPSPSVSLTLSPSDPVPDTDPTRFYQMGTLIQLCKEVEEGMQSWMQQKKEATWRLSRLEQQLEAEKGRKRREAVEEIEAQIRCLRQEEVALVGRIERDYREELSALQREAEGKEAKFVEAWCGKHAKLAKLVERIAVGVRNQGFSSKPS

>MdMYB93

MKERQRWSAEEDALLRAYVKQYGPREWNLVSQRMNTPLDRDAKSCLERWKNYLKPGIKKGSLTEEEQRLVICLQAKHGNKWKKIAAEVPGRTAKRLGKWWEVFKEKPPREPKTRKIPDPIVEGKYDTILETFAEKLVKERAPTYLMATSNGAYLHTETSSPAPTILPPWLSNSNVSPNVRPPSPSVTLSLSPTVAPSPPIPWLQQDRGSDGSFVVGNLPHHGVVPACGENLVISELVECSRELEEMHRAWAAHKKEASWRLRRVELQLDSEKACRRREKMEEIEAKVKALREEQKAALDRIEAEYREQLAGLRRDAEAKEQKLAEQWAAKHLRLSQFLEQMGGRPRIVEPNGR

>MdMYB94

SNLPFLRFDLQICSVAIASSILRLREISVLRSSKLSWVAGLVFNKVGPTIALGDYNAYESKYWRVSLCRNPVMQLDLNSTPSEELCDSIQNVQVLHGRTSGPIRRSTKGQWTPEEDEILRRAVERFKGKNWKKIAECFNGRTDTQCLHRWQKVLNPRMVKGPWSKEEDDIIIEWVEKYGPKDWCSIAQHLPGRIGKQCRERWHNHLNPAIKKDAWTQAEEFALIRAHQIYGNQWAEITRFLPGRQCHKKSLEQFSEEEVGFCEDEVLSILEVGEGFPRELVESHALDFCVLPEKKDTENVKSSSSAKKLLKSDILIVEGVQKTGFEKVVLVLVFEPHETDDNSGGRRNLELHKSYRVIGTNLFWDQKFVQYSRDYIRDY

>MdMYB100

MRIMIKGGVWKNTEDEILKAAVMKYGKNQWARISSLLVRKSAKQCKARWYEWLDPSIKKTEWTREEDEKLLHLAKLMPTQWRTIAPIVGRTPSQCLERYEKLLDAACVKDENYEPGDDPRKLRPGEIDPNPESKPARPDPVDMDEDEKEMLSEARARLANTRGKKAKRKAREKQLEEARRLASLQKRRELKAAGIDTRQRKRKRKGIDYNAEIPFEKKPPPGFYDVADEDRPVEQPNYPTTIEELEGKRRIDVEAQSRKQDIAKNKIAQRQDAPSAILQANKMNDPETVRKRSKLMLPAPQISDNELEEIAKMGYANDLAVEEFAEGSGATRALLANYSQTPRLGMTPSRTPQRTPAGKGDAIMMEAENLARMRESQTPLLGGDNPELHPSDFSGVTPRKKEIQTPNLMLTPSATPGGGLTPRTGLTPRVGMTPSRDSFGMTPKGTPIRDELRINEEIDTPNSAKLEQRRNLQFGFGSLPQPKNEYQIVVQPVPEDNEEPEEKIEEDMSDRLARERAEEEARQQALLRKRSKVLQRELPRPPSASLDLIRNSLIKADGDRSSFVPPTPIEQADEMVKVELLSLLEHDNAKYPFTEKVDKRKKKGSKRSANGPTTSVPVIEDFEEDELKEADSLIKEDAEILRVAMRHENETLDEFVEAHKTCLNDLMYFPTRGAYGLSSVAGNMEKLAALQSEFENVKKKMDDDIQKAASIENKVKVRTYGYEMRAKEGLWPKIEETFKQIDTAAKELECFKALQKQERLAATHRINNIWEEVQNQKELERQLQKRYGNLVAEVERGQQRMDELRAEAEKQEIAARNSDLELAEATANVTVQQTTENPDHVTASDEPGNAVPGGASHVEGTNQQMDGVQEQASTGPEGDMDNDAKKVHATPADDVNLPDNMPSAVEGDNVSDSVVASENLKVNDPDVCKDQKVEMPQNVTEIEGTVQAAGDDGFANGTAAIVSVEGEVNSTENAAPQI

>MdMYB110a

MEGYNVNLSVMRKGAWTREEDDLLRQCIEILGEGKWHQVPYKAGLNRCRKSCRLRWLNYLKPNIKRGDFTEDEVDLIIRLHKLLGNRWSLIAGRLPGRTANDVKNYWNTRLRINSRMKTLQNNSQETRKTIVIRPQPRSFIKSSNYLSSKEPIIDHIQSEEDLSTSPQTSSSTNNGNDWWKTLLEDDDILLKELYVPVLS

>MdMYB111

MRKPCCEKEGTNKGAWSKQEDQKLIDYIKTHGEGCWRSLPKAAGLHRCGKSCRLRWINYLRPDIKRGNFEQDEEELIIKLHALLGNRWSLIAGRLPGRTDNEVKNYWNSHIRKKLIKMGIDPNNHRLNQIIPRPNPQNDSVSPAATSSGSMSNINACTKTPLKSSDDQIDHRASEAASVLEDETSGPSSRDLNLDLTIAFPEPSLQVEEGMPKLIKGSNTTAREIETNLQHLPTLVLFR

>MdMYB114

WTKEEDDLLQHFVQQHGEGKWRQVPLKAGLNRCRKSCRLRWLNYLKPSIKRGDFGEDEIDLMVRLHKLVGNRWSMIAGRLPGRTANDVKNYWSTRLRWKKSASGDKLKNHKPLQQVAKVETTKTVVIRPRPRTFSKNLNYLSRKLTATNSNIQQHNNQVLLQEPLAVTSSLTPX

>MdMYB115

MKLDSSESSEECKGSGGEMGGIPWTKEEDQLLRKCIEQHGEGKWHRLPLLAGLNRCRKSCRLRWLNYLRPNIKRGNFEEEEVDLIIKLHRLLGNRWSSVAARLPGRTGNDVKNYWNCHLSKRLNAQQDHQSEYHIKNKHDAVRNINNGVKVXXDPKLQTLQVQAAQRGRHMHQXPHQPVNVFVQEEGSSTGPKPKLCNFNGNGQNNNQIFENNNNNNXXTGINASDYPMDDDPSPFDQQV
