# Supplementary material for: Multiple Copies of a Simple MYB-Binding Site Confers Trans-regulation by Specific Flavonoid-Related R2R3 MYBs in Diverse Species
Source: Front Plant Sci. 2017 Oct 31;8:1864. doi: 10.3389/fpls.2017.01864 (PMC5671642; doi:10.3389/fpls.2017.01864)
Supplement: Supplementary file 4 [file Data_Sheet_2.docx]

**Supplementary Data Sheet 2:** Detailed conditions for the HPLC and LC-MS analysis

**Analysis of samples by UHPLC**

The UHPLC system was composed of a Dionex Ultimate® 3000 Rapid Separation LC system (Thermo Scientifc). Instrument control and data analysis was performed using Chromeleon v7.2 (Thermo Scientific). The separation column was a Zorbax^TM^ SB-C18 2.1 x 100 mm, 1.8 µm (Agilent, Melbourne, Australia) and were maintained at 35 °C. The flow was 400 µL/min. The solvents were A = 5% formic acid, and B = 100% acetronitrile. The solvent gradient was: 95% A, 5% B, 0-0.5 min; linear gradient to 60% A, 40% B, 0.5-10 min; linear gradient to 100% B, 10-15 min; composition held at 100% B, 15-17 min; linear gradient to 95% A, 5% B, 17-17.2 min; to return to the initial conditions before another sample injection at 20 min. The injection volume for samples and standards was 1 μL. Compound concentrations were calculated using external calibration curves generated from authentic standards.

**Analysis of Arabidopsis samples by LC-HRAM-MS**

The liquid chromatography - high resolution accurate mass – mass spectrometry (LC-HRAM-MS) system was composed of a Dionex Ultimate® 3000 Rapid Separation LC and a micrOTOF QII high resolution mass spectrometer (Bruker Daltonics, Bremen, Germany) fitted with an electrospray ion source. The LC separations used a Triart C18 2.0 x 150 mm, 1.9 µm column (Agilent, Melbourne, Australia) and was maintained at 60 °C. The flow was 350 µL/min. The solvents were A = 1.0% formic acid and B = 100% acetronitrile. The solvent gradient was 10% A, 90% B, 0-0.5 min; linear gradient to 100% A, 0.5-25 min; composition held at 100% A, 25-28 min; linear gradient to 10% A, 90% B, 28-28.2 min; to return to the initial conditions before another sample injection at 31 min. The injection volume for samples and standards was 1 μL. The micrOTOF QII parameters for polyphenolic analysis were: temperature 225 ºC; drying N_2_ flow 6 L/min; nebulizer N_2_ 1.5 bar, endplate offset -500V, mass range 100-1500 Da, acquired were acquired at 2 scans/s. Negative ion electrospray was used with a capillary voltage of +3500 V. Post-acquisition internal mass calibration used sodium formate clusters with the sodium formate delivered by a syringe pump at the start of each chromatographic analysis. Polyphenols compounds were quantified using QuantAnalysis (Bruker Daltonics, Bremen, Germany) software. To quantify target compounds, exact (± 10 mDa) ion chromatograms (EICs) for each of the target compounds were extracted from the three dimensional LCMS data of each sample and calibration standard. The concentrations of components in samples were calculated by comparison to external calibration curves of authentic compounds. When an authentic compound was not available, the calibration curve of a similar compound was used to calculate equivalents. For example all anthocyanins were quantified as cyanidin 3-glucoside equivalents.
